# Predicting EuroQol (EQ-5D-5 L) health state utilities from functional outcomes of sleep questionnaire (FOSQ-10) scores

**DOI:** 10.1007/s10198-025-01885-1

**Published:** 2026-01-31

**Authors:** Andrea N. Natsky, Ching Li Chai-Coetzer, Andrew Vakulin, Billingsley Kaambwa

**Affiliations:** 1 Menzies Centre for Health Policy and Economics, Faculty of Medicine and Health, University of Sydney, Sydney, NSW Australia; 2 College of Medicine and Public Health, Adelaide Institute for Sleep Health, FHMRI Sleep Health, Flinders University, Adelaide, SA Australia; 3 Respiratory and Sleep Services, Southern Adelaide Local Health Network, SA Health, Adelaide, SA Australia; 4 Flinders Health and Medical Research Institute, College of Medicine and Public Health, Flinders University, Adelaide, South Australia Australia

**Keywords:** Mapping, FOSQ-10, EQ-5D-5L, OSA, Sleep health, Quality-of-life instruments, C2, I1

## Abstract

Utility-based quality-of-life measures are generally recommended for economic evaluation. However, disease-specific, non-utility outcome measures are used more frequently than the former, particularly in sleep health studies. This paper aimed to establish a mapping algorithm between two of the most used instruments in sleep health and the general population, respectively: the Functional Outcomes of Sleep Questionnaire 10 items (FOSQ-10) and the EuroQoL 5 Dimensions 5 Level (EQ-5D-5 L). In-sample cross-validation approach was performed using a k-fold technique to randomly divide the primary dataset (*n* = 1,514) into ten subsamples. Five regression techniques were employed to estimate utilities from FOSQ-10, including ordinary least squares, censored least absolute deviations, generalised linear model (GLM), GLM inverse and beta-binomial models. Six criteria: mean absolute error (MAE), root mean squared error (RMSE), correlation distribution of predicted utilities, residual distributions and proportion of predictions with absolute errors of less than 10% and 25% were used to evaluate the predictive ability of 55 regression models. Beta Binomial model 9 (BB (9)) was chosen as the best-fitting model based on the selection criteria rankings. The BB (9) employed a multivariable fractional polynomial technique, where all significant FOSQ-10 domains, obstructive sleep apnoea (OSA) diagnosis status, income and education level were included as continuous variables. RMSE (0.1997) and MAE (0.1433) estimates for this model were within the range of published estimates. When data on utility-based measures have not been collected directly, the mapping algorithm can be utilised to predict utility where only FOSQ-10 data are available.

**Supplementary Information:**

The online version contains supplementary material available at https://doi.org/10.1007/s10198-025-01885-1.

## Introduction

Obstructive sleep apnoea (OSA) is a prevalent sleep-related breathing disorder affecting one-seventh of the general adult population globally [1]. The most notable clinical symptoms of OSA are excessive daytime sleepiness, fatigue and lack of energy experienced by individuals. Due to chronic sleep disruption or recurrent hypoxemia [2], OSA is also associated with several adverse health consequences, including cardiovascular and neuropsychiatric sequelae [3], and elevated risks of occupational and traffic accidents [4]. The detrimental effect of OSA on physical and mental functioning also extends to the disruption of professional and social lives, with evidence that OSA patients have increased absenteeism, presenteeism and more significant health services utilisation [5, 6], which ultimately impact health-related quality of life (HRQoL) of persons with OSA [7].

Given the high prevalence of OSA and its negative health consequences, it is vital to quantify the HRQoL related to OSA to help better define the burden of disease and develop more efficient sleep health services. Various instruments are now available for clinicians and researchers to measure the HRQoL in individuals with OSA, which can be divided into sleep-specific and generic instruments. Various sleep-specific instruments exist, including, but are not limited to, the Calgary Sleep Apnoea Quality of Life Instruments, Functional Outcomes of Sleep Questionnaire (FOSQ), OSA Patient-Oriented Severity Index, Maugeri obstructive sleep apnoea syndrome questionnaire and Quebec sleep questionnaire. Generic instruments relevant to OSA populations include the Medical Outcome Survey Short Form-36 (SF-36), the Nottingham Health Profile, Sickness Impact Profile (SIP), and the EQ-5D.

As the current healthcare system is faced with finite resources, economic evaluation is integral in determining the value for money of relative interventions and services, particularly for OSA management. The use of economic evaluation, particularly cost-utility analysis (CUA), is also endorsed by decision-making bodies internationally, including the Pharmaceutical Benefits Advisory Committee and the Medical Benefits Advisory Committee in Australia, Canadian Agency for Drugs and Technologies in Health and the National Institute for Health and Care Excellence in the United Kingdom [8–10]. One key component in undertaking CUA is assessing the utility of the population of interest, mainly reported as the quality-adjusted life years (QALY), which provides a standardised currency for assessing the multiple dimensions of health benefits in terms of quality of life and survival gained [11]. Instruments specific for disorders of excessive sleepiness (e.g. FOSQ) are more commonly used within this disease area but unsuitable for CUA. Therefore, an algorithm enabling mapping from sleep-specific instruments to utility scores, which can then be used within CUA, would be helpful in instances where data on only the former have been collected [12, 13].

This study aims to develop a regression-based mapping function to predict utilities based on the EQ-5D-5 L [14] from a disease-specific instrument, the Functional Outcomes of Sleep Questionnaire 10 items (FOSQ-10) [15]. Data for both HRQoL instruments were collected as part of a survey of diagnosed and at-risk OSA who participated in a study exploring the views and preferences regarding OSA management pathways [16]. Each disease-specific instrument provides unique aspects of health outcomes and quality of life [17–19]. The FOSQ is the first self-report tool to evaluate the impact of daytime sleepiness on activities of daily living [20], is commonly used in sleep research [19], and has demonstrated positive psychometric performance and responsiveness, particularly in populations with OSA [18]. However, the FOSQ-10, a shorter test version, is used instead of the original 30-item version. The FOSQ-10 has similar psychometric performance as the longer version and is significantly less laborious and time-consuming for participants to complete. The EQ-5D-5 L is a generic HRQoL instrument relevant for OSA populations to generate utility scores from the FOSQ-10 as it is the most-used preference-based tool to generate single utility scores associated with health states [21]. To our knowledge, no mapping algorithms have been previously developed between the two instruments. The study findings are designed to contribute to existing knowledge regarding the application of these instruments in populations with OSA, as well as broadening the availability of utility-derived scores from a disease-specific instrument.

## Methods

We adhered to the ‘Mapping onto Preference-Based Measures Reporting Standards’ checklist [22] to predict utilities from the FOSQ-10 (source instrument) to the EQ-5D-5 L (target instrument).

###  Data

Participants were general population ≥ 18 years of age recruited from an online panel of Australian adults by Dynata, a survey and market research company (Melbourne, Australia). Dynata conducted the survey in October 2019 using invitations through various platforms, including email, telephone alerts, banners and online messaging. A three-stage randomisation process is used to match a potential participant with a survey they are likely to complete and minimise the risk of bias [23]. A wide selection of participants was purposefully drawn from Dynata’s panel to maximise the variation of respondents’ characteristics. OSA-50 screening questionnaire was used in the survey to identify participants diagnosed with or at high risk for OSA based on a score of ≥ 5 out of a maximum of 10 [24]. Specific project details were not included in the invitation to avoid self-selection bias. The panel was sampled to generate a study sample that matches the Australian Bureau of Statistics estimates on sex, state and regional representation. The survey was approved by the Human Research Ethics Committee at Flinders University (Project 8435). The survey comprises sections on demographics, diagnosis status, OSA symptoms, patient satisfaction and motivations for OSA treatment and services, treatment options, treatment compliance, discrete choice experiment on OSA management pathways and general health and HRQoL.

### Source and target measures

The HRQoL was measured using two instruments, namely EQ-5D-5 L as the target measure and the FOSQ-10 as the source measure.

The FOSQ-10 is a self-report functional status instrument that measures the impact of sleepiness on activities of daily living and quality of life and commonly used in sleep research [15, 19]. It is a non-preference-based, disease-specific HRQoL instrument that was not specifically created for application in economic evaluations. The FOSQ-10 is a shorter version of the original 30-item FOSQ [20] that performs similarly to the longer form and is more easily administered in research and clinical practice settings. It consists of 10 items with five subscales: general productivity, activity level, vigilance, social outcome, and sexual relationships and intimacy. Each item has a 4-point Likert response format with a higher score reflecting improvements in HRQoL (e.g., 1 = extreme difficulty, 2 = moderate difficulty, 3 = a little difficulty, and 4 = no difficulty). A response alternative is also available, represented by a score of 0, which implies a missing response or if participants do not engage in the activity for reasons other than tiredness or sleepiness. The item scores can be summed up to calculate a mean-weighted item score, particularly for subscales with more than one item. Once the means of each subscale score have been computed and multiplied by five, the total score is obtained, ranging from 0 to 20, with higher scores indicating better sleep-specific HRQoL.

The EQ-5D-5 L [14] is an HRQoL instrument commonly used in the adult population and preferred in economic evaluation studies [8]. It is a generic, preference-based instrument comprised of five single-item dimensions of health: self-care, mobility, usual activities, anxiety/depression and pain/discomfort [25]. It is a newer model derived from the original EQ-5D-3 L, which includes five rather than three Likert scales of impairment in each domain: 1 = no, 2 = slight, 3 = moderate, 4 = severe 5 = extreme [26]. With the level expansions, EQ-5D-5 L can identify 3,125 unique health states, with utility scores ranging from − 0.594 to 1 using a UK value set [27]. Scores less than 0 represent health states considered worse than death [27].

### Statistical analysis

The statistical analysis was conducted using STATA version 16 [28] in two stages: Initially, the degree of correlation between individual dimensions and utilities/summary scores of comparator instruments was examined using Spearman’s correlation, along with the distribution of the mean of each instrument across all dimension levels. Correlation analysis was conducted to determine the strength and direction of the relationship between the two HRQoL instruments, specifically how changes in FOSQ-10 correspond with changes in EQ-5D-5L. Spearman’s correlation was used because HRQoL data are commonly not normally distributed, which also occurred in this study. The large sample size in this study also justifies the use of Spearman’s correlation. A moderate correlation is established between 0.4 and 0.6, while a score of ≥ 0.70 indicates a strong correlation [29]. Based on previous literature [17], moderate to strong correlations may be predicted between subscales or dimensions that measure similar constructs, e.g. ‘activity level’ in FOSQ-10 and the ‘usual activities’ in EQ-5D-5 L dimensions. However, these associations may be negative as both instruments possessed opposite scales, where FOSQ-10 scales in decreasing severity, while EQ-5D-5 L in increasing severity. Lower mean utilities or summary scores are also anticipated for EQ-5D-5 L and FOSQ, respectively, related to increasing severity levels on the comparator instrument’s dimensions.

#### Modelling approaches

Initially, only eight model specifications were adopted in the analysis (see Table 1, model 1 to 8). As the preferred mapping model selection is an iterative process [30, 31], a further search into literature to identify an appropriate or improved regression model was conducted. Three other model specifications were incorporated into the analysis based on significant variables drawn from Least Absolute Shrinkage and Selection Operator (Lasso) Regression [32] and Multivariable Fractional Polynomial (MFP) Models [33] (see Table 1, Model 9 to 11). The two techniques are often used to select the most important predictor variables from a pool of independent variables. Lasso, by shrinking some coefficients to zero, effectively excludes less important variables, thus enhancing model simplicity and interpretability. MFP, by exploring a range of fractional polynomial transformations, identifies the best-fitting functional forms for continuous predictors, allowing for flexible modelling of non-linear relationships. The estimation samples (k = 10) were used to construct 55 primary models (derived from five regression techniques x eleven model specifications) as specified in Table 1. These primary models were then tested on 10 validation samples to evaluate their predictive ability.

**Table 1:** Variables used in regression models

Model type	Model specification
	Independent variables
1 OLS, CLAD, BB, GLM, GLM inverse	FOSQ-10 total scores only^a^
2 OLS, CLAD, BB, GLM, GLM inverse	FOSQ-10 total scores^a^, OSA^b^ and income^c^
3 OLS, CLAD, BB, GLM, GLM inverse	FOSQ-10 total scores^a^ and OSA^b^
4 OLS, CLAD, BB, GLM, GLM inverse	FOSQ-10 total scores^a^ and income^c^
5 OLS, CLAD, BB, GLM, GLM inverse	FOSQ-10 total scores^a^, FOSQ-10 total scores^a^ squared, OSA^b^ and income^c^
6 OLS, CLAD, BB, GLM, GLM inverse	FOSQ-10 total scores^a^, FOSQ-10 total scores^a^ cubed, OSA^b^ and income^c^
7 OLS, CLAD, BB, GLM, GLM inverse	FOSQ-10 domain scores^d^
8 OLS, CLAD, BB, GLM, GLM inverse	FOSQ-10 domain scores^d^, OSA^b^ and income^c^
9 OLS, CLAD, BB, GLM, GLM inverse	MFP version of model specification (8)^e^
10 OLS, CLAD, BB, GLM, GLM inverse	MFP version of model specification (8)^f^
11 OLS, CLAD, BB, GLM, GLM inverse	Lasso version of model specification (8)^g^
**Model type**	**Dependent ** **variables**
1–11	EQ-5D-5 L utilities ^i^

*EQ-5D-5 L EuroQoL 5 dimensions 5 levels*,* FOSQ-10 Functional Outcomes of Sleep Questionnaire*

^a^ FOSQ-10 is calculated as a continuous variable with a range of 0–20

^b^ OSA: dummy = 1 if diagnosed with OSA and 0 if undiagnosed

^c^ Income: dummy = 1 if earning more than AU 60,000/year

^d^ FOSQ-10 domain scores (F_gp_conc: concentration, F_gp_rem: remembering, F_al_ra: relationship affected, F_al_am: morning activity, F_al_pm: evening activity, F_vi_sd: driving short distance, F_vi_ld: driving long distance, F_vi_mo: watching movies, F_social: social visit, F_sex: desire intimacy) are scored as categorical variables with five possible values ranging from 0 to 4

^e^ Multivariable fractional polynomial model selection of model (8), includes: FOSQ-10 total score^− 2^−0.33, (FOSQ 10 total score + 4.77^e7^)/10)^−0.5^−0.76, income ^c^, OSA ^b^, education ^h^, F_gp_conc^3^−79.65, F_al_am-3.35, F_al_pm-3.26, F_sex-2.66

^f^ Multivariable fractional polynomial model selection with indicator for categorical variables of model (8), includes: FOSQ-10 total score^− 2^−0.33, FOSQ-10 total score^− 0.5^−0.76, income ^c^, OSA ^b^, education^h^, F_gp_conc-3.35, F_al_ra-3.66, F_al_am-3.39, F_al_pm-3.31, F_social^− 2^−0.07, F_sex-3.30

^g^ Multivariable fractional polynomial model selection of model (8), includes: FOSQ10 1.F_gp_conc 4.F_gp_conc 1.F_gp_rem 1.F_al_ra 4.F_al_am 1.F_al_pm 4.F_al_pm 1.F_vi_sd 2.F_vi_ld 3.F_vi_mo 1.F_social 4.F_social 4.F_sex 0.biincomelevel 0.OSA

^i^ EQ-5D-5 L utilities are calculated as continuous variables ranging from − 0.594 to 1, except for Beta regression, where zeros replace negative values

^h^ Education: dummy = 1 if attaining education higher than high-school/Year 12 level and 0 if not finished high-school/Year 12 level

Using the regression models, the chosen independent variables from FOSQ-10 were mapped onto EQ-5D-5 L. Specific respondent characteristics were also included when they enhanced the predictive capacity of the models. We also performed sensitivity analyses to test the effect of having dummy variables in the models. The following are the initially planned regression model families to be used in the mapping:

- *Ordinary least squares (OLS) regression model* The most commonly used regression in mapping [34], yet its limitations include unreliable coefficient estimates, out-of-range predictions and ceiling effects [35]. Out-of-range predictions occur when the model forecasts implausible values outside the feasible utility range, which, to a certain scale, can be illogical for HRQoL. Ceiling effects happen when the model’s predictions reach an upper limit and cannot exceed it, leading to a clustering of predictions at the top end.
- *Censored least absolute deviations (CLAD)* A consistent and asymptotically normal model for a broad class of error distribution, which also accounts for the ceiling effect and is robust when used for heteroscedastic and skewed data [36].
- *The generalised linear model (GLM)* A robust model for heteroscedasticity and skewed distribution, and the choice of using this technique is guided by Manning’s modified park test [37].
- *The Beta Binomial (BB) regression model* The BB model can robustly compute unimodal or bimodal utilities despite skewness. However, it has a strict range of 0 to 1 for utility scores [38]. Apart from a small proportion (1.98%) of observations for the EQ-5D-5 L, most utilities (*n* = 1,484) in our data set were positive. As done elsewhere, these outliers were set equal to zero [39].

#### Estimation and validation of primary models

Complete datasets were available for both instruments and the relevant demographic data. Hence no data imputation was required. The in-sample cross-validation approach was performed using a k-fold technique to randomly divide the primary dataset (*n* = 1,514) into k subsamples (k = 10). One subsample was retained as the validation data for testing the predictive model, and the remaining nine subsamples were used as training or estimation data [40]. This method produced a less biased estimate than a simple train/test split. The cross-validation process was repeated ten times. Each time, one of the *k* subsets was used exactly once as the validation set, and the other *k-1* subsets were placed together to form the estimation sample [40]. The average error across all *k* trials can then be computed. A value of k = 10 is recommended and is commonly used; thus, it was adapted in this study [41].

#### Assessment of predictive ability

Using STATA’s inbuilt post-estimation commands, predictive utilities from all 1100 cross-validated models were estimated. The predictive ability of the models was examined using two measures of predictive error [41]: the root mean squared error (RMSE) and the mean absolute error (MAE), with lower values of the measures indicating a better-performing predictive model. As done elsewhere and suggested by past literature, more weight was placed on the RMSE if inconsistent results were generated by the RMSE and MAE, particularly when errors from the model had a Gaussian distribution [42]. Model performance was assessed initially in the validation, then the estimation sample. Additional assessments were imposed to evaluate the performance of the models further. First, Spearman’s rank correlation between the observed and predicted utilities was used to investigate the level of agreement between the two values. Second, the observed and predicted utility distributions were examined to determine how closely both scores matched each other [39, 43]. Third, the residuals (observed minus predicted utilities) were assessed to check for bias in the predicted utilities [39, 43]. Lastly, the proportion of predicted utility scores that deviated from the observed value by no more than 0.10 or 0.25, respectively, were reported to determine the difference between predicted and observed scores [44].

## Results

### Demographics and other participant characteristics

Table 2 displays the participants’ characteristics. From 3,812 individuals, completed surveys were returned by 1,514 participants diagnosed with or at high risk for OSA. Almost half of the study sample was male (*n* = 732, 48%). The majority of the sample was made up of people who were aged 45 to 74 years old (*n* = 1,155, 76%), educated beyond high school (*n* = 1,004, 67%), living in a metropolitan area (*n* = 981, 65%) and indicated that their general health was excellent, very good or good (*n* = 864, 56%). The mean (standard deviation) for EQ-5D-5 L and FOSQ-10 for the entire sample was 0.709 (0.244) and 17.401 (2.952), respectively. By transforming these results into Z scores, the mean scores were standardised toa mean of 0 and a standard deviation of 1. This transformation allows for effective comparison between the instruments, as it places them on a common scale. Despite this standardisation, the median scores varied, ranging from 0.176 to 0.316, suggesting certain degrees of variation between the instruments.

**Table 2:** Quality of life (EQ-5D-5 L and FOSQ-10) values for selected patient characteristics

Characteristics	*N*(%)	EQ-5D-5 L				FOSQ-10			
		Mean	SD	Median	IQR	Mean	SD	Median	IQR
Whole sample	1516 (100%)	0.709	0.244	0.752	(−0.453–1.453)	17.401	2.952	18.333	(5–20)
Whole sample (Z scores)	1516 (100%)	0	1	0.176	(−4.768 - 1.193)	0	1	0.316	(−4.20-0.88.20.88)
Age groups (n, %)									
18–24	27 (2%)	0.644	0.304	0.740	(−0.255–1.255)	16.054	2.968	16.333	(12.167–19.167)
25–34	100 (7%)	0.663	0.312	0.757	(−0.013–1.013)	15.219	3.874	16	(7.332–20.332)
35–44	202 (13%)	0.689	0.228	0.740	(0.075–1.075)	16.258	3.027	16.667	(8.125–20.125)
45–54	350 (23%)	0.690	0.268	0.750	(−0.114–1.114)	16.839	3.026	17.667	(7.5–20)
55–64	398 (26%)	0.713	0.235	0.767	(−0.042–1.042)	17.825	2.800	18.75	(7.5–20)
65–74	407 (27%)	0.739	0.214	0.767	(−0.053–1.053)	18.556	2.036	19.333	(10.333–20.333)
75+	32 (2%)	0.814	0.133	0.826	(0.668–1.668)	18.767	1.618	19.25	(17.083–20.083)
*p*-value				0.037				0	
Gender (n, %)									
Male	732 (48%)	0.722	0.255	0.767	(−0.453–1.453)	17.609	2.937	18.667	(5–20)
Female	482 (52%)	0.697	0.232	0.750	(−0.099–1.099)	17.225	2.938	18.333	(7.167–20.167)
Other^1^	2 (0%)	0.495	0.386	0.495	(0.222–0.768)	10.583	2.711	10.583	(8.67–12.5)
p-value				0.012				0	
Education (n, %)									
Year 8 or below	24 (2%)	0.648	0.28	0.717	(0.429–0.879)	16.743	4.598	18.417	(14.833–19.5)
Year 9	44 (3%)	0.634	0.241	0.708	(0.230–0.906)	17.757	2.295	18.521	(13–20)
Year 10	159 (10%)	0.658	0.266	0.725	(−0.025–1.025)	17.507	2.927	18.500	(8.75–20.75)
Year 11	64 (4%)	0.715	0.234	0.767	(0.230–1.230)	18.069	2.367	18.708	(12.667–20.667)
Year 12	218 (14%)	0.718	0.211	0.767	(0.168–1.168)	17.770	2.766	18.708	(7.5–20)
Certificate	327 (22%)	0.679	0.266	0.736	(−0.116–1.116)	17.071	3.035	18.333	(8–20)
Diploma	223 (15%)	0.747	0.21	0.768	(0.036–1.036)	17.661	2.696	18.667	(9.833–20.833)
Undergraduate and above	454 (30%)	0.735	0.242	0.768	(−0.452–1.452)	17.202	3.103	18.125	(5–20)
Prefer not to answer	3 (0%)	0.805	0.070	0.796	(0.740–0.879)	17.722	0.734	18.125	(17.875–18.167)
*p*-value				0				0.052	
Income level (n, %)		Mean	SD	Median	IQR	Mean	SD	Median	IQR
<$12,000	158 (10%)	0.743	0.242	0.768	(−0.028–1.028)	17.924	2.944	19.000	(8.125–20.125)
$12,001 - $20,000	49 (3%)	0.549	0.260	0.570	(0.129–0.848)	16.531	3.079	17.333	(11–20)
$20,001-$30,000	203 (13%)	0.624	0.263	0.681	(−0.042–1.042)	17.509	2.828	18.333	(8.333–20.333)
$30,001-$40,000	142 (9%)	0.667	0.243	0.735	(0.026–1.026)	17.526	2.780	18.333	(10.167–20.167)
$40,001-$50,000	124 (8%)	0.720	0.210	0.759	(0.18–1.18)	17.493	2.942	18.8125	(10–20)
$50,001-$60,000	130 (9%)	0.736	0.221	0.767	(−0.016–1.016)	17.765	2.413	18.521	(11.333–20.333)
$60,001-$80,000	187 (12%)	0.715	0.230	0.763	(0.006–1.006)	17.308	3.049	18.500	(8.333–20.333)
$80,001-$100,000	185 (12%)	0.738	0.264	0.768	(−0.013–1.013)	17.159	3.175	18.167	(6.667–20.667)
$100,001-$150,000	199 (13%)	0.750	0.199	0.768	(0.168–1.168)	17.071	3.097	18.000	(8.167–20.167)
$150,001-$200,000	84 (6%)	0.759	0.207	0.768	(0.216–1.216)	17.639	2.277	18.229	(12.667–20.667)
>$200,000	55 (4%)	0.754	0.313	0.837	(0.218–1.218)	17.108	3.656	18.667	(10–20)
*p*-value				0				0.013	
Location (n, %)									
Metro	981 (65%)	0.718	0.24	0.767	(−0.453–1.453)	17.335	2.926	18.333	(5–20)
Regional	535 (35%)	0.693	0.25	0.74	(−0.116–1.116)	17.523	2.999	18.542	(7.33–20.33)
*p*-value				0.06				0.052	
General health (n, %)									
Excellent	45 (3%)	0.762	0.335	0.848	(0.255–1.255)	17.229	3.836	19	(10.83–20.83)
Very Good	250 (16%)	0.814	0.837	0.837	(0.057–1.057)	18.246	2.639	19.333	(8.5–20)
Good	569 (37%)	0.791	0.164	0.768	(0.001–1.001)	18.058	2.398	18.958	(8.333–20.333)
Fair	467 (31%)	0.653	0.222	0.71	(−0.114–1.114)	17.139	2.709	18	(7.67–20.67)
Poor	184 (12%)	0.442	0.275	0.456	(−0.099–0.906.099.906)	14.945	3.744	15.333	(6.83–20.83)
Unsure	1 (0%)	0.508		0.508		15.417		15.417	
*p*-value				0				0	

^1^two respondents with gender registered as ‘other’ were excluded from the test of difference analysis due to small number of samples

### Bivariate relationship between FOSQ-10 and EQ-5D-5 L

Table 3 summarises the relationships between the individual dimensions of both instruments and between individual dimensions of one instrument relative to utilities/summary scores for each comparator instrument. Correlation between other individual dimensions between EQ-5D-5L and FOSQ-10 was relatively low, ranging from − 0.51 to −0.14. The negative association between the individual dimensions of the quality-of-life instruments is due to differences in the descriptive scoring system adopted by both instruments, where FOSQ-10 has its domain subscales in decreasing severity, while those for the EQ-5D-5L are in increasing severity. However, both instruments’ utility and summary scores correlate positively with higher scores, indicating better quality of life. The most robust evidence of convergent validity between dimensions was seen in comparing ‘anxiety/depression’ (EQ-5D-5L) and the summary scores of FOSQ-10 (r=−0.53). There was evidence of moderate convergent validity between the EQ-5D-5L’ anxiety/depression’ domain and other similar dimensions of FOSQ-10, i.e., general productivity (r=−0.50) and activity level (r=−0.51). Positive moderate associations were evident between EQ-5D utility scores and some of the FOSQ-10 individual dimensions, such as general productivity (r = 0.41), activity level (r = 0.46) and overall summary score (r = 0.48). These findings show that correlations between all dimensions measuring similar constructs were in the direction that was hypothesised (negative for individual dimensions and positive for summary/utility scores). However, the level of correlation was low or moderate instead of strong. Table 3 also demonstrates that overall, higher correlations were apparent between dimensions of particular instruments and utility scores of comparator instruments than those observed between individual dimensions of comparator instruments. As expected, positive correlation between mean utilities of EQ-5D-5L and summary scores of FOSQ-10 was observed with lower scores in increasing severity levels and vice versa.

**Table 3:** Correlation between quality-of-life instrument dimensions and utilities/summary scores

	EQ-5D5L dimensions					
FOSQ10 dimensions	Mobility	Self-care	Usual Activities	Pain/discomfort	Anxiety/depression	EQ-5D5L utility score
General productivity	−0.207	−0.247	−0.289	−0.299	−0.496	0.414
Activity level	−0.255	−0.297	−0.351	−0.324	−0.506	0.461
Vigilance	−0.204	−0.263	−0.276	−0.250	−0.350	0.335
Social outcomes	−0.245	−0.325	−0.323	−0.228	−0.331	0.331
Intimacy and Sexual relationships	−0.186	−0.139	−0.217	−0.211	−0.284	0.294
FOSQ10 summary score	−0.264	−0.316	−0.347	−0.332	−0.533	0.476

### Assessment of model predictive ability

Table S1 displays the summary performance indicators for each of the 1,100 models when assessed using the cross-validated estimation (550 models) and validation samples (550 models). The table ranks each model and checks for consistencies between the estimation and validation samples to select the five best-fitting models. GLM B (Inverse) and CLAD models consistently over-predicted EQ-5D-5 L utility scores, while the rest (OLS, BB and GLM) predicted values equivalent to or within 0.01 points of the observed EQ-5D-5 L scores. The range of the predicted EQ-5D-5 L scores (minimum to maximum) for models in both estimation and validation samples was narrower than that for the observed utilities. Performance indicators were computed using these models (with the range of values shown in parentheses for the average of cross-validated estimation and validation samples, respectively): RMSE (0.1997–0.2175 and 0.1898–0.2147), MAE (0.1433–0.1578 and 0.1430–0.1539), correlation (0.490–0.581 and 0.429–0.553), and the proportion of predictions where the predicted utility score deviated from the observed value by < 0.10 (43–53% and 42–50%) or < 0.25 (80–85% and 81–86%).

The RMSE was used initially to rank the performance of the 550 models in the validated sample resulting in the five best-predicted models: BB (10), BB (9), BB (11), BB (8) and BB (5). The performance indicators drawn from these models (range of values again presented for the estimation and validation samples, respectively) were: RMSE (0.1898–0.1937 and 0.1997–0.2041), MAE (0.1433–0.1496 and 0.1430–0.1458), correlation (0.553–0.565 and 0.484–0.523), and the proportion of predictions where the predicted utility score that deviated from the observed value by < 0.10 (45–50% and 43–46%), < 0.25 (80–84% and 83–84%). When all performance indicators were considered in the validation sample (Table 4), BB (10) performed best on the RMSE and second-best on the MAE. The BB (9) performed second-best on RMSE but best for the MAE in the validation sample. Compared to all models in the validation samples, although they perform best in RMSE and MAE, all the BB models did not score in the top ten in terms of correlation and proportion deviated from the observed values. A further examination of the measures of spread (particularly the 25th percentile, median, and 75th percentile) did not clearly indicate which model predicted utilities more closely to the observed scores and the actual distribution.

**Table 4:** Predictive ability of the best-fitting models

Estimation method^a^	Mean (SD)	Min.	*P*.25	Median	*P*.75	Max.	Corr	RMSE	MAE	% <0.10	% <0.25
Estimation sample (*n* = 1362)*											
Observed EQ-5D-5 L	0.709 (0.247)	−0.594	0.635	0.761	0.846	1					
BB (9)	0.705 (0.123)	0.229	0.641	0.738	0.793	0.913	0.553	0.19971	0.1433	50%	80%
OLS (9)	0.709 (0.137)	−0.002	0.646	0.742	0.804	0.988	0.564	0.20169	0.1487	46%	84%
OLS (10)	0.709 (0.137)	−0.005	0.646	0.740	0.811	0.945	0.564	0.20206	0.1470	46%	84%
OLS (7)	0.709 (0.137)	−0.088	0.638	0.741	0.826	0.925	0.564	0.20411	0.1484	45%	84%
GLM (8)	0.709 (0.144)	0.120	0.635	0.733	0.817	1.135	0.565	0.20413	0.1496	47%	84%
Validation sample (*n* = 152)*											
Observed EQ-5D-5 L	0.709 (0.242)	−0.274	0.627	0.757	0.847	1					
BB (10)	0.708 (0.121)	0.298	0.639	0.739	0.802	0.870	0.488	0.1898	0.1439	45%	84%
BB (9)	0.708 (0.118)	0.293	0.643	0.740	0.798	0.865	0.484	0.1902	0.1430	46%	84%
BB (11)	0.704 (0.118)	0.332	0.622	0.735	0.801	0.854	0.523	0.1924	0.1456	44%	84%
BB (8)	0.705 (0.126)	0.277	0.626	0.737	0.806	0.881	0.506	0.1936	0.1454	44%	83%
BB (5)	0.704 (0.113)	0.401	0.631	0.739	0.792	0.843	0.507	0.1937	0.1458	43%	83%

The dependent variables in all regression models were the EQ-5D-5 L (Euro-QoL 5 dimensions 5 levels) utilities. The independent variable(s) are outlined in Table 1

*Corr* Spearman correlation, *MAE* mean absolute error, *Max* maximum, *Min* minimum, *P.25* 25th percentile, *P.75* 75th percentile, *RMSE* root mean squared error, *SD* standard deviation, % <0.10 and % <0.25 total proportion of all predictions (across both the estimation and validation samples) where the predicted utility score differed from the observed value by no more than 0.10 or 0.25 respectively. Model performance was assessed using the two primary goodness-of-fit statistics: RMSE and MAE

^a^ Estimation method: *BB* beta-binomial, *OLS* ordinary least square, *GLM* generalised linear model

*In-sample cross-validation approach was performed using a k-fold technique, which randomly divide the primary dataset (*n* = 1,514) into k subsamples (k = 10) where one subsample was retained as the validation data for testing the predictive model, and the remaining nine subsamples were used as training or estimation data

In contrast to the validation sample, where BB family models dominate the five best-predicted models based on RMSE, the best predictive models in the estimation sample were a mixture of family models consisting of BB (9), OLS (9), OLS (10), OLS (7) and GLM (8). BB (9) performed best on the RMSE and MAE in the estimation sample, second-best on the proportion of predicted utility deviation less than 0.10, and seventh on the correlation. OLS (9) performs second best for the RMSE, fourth on the MAE and fifth on correlation. OLS (10) was the third-best on the RMSE, second-best for MAE and fifth on the proportion of predictions where the predicted utility score deviated from the observed value by < 0.25. Model specifications (9) and (10) performed the best among other models. Both models were categorised as non-linear models that included fractional polynomial terms (MFP). The independent variables for model specifications (9) and (10) had a combination of demographic characteristics, total score and domains of the FOSQ-10 that were deemed statistically significant as computed by the MFP models. Including dummy variables (living-area = 1 if regional, 0 metro, age = 1 if < 55 years, 0 if > 55 years, and gender = 1 if male, 0 if female) in all model specifications within the sensitivity analysis did not result in better-performing models (Table S2).

**Fig. 1:**
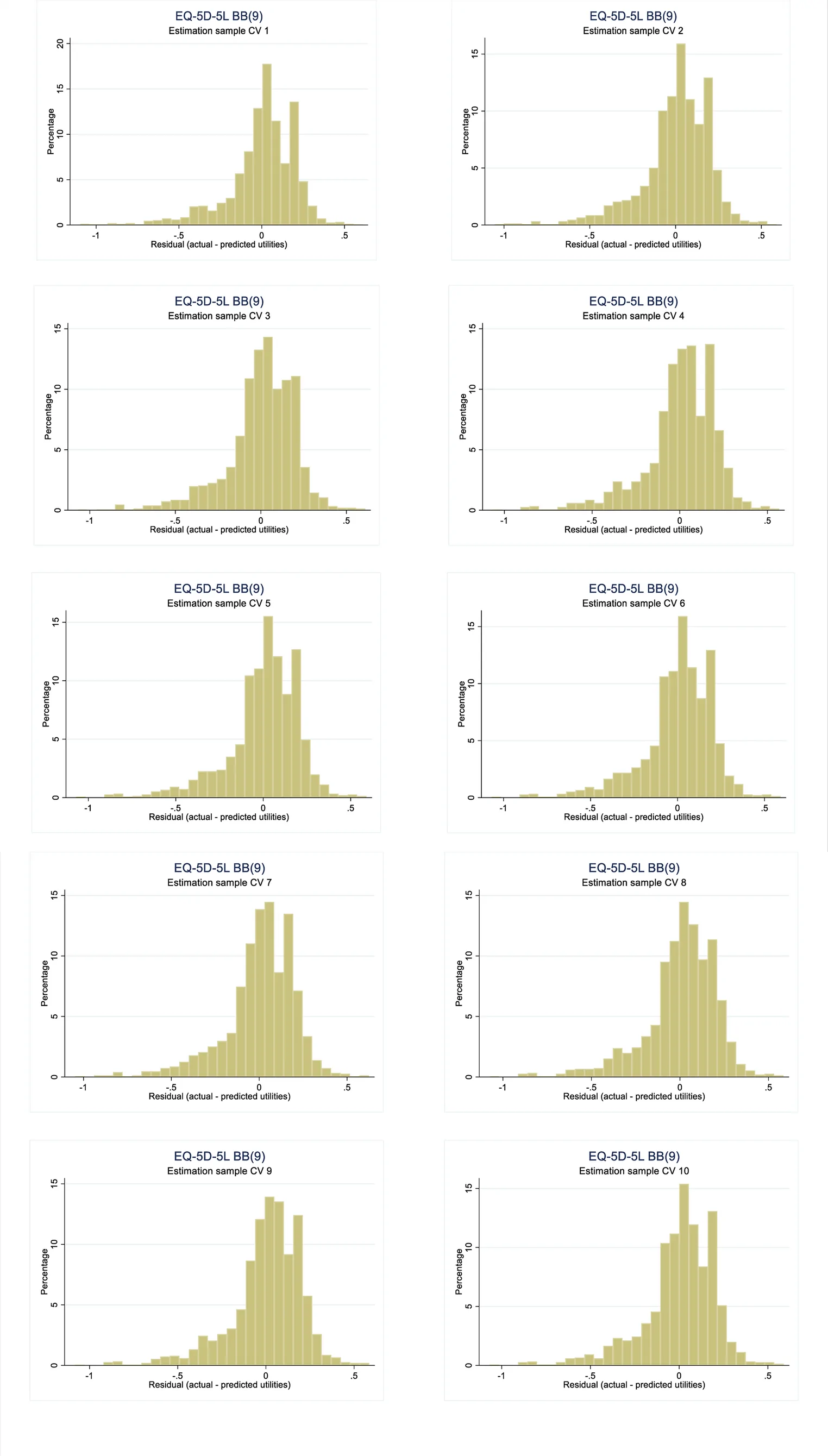
Distribution of BB (9) residuals across k=10 estimation samples

**Fig. 2:**
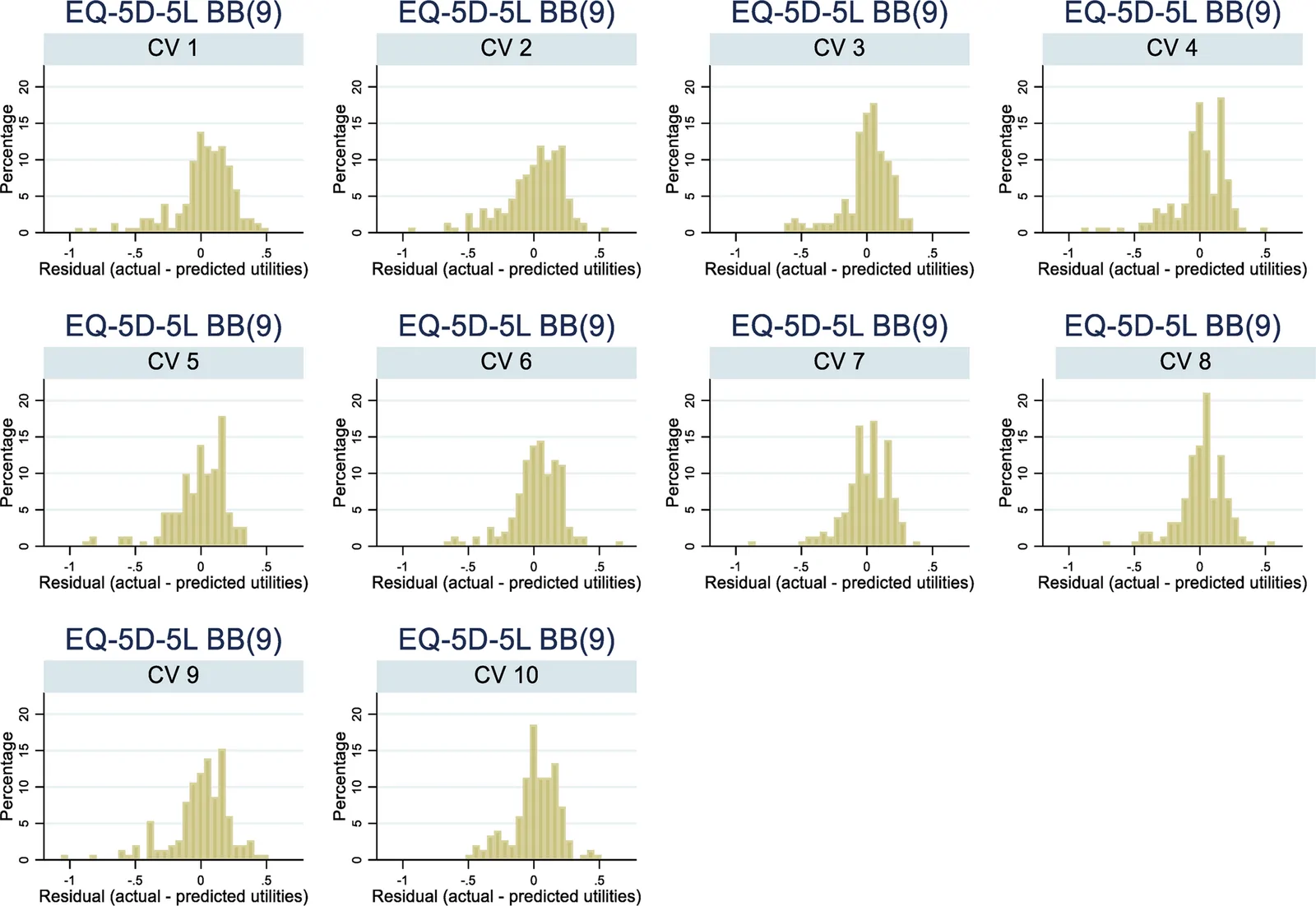
Distribution of BB (9) residuals

**Fig. 3:**
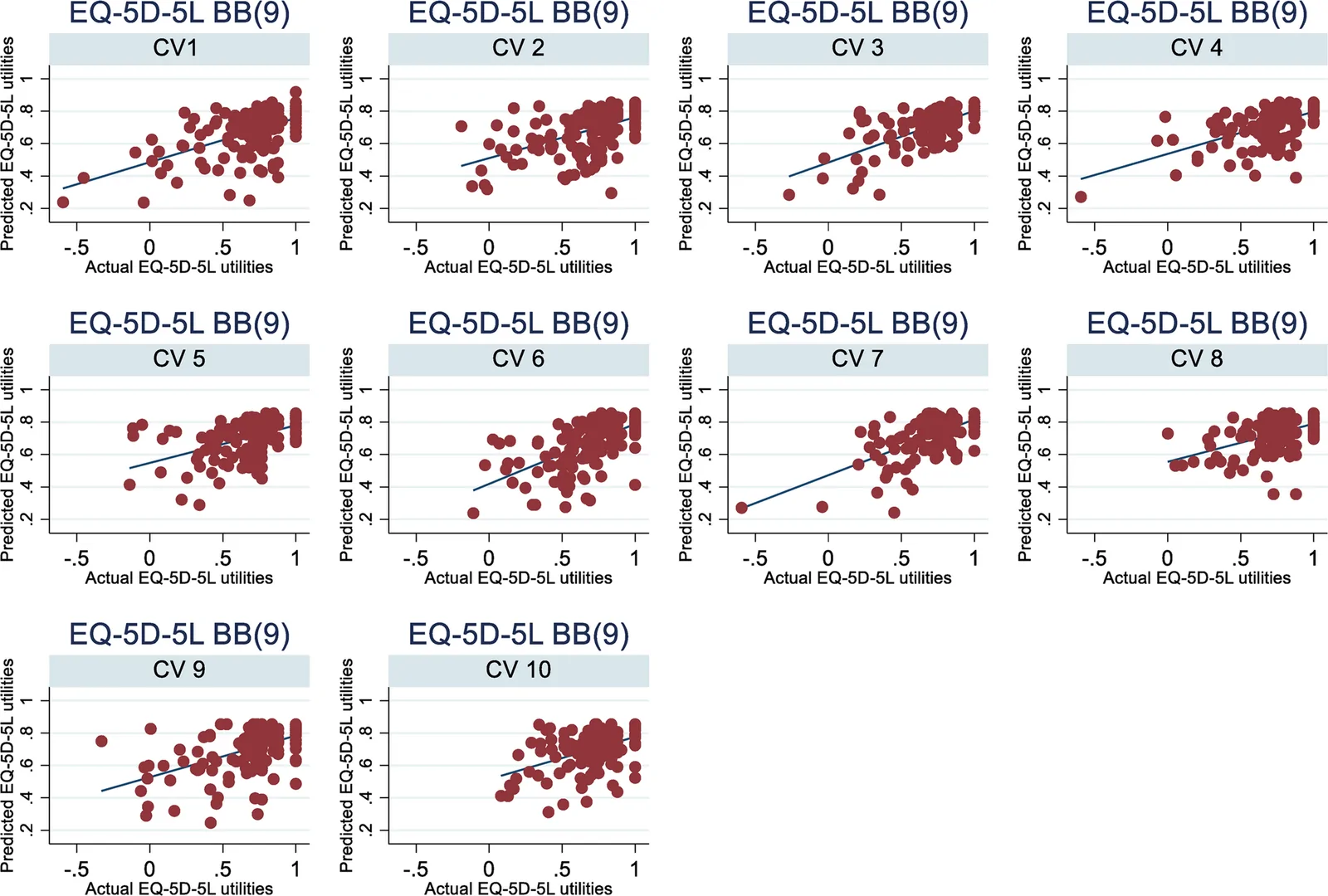
Scatter plot of predicted versus actual BB (9) EQ-5D-5L values across k=10 validation samples

**Fig. 4:**
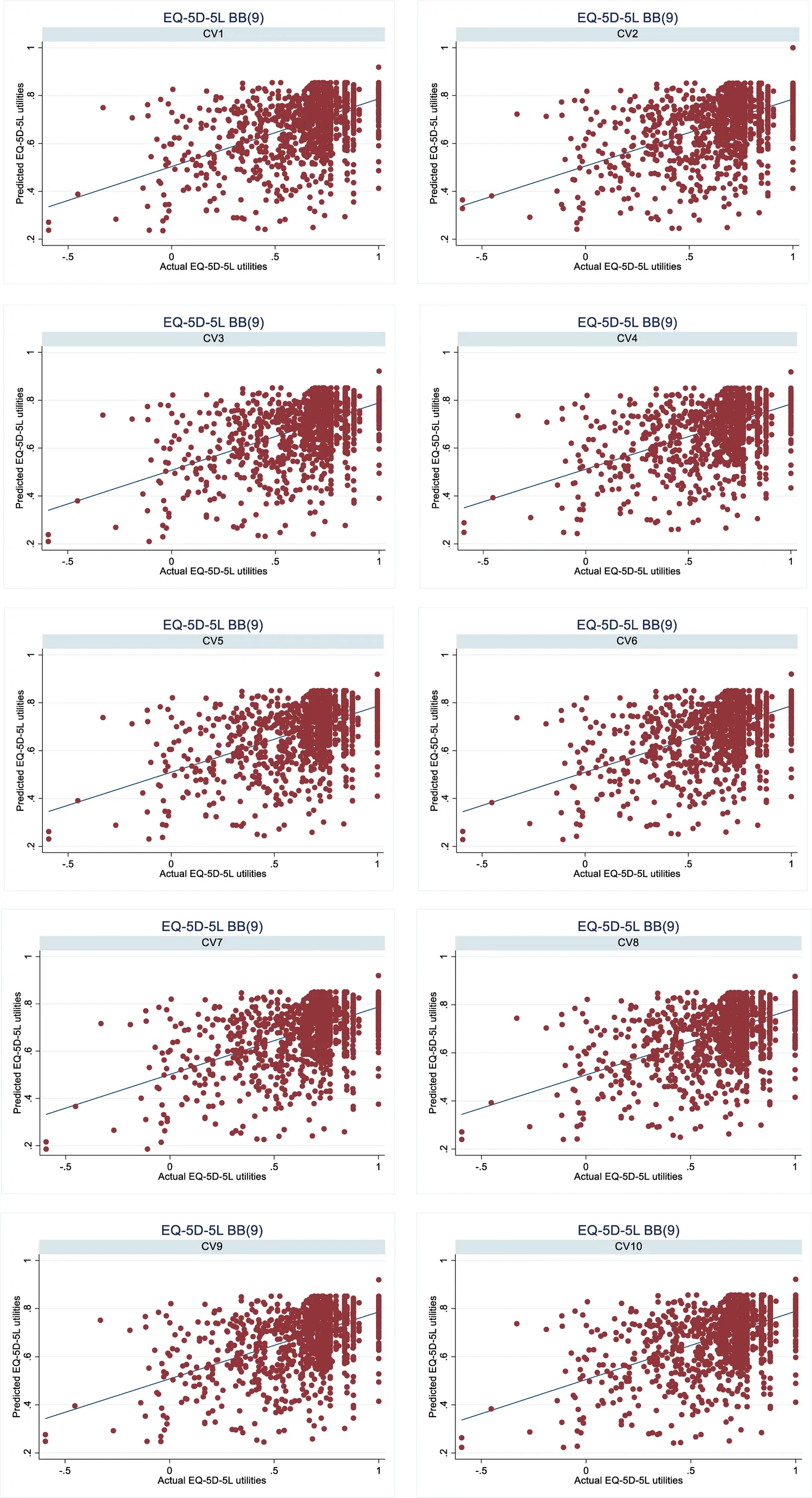
Scatter plot of predicted versus actual BB (9) EQ-5D-5L values across k=10 estimation samples

Based on the rankings on the RMSE and MAE, supplemented by the additional selection criteria, BB (9) was chosen as the best-fitting model. When considering the regression model families and model specification BB (9) was relatively the better performer in both estimation and validation samples. For BB (9), the visual inspection of the residuals in the estimation samples, as demonstrated in Fig. 1, suggests similar distribution in all cross-validated folds. However, compared with the histograms of validated samples shown in Fig. 2, the distribution is slightly more scattered due to the difference in the number of samples. Yet, the central tendencies (mean, mode, median) remain close to zero (appear close to being normally distributed) as observed in the estimated sample. Similar findings are apparent when looking at the values of the predicted EQ-5D-5 L utilities when plotted against the actual EQ-5D-5 L utilities, as presented in Figs. 3 and 4 for the validated and estimated samples, respectively. Spearman’s rank correlations between BB (9) predicted and observed utilities in both samples showed moderate correlation (0.48–0.55). The regression model coefficients for BB (9), based on running the model in the whole dataset, are presented in Table 5. The beta-binomial coefficients are not directly interpretable. However, in this study, we are not interested in interpreting the raw coefficients but rather in predicting EQ-5D-5 L utilities from FOSQ-10. Therefore, an example has been given in Table S3 on how to use the coefficients reported in Table 5 to calculate the predicted EQ-5D-5 L utilities from FOSQ-10 using the logit transformation. Additionally, the average marginal effects of each variable used in the best-performing model are provided in Table S4. To predict EQ-5D-5 L utilities from FOSQ-10 (with domains scored 1 = extreme difficulty to 5 = no difficulty), the recommended mapping algorithm is as follows:

$$\begin{array}{c}EQ-5D-5L\:utility=exp\left(0.7170+\:{(1.63}^{-11}\times\:\:Item\:1\right)-\left(1.5640\times\:\:Item\:2\right)\\\:+(0.2376\:\times\:\:Item\:3)-\:(0.1777\:\times\:\:Item\:4)+\:(0.1929\:\times\:\:Item\:5)\\+\:(0.0046\:\times\:\:Item\:6)+\:(0.1351\times\:\:Item\:7)+\:(0.1746\:\times\:\:Item\:8)\\+(0.0704\:\times\:\:Item\:9))/(1+exp(0.7170+\:{(1.63}^{-11}\times\:\:Item\:1)\:\\-\:\left(1.5640\times\:\:Item\:2\right)+(0.2376\:\times\:\:Item\:3)-\:(0.1777\:\times\:\:Item\:4)\\+\:(0.1929\:\times\:\:Item\:5)+\:(0.0046\:\times\:\:Item\:6)+\:(0.1351\times\:\:Item\:7)\\+\:(0.1746\:\times\:\:Item\:8)+(0.0704\:\times\:\:Item\:9))\end{array}$$

**Table 5:** Model coefficients for the five best-fitting models based on the whole sample (*n* = 1,514) ^a^

FOSQ-10 domain item/demographic characteristics	Estimation method [coefficient (standard error)]^b^				
	BB (9)^c^	OLS (9) ^c^	OLS (10) ^d^	OLS (7) ^e^	GLM (8) ^e^
FOSQ 10				−0.017 (0.010)*	
(FOSQ 10 total score)^−2^	0.000 (0.000)***	0.000 (0.000)***			
(FOSQ 10 total score)^−0.5^	−1.564 (0.444)***	−0.812 (0.094)***			
OSA status	−0.177 (0.054)***	−0.043 (0.012)***	−0.035 (0.012)***		0.073 (0.044)*
Income level	0.238 (0.051)***	0.061 (0.011)***	0.060 (0.011)***		−0.118 (0.038)***
Education level	0.193 (0.053)***	0.036 (0.011)***	0.040 (0.011)***		
Gender			−0.056 (0.090)***		
Concentration (< = 0)			0.283 (0.055)***		
Concentration			0.056 (0.009)***		
(Concentration)^3^	0.005 (0.001)***	0.001 (0.000)***			
Relationship (< = 0)			0.182 (0.055)***		
Relationship			0.026 (0.011)**		
Morning activity (< = 0)			0.075 (0.058)		
Morning activity	0.135 (0.035)***	0.022 (0.008)***	0.034 (0.009)***		
Evening activity (< = 0)			−0.031 (0.057)		
Evening activity	0.175 (0.037)***	0.031 (0.008)***	0.035 (0.009)***		
Social (< = 0)			−0.064 (0.033)**		
(Social)^−2^			−0.317 (0.056)***		
Intimacy (< = 0)			0.020 (0.027)		
Intimacy	0.070 (0.017)***	0.010 (0.004)***	0.021 (0.007)**		
1. Concentration				−0.225 (0.072)***	0.859 (0.327)***
2. Concentration				−0.098 (0.059)*	0.194 (0.201)
3. Concentration				−0.069 (0.054)	0.144 (0.175)
4. Concentration				−0.006 (0.053)	0.053 (0.170)
1. Remembering				−0.067 (0.079)	0.616 (0.327)
2. Remembering				−0.081 (0.068)	0.111 (0.238)
3. Remembering				−0.055 (0.065)	0.076 (0.223)
4. Remembering				−0.037 (0.065)	0.057 (0.221)
1. Relationship				−0.170 (0.060)***	0.678 (0.323)**
2. Relationship				−0.110 (0.051)**	0.240 (0.205)
3. Relationship				−0.111 (0.045)***	0.260 (0.164)
4. Relationship				−0.077 (0.044)*	0.205 (0.157)
1. Morning activity				−0.014 (0.062)	0.128 (0.266)
2. Morning activity				−0.018 (0.056)	0.086 (0.228)
3. Morning activity				0.013 (0.054)	0.038 (0.216)
4. Morning activity				0.059 (0.053)	−0.047 (0.214)
1. Evening activity				0.067 (0.063)	−0.002 (0.306)
2. Evening activity				0.137 (0.055)**	−0.384 (0.243)
3. Evening activity				0.171 (0.051)***	−0.440 (0.229)*
4. Evening activity				0.209 (0.051)***	−0.504 (0.227)**
1. Driving short distance				−0.141 (0.057)***	0.764 (0.358)**
2. Driving short distance				−0.050 (0.045)**	0.121 (0.199)
3. Driving short distance				−0.011 (0.033)	0.017 (0.125)
4. Driving short distance				0.003 (0.028)	−0.003 (0.010)
1. Driving long distance				−0.026 (0.047)	0.097 (0.211)
2. Driving long distance				−0.032 (0.035)	0.061 (0.142)
3. Driving long distance				−0.003 (0.027)	0.024 (0.094)
4. Driving long distance				0.008 (0.024)	0.008 (0.083)
1. Watching movies				0.015 (0.061)	0.060 (0.265)
2. Watching movies				0.018 (0.052)	−0.016 (0.193)
3. Watching movies				0.024 (0.048)	−0.011 (0.173)
4. Watching movies				0.035 (0.048)	−0.017 (0.172)
1. Social				−0.255 (0.069)***	3.958 (0.853)***
2. Social				−0.023 (0.047)	0.013 (0.195)
3. Social				0.007 (0.037)	−0.041 (0.147)
4. Social				0.054 (0.036)	−0.091 (0.137)
1. Intimacy				−0.049 (0.035)	0.081 (0.115)
2. Intimacy				0.048 (0.027)*	−0.123 (0.092)
3. Intimacy				0.039 (0.018)**	−0.074 (0.065)
4. Intimacy				0.071 (0.015)***	−0.098 (0.053)*
Constant	0.717 (0.049)***	0.673 (0.011)***	0.719 (0.012)***	0.861 (0.172)***	1.658 (0.274)***
RMSE	0.19971	0.20169	0.20206	0.20411	0.20412
MAE	0.14331	0.13737	0.14732	0.14842	0.14868
R^2^	0.29094	0.31863	0.31866	0.31863	0.265566

*FOSQ-10 Functional Outcomes of Sleep Questionnaire*,* OSA Obstructive Sleep Apnoea*,* RMSE* root mean squared error, *MAE* mean absolute error, *R*^*2*^ proportion of the variation in the dependent variable predicted from the independent variables

* Statistically significant at 0.1 level, ** statistically significant at 0.05 level, *** statistically significant at 0.01 level

^a^ The whole sample includes the estimation and validation sample (*n* = 1,514)

^b^ Estimation method: *BB* beta-binomial, *OLS* ordinary least square, *GLM* generalised linear model

^c^ Multivariable fractional polynomial model selection, includes: FOSQ-10 total score^− 2^−0.33, (FOSQ 10 total score + 4.77^e7^)/10)^−0.5^−0.76, income ^f^, OSA ^g^, education ^h^, (concentration + 1)^3^−79.65, morning activity − 3.35, evening activity − 3.26, intimacy − 2.66

^d^ Multivariable fractional polynomial model selection with indicator for categorical variables, includes: income ^f^, OSA ^g^, education^h^, concentration − 3.35, relationship − 3.66, morning activity^3^−3.39, evening activity-3.31, social^− 2^−0.07, intimacy-3.30

^e^ FOSQ-10 domain scores entered as categorical variables scored as follows: 1 = Extreme difficulty/Extremely, 2 = Moderate difficulty/Moderately, 3 = Slight difficulty/A little, 4 = No difficulty/No and 5 = I don’t do this activity (reference)

^f^ OSA: dummy = 1 if diagnosed with OSA and 0 if undiagnosed

^g^ Income: dummy = 1 if earning more than AU 60,000/year

^h^ Education: dummy = 1 if attaining education higher than high-school/Year 12 level and 0 if not finished high-school/Year 12 level

Where Item 1 = (FOSQ 10 total score)^−2^−0.33, Item 2 = (FOSQ 10 total score + 4.77^e7^)/10)^−0.5^−0.76, Item 3 = Income level is a dummy variable equal to 1 if earning more than AU$ 60,000/year and 0 if lower than AU$ 60,000/year, Item 4 = Diagnosed with OSA is a dummy variable equal to 1 if diagnosed with OSA and 0 if undiagnosed, Item 5 = Education level is a dummy variable equal to 1 if attaining education higher than high-school and 0 if not finished high-school, Item 6 = (Concentration + 1)^3^−79.65, Item 7 = Morning Activity-3.35, Item 8 = Evening activity-3.26, Item 9 = Intimacy-2.66.

## Discussion

This is the first study to directly compare and map FOSQ-10 to EQ-5D-5 L in an adult population sample. The EQ-5D-5 L focuses more on the general health-related quality of life, while the FOSQ-10 concentrates on sleep-specific quality-of-life. We found that there were moderate levels of agreement between these instruments. Although there were statistically significant correlations between the two instruments, these correlations of the summary scores, utilities and subdimensions were moderate at best.

Despite the apparent low and moderate correlations found between individual dimensions of both instruments, correlations between the overall utilities of EQ-5D-5 L and FOSQ-10 summary scores were all moderate. Previous research deemed moderate levels of agreement for utilities and summary scores sufficient to determine that such instruments can be interchangeable [45, 46]. In economic evaluation, the measure of interest is the mean value of the summary scores or overall utilities, with low concern for the item responses [39, 43, 44]. Therefore, both instruments are considered applicable for measuring the quality-of-life outcomes in sleep disorders, particularly OSA. However, depending on the context in which the quality-of-life instruments need to be applied, one measure may be more beneficial. In a situation where health status in terms of utility is required to be investigated (cost-utility analysis), EQ-5D-5 L may be considered the ideal choice. In comparison, FOSQ-10 may be deemed more appropriate when a non-preference-based outcome is desired and to see a more drastic change in health status in a population with OSA.

The FOSQ-10 is a commonly used quality-of-life instrument for people with sleep disorders like OSA. However, as it is a non-preference based HRQoL measure, it has limited capacity when comparing interventions for diverse services and interventions, particularly in economic evaluation studies. This study aimed to investigate whether it was possible to overcome this limitation by developing a regression-based algorithm to predict EQ-5D-5 L utility scores from FOSQ-10. Based on an analysis of 1,514 respondents, our results demonstrate that it is possible to map FOSQ-10 onto EQ-5D-5 L utilities. The resulting predicted EQ-5D-5 L utilities from mappings may then be used to calculate QALYs for the conduct of CUA in studies concerned with sleep disorders. For this exercise, we recommend using BB (9), a beta regression model using significant variables denoted from multivariable fractional polynomial (i.e., the summary score of FOSQ-10, several of FOSQ-10 domains, and demographic criteria: OSA status, income, and education level).

Initially, the RMSE was used to identify the five best-fitting models in the validation sample (BB (10), BB (9), BB (11), BB (8) and BB (5)) and the estimation sample (BB (9), OLS (9), OLS (10), OLS (7) and GLM (8)). The magnitude of the primary measures of predictive ability (RMSE and MAE) for these high-ranked models in the validation (0.1997–0.2041 for RMSE and 0.1430–0.1458 for MAE) and the estimation (0.1898–0.1937 for RMSE and 0.1433–0.1496 for MAE) samples were all within the range of those reported in the literature [34]. Although BB (10) had the lowest RMSE in the validation sample, BB (9) proved to be consistent in its predictive ability in both estimation and validation samples. Consequently, BB (9) was chosen as the best-fitting model in both samples.

It is important to note that the predicted scores from BB (9) had relatively narrower ranges than those of observed utilities, as shown elsewhere [39, 47]. This occurrence may be caused due to the restriction imposed on the BB (9) that limits utilities from 0 to 1. However, BB (9) predicted EQ-5D-5 L utility scores whose group means were close to the observed utilities, with mean differences of 0.004 (estimation sample) and 0.001 (validation sample). The mean differences for both samples were lower than the minimum important differences of the EQ-5D-5 L reported in the other studies (range 0.051 [48] to 0.1 [49]). As mean estimates are the crucial measures within economic evaluations [39, 43, 44], our results suggested that BB (9) had acceptable predictive ability. While there has been a growing number of mapping studies in past years, comparing the results with other comparable analyses was not viable as our study is the first to develop a mapping algorithm for FOSQ-10 to EQ-5D-5 L.

This study has several limitations to be noted. First, mapping FOSQ-10 to EQ-5D-5 L introduces uncertainty, as the generic instrument may not fully capture the impact of sleep disturbances on health-related quality of life. Despite the adequate predictive ability of the models, they tended to underestimate the utility with excellent health and overestimate utility for those with poor health, as seen elsewhere [44]. However, the prediction accuracy, particularly for mean estimates, was similar to the observed utilities and was lower than the range of minimum important differences of the EQ-5D-5 L reported in the literature of 0.051 [48] to 0.1 [49]. While future research should consider validating the mapping algorithms developed in this study using an external sample, it is essential to note that the mapping algorithm has not been validated in an independent external dataset. However, the models performed well in a large cross-validated samples incorporated in this study. Second, a UK value set was used to estimate the EQ-5D-5 L utilities, as the Australian value set was unavailable at the time of analysis [27]. Several guidelines suggested the use of reference weights in accordance with the country or jurisdiction of interest as population health states’ values may vary across regions [8, 10]. However, previous research comparing EQ-5D instruments in Australia using value sets from the UK and the US found that although there are some observed variations between the population norms cross-countries, the UK value set provided relatively comparable valuations to that of Australia [50]. Third, additional uncertainty arose from using an indirect estimation based on the UK value set, which applied a crosswalk mapping to derive EQ-5D-3 L utilities from EQ-5D-5 L [27]. Future studies are encouraged to repeat the analysis using Australian values set to estimate the EQ-5D-5 L utilities to maximise local relevance and accuracy.

## Conclusions

The FOSQ-10 and EQ-5D-5 L are suitable for measuring the quality-of-life outcomes in a population with a diagnosed or high risk of sleep disorder such as OSA. The study findings support using both instruments as outcome measures in economic evaluations that provide different perspectives in their applications. We encourage using FOSQ-10 and EQ-5D-5 L simultaneously to capture broader aspects of quality of life and health status of interest within economic evaluations. In terms of utility score, collecting utility data using a generic preference-based instrument such as EQ-5D-5 L will always be the most recommended choice for measuring the quality of life to conduct an economic evaluation, particularly CUA. Alternatively, when direct data collection has not been done, the mapping equation provided in this study can be utilised to predict utilities if HRQoL of only FOSQ-10 data has been collected. BB (9) mapping equation is recommended for this exercise.

## Supplementary information

Below is the link to the electronic supplementary material.

ESM 1 (DOCX 241 KB)

## Data Availability

The datasets generated and analysed during the current study are not publicly available as they may be disclosive, which would contravene ethical approval. Appropriately anonymised data and relevant code are available from the corresponding author on reasonable request.
