# Supplementary material for: Predicting EuroQol (EQ-5D-5 L) health state utilities from functional outcomes of sleep questionnaire (FOSQ-10) scores
Source: Eur J Health Econ. 2026 Jan 31;27(7):1387–402. doi: 10.1007/s10198-025-01885-1 (PMC13619769; doi:10.1007/s10198-025-01885-1)
Supplement: Supplementary file 1 [file 10198_2025_1885_MOESM1_ESM.docx]

## Supplementary Materials

### Title

Predicting EuroQol (EQ-5D-5L) health state utilities from Functional Outcomes of Sleep Questionnaire (FOSQ-10) scores

### Authors

Andrea N. Natsky, Ching Li Chai-Coetzer, Andrew Vakulin, Billingsley Kaambwa

### Corresponding author

Andrea N. Natsky

University of Sydney, Australia

[andrea.natsky@sydney.edu.au](mailto:andrea.natsky@sydney.edu.au)

### Table of Contents

[**Table S1** Predictive ability of models across estimation and validation samples (k=10)	1](#_Toc157877625)

[**Table S2** Predictive ability of best fitting models when gender, area and age group dummies are included as explanatory variables	30](#_Toc157877626)

[**Table S3** Predicting utilities of EQ-5D-5L from FOSQ-10 using mapping algorithm BB 9	31](#_Toc157877627)

[**Table S4** Marginal effect for the highest rank model (BB 9) based on the whole sample (n=1,514)	32](#_Toc157877628)

**Table S1** Predictive ability of models across estimation and validation samples (k=10)

| Estimation method^a^ | | Mean (SD) | Min. | P.25 | Median | P.75 | Max. | Corr | R^2^ | RMSE | MAE | % <0.10 | % <0.25 |
| --- | --- | --- | --- | --- | --- | --- | --- | --- | --- | --- | --- | --- | --- |
|  | **Estimation sample (n = 1362)*** | | | | | | | | | | | | |
| Observed EQ-5D-5L | | 0.709 (0.247) | -0.594 | 0.635 | 0.761 | 0.846 | 1 |  |  |  |  |  |  |
| OLS (1) | |  |  |  |  |  |  |  |  |  |  |  |  |
| fold 1 | | 0.711 (0.120) | 0.184 | 0.658 | 0.753 | 0.803 | 0.817 | 0.503 | 0.253 | 0.20728 | 0.04296 | 46% | 84% |
| fold 2 | | 0.712 (0.122) | -0.015 | 0.659 | 0.756 | 0.805 | 0.819 | 0.509 | 0.259 | 0.20704 | 0.04287 | 47% | 84% |
| fold 3 | | 0.709 (0.118) | 0.017 | 0.653 | 0.746 | 0.799 | 0.812 | 0.483 | 0.234 | 0.23290 | 0.05424 | 46% | 83% |
| fold 4 | | 0.708 (0.120) | -0.006 | 0.656 | 0.745 | 0.800 | 0.814 | 0.495 | 0.245 | 0.21119 | 0.04460 | 46% | 83% |
| fold 5 | | 0.711 (0.122) | -0.013 | 0.658 | 0.748 | 0.803 | 0.817 | 0.504 | 0.254 | 0.20984 | 0.04403 | 46% | 83% |
| fold 6 | | 0.711 (0.122) | -0.027 | 0.660 | 0.748 | 0.804 | 0.818 | 0.499 | 0.249 | 0.21163 | 0.04479 | 46% | 83% |
| fold 7 | | 0.708 (0.119) | 0.013 | 0.655 | 0.748 | 0.802 | 0.815 | 0.487 | 0.237 | 0.21430 | 0.04592 | 46% | 83% |
| fold 8 | | 0.705 (0.123) | -0.003 | 0.646 | 0.748 | 0.803 | 0.816 | 0.498 | 0.248 | 0.21495 | 0.04620 | 45% | 83% |
| fold 9 | | 0.710 (0.119) | 0.003 | 0.659 | 0.747 | 0.801 | 0.815 | 0.495 | 0.245 | 0.20850 | 0.04347 | 47% | 84% |
| fold 10 | | 0.709 (0.124) | -0.015 | 0.650 | 0.747 | 0.803 | 0.816 | 0.499 | 0.249 | 0.21493 | 0.04619 | 45% | 83% |
| CV Average | | 0.709 (0.121) | 0.014 | 0.655 | 0.749 | 0.802 | 0.816 | 0.497 | 0.247 | 0.21326 | 0.15431 | 46% | 83% |
|  | |  |  |  |  |  |  |  |  |  |  |  |  |
| OLS (2) | | 0.711 (0.127) | 0.203 | 0.652 | 0.749 | 0.791 | 0.865 | 0.528 | 0.279 | 0.20371 | 0.15240 | 47% | 84% |
| fold 1 | | 0.712 (0.128) | -0.025 | 0.655 | 0.748 | 0.799 | 0.864 | 0.531 | 0.282 | 0.20390 | 0.15173 | 48% | 84% |
| fold 2 | | 0.709 (0.125) | 0.010 | 0.650 | 0.743 | 0.790 | 0.864 | 0.512 | 0.262 | 0.21004 | 0.15185 | 47% | 83% |
| fold 3 | | 0.708 (0.126) | -0.014 | 0.651 | 0.741 | 0.793 | 0.863 | 0.519 | 0.270 | 0.20786 | 0.15193 | 47% | 84% |
| fold 4 | | 0.711 (0.128) | -0.021 | 0.747 | 0.747 | 0.798 | 0.866 | 0.529 | 0.280 | 0.20637 | 0.15182 | 48% | 83% |
| fold 5 | | 0.711 (0.128) | -0.034 | 0.657 | 0.745 | 0.795 | 0.868 | 0.525 | 0.276 | 0.20794 | 0.15210 | 46% | 83% |
| fold 6 | | 0.708 (0.126) | -0.003 | 0.645 | 0.741 | 0.789 | 0.864 | 0.514 | 0.264 | 0.21062 | 0.15185 | 47% | 83% |
| fold 7 | | 0.705 (0.130) | -0.017 | 0.643 | 0.740 | 0.791 | 0.865 | 0.524 | 0.274 | 0.21133 | 0.15198 | 46% | 83% |
| fold 8 | | 0.710 (0.126) | -0.010 | 0.653 | 0.745 | 0.794 | 0.868 | 0.525 | 0.276 | 0.20445 | 0.15193 | 47% | 83% |
| fold 9 | | 0.709 (0.131) | -0.032 | 0.651 | 0.747 | 0.791 | 0.870 | 0.528 | 0.279 | 0.21075 | 0.15220 | 45% | 82% |
| fold 10 | | 0.711 (0.127) | 0.203 | 0.652 | 0.749 | 0.791 | 0.865 | 0.528 | 0.279 | 0.20371 | 0.15240 | 47% | 83% |
| CV Average | | 0.709 (0.128) | 0.006 | 0.660 | 0.744 | 0.793 | 0.866 | 0.524 | 0.274 | 0.20770 | 0.15198 | 47% | 83% |
|  | |  |  |  |  |  |  |  |  |  |  |  |  |
| Estimation method^a^ | | Mean (SD) | Min. | P.25 | Median | P.75 | Max. | Corr | R^2^ | RMSE | MAE | % <0.10 | % <0.25 |
| OLS (3) | |  |  |  |  |  |  |  |  |  |  |  |  |
| fold 1 | | 0.711 (0.121) | 0.174 | 0.655 | 0.752 | 0.803 | 0.823 | 0.506 | 0.256 | 0.20690 | 0.15422 | 48% | 84% |
| fold 2 | | 0.712 (0.123) | 0.015 | 0.655 | 0.753 | 0.805 | 0.827 | 0.514 | 0.264 | 0.20643 | 0.15355 | 48% | 84% |
| fold 3 | | 0.709 (0.120) | 0.055 | 0.651 | 0.747 | 0.798 | 0.822 | 0.490 | 0.240 | 0.21316 | 0.15374 | 47% | 83% |
| fold 4 | | 0.708 (0.121) | 0.025 | 0.652 | 0.745 | 0.797 | 0.822 | 0.499 | 0.250 | 0.21059 | 0.15408 | 47% | 83% |
| fold 5 | | 0.711 (0.124) | 0.022 | 0.659 | 0.751 | 0.806 | 0.826 | 0.510 | 0.260 | 0.20909 | 0.15373 | 47% | 83% |
| fold 6 | | 0.711 (0.123) | 0.006 | 0.652 | 0.751 | 0.801 | 0.827 | 0.504 | 0.254 | 0.21101 | 0.15391 | 47% | 83% |
| fold 7 | | 0.708 (0.120) | 0.041 | 0.653 | 0.749 | 0.796 | 0.822 | 0.491 | 0.241 | 0.21388 | 0.15358 | 47% | 83% |
| fold 8 | | 0.705 (0.124) | 0.025 | 0.644 | 0.744 | 0.798 | 0.824 | 0.502 | 0.252 | 0.21449 | 0.15366 | 47% | 83% |
| fold 9 | | 0.710 (0.120) | 0.036 | 0.654 | 0.746 | 0.799 | 0.825 | 0.502 | 0.252 | 0.20771 | 0.15373 | 48% | 83% |
| fold 10 | | 0.709 (0.125) | 0.015 | 0.649 | 0.751 | 0.800 | 0.825 | 0.503 | 0.253 | 0.21439 | 0.15386 | 47% | 83% |
| CV Average | | 0.709 (0.122) | 0.041 | 0.652 | 0.749 | 0.800 | 0.824 | 0.502 | 0.252 | 0.21077 | 0.15381 | 47% | 83% |
|  | |  |  |  |  |  |  |  |  |  |  |  |  |
| OLS (4) | |  |  |  |  |  |  |  |  |  |  |  |  |
| fold 1 | | 0.711 (0.126) | 0.213 | 0.656 | 0.749 | 0.784 | 0.856 | 0.525 | 0.275 | 0.20422 | 0.15331 | 47% | 83% |
| fold 2 | | 0.712 (0.126) | -0.057 | 0.656 | 0.748 | 0.791 | 0.854 | 0.525 | 0.276 | 0.20471 | 0.15272 | 48% | 84% |
| fold 3 | | 0.709 (0.123) | -0.029 | 0.652 | 0.744 | 0.781 | 0.852 | 0.505 | 0.255 | 0.21105 | 0.15285 | 46% | 83% |
| fold 4 | | 0.708 (0.125) | -0.048 | 0.653 | 0.743 | 0.785 | 0.852 | 0.514 | 0.264 | 0.20860 | 0.15290 | 47% | 83% |
| fold 5 | | 0.711 (0.127) | -0.058 | 0.658 | 0.746 | 0.789 | 0.856 | 0.522 | 0.273 | 0.20727 | 0.15279 | 47% | 83% |
| fold 6 | | 0.711 (0.127) | -0.069 | 0.658 | 0.744 | 0.787 | 0.857 | 0.520 | 0.270 | 0.20874 | 0.15308 | 46% | 83% |
| fold 7 | | 0.708 (0.125) | -0.034 | 0.651 | 0.741 | 0.782 | 0.855 | 0.509 | 0.259 | 0.21123 | 0.15282 | 46% | 83% |
| fold 8 | | 0.705 (0.129) | -0.047 | 0.644 | 0.741 | 0.783 | 0.855 | 0.519 | 0.269 | 0.21196 | 0.15295 | 45% | 83% |
| fold 9 | | 0.710 (0.124) | -0.046 | 0.655 | 0.742 | 0.783 | 0.855 | 0.517 | 0.267 | 0.20555 | 0.15290 | 47% | 84% |
| fold 10 | | 0.709 (0.130) | -0.065 | 0.655 | 0.747 | 0.782 | 0.860 | 0.523 | 0.273 | 0.21150 | 0.15324 | 46% | 83% |
| CV Average | | 0.709 (0.126) | -0.024 | 0.654 | 0.745 | 0.785 | 0.855 | 0.518 | 0.268 | 0.20848 | 0.15295 | 46% | 83% |
|  | |  |  |  |  |  |  |  |  |  |  |  |  |
|  | |  |  |  |  |  |  |  |  |  |  |  |  |
| Estimation method^a^ | | Mean (SD) | Min. | P.25 | Median | P.75 | Max. | Corr | R^2^ | RMSE | MAE | % <0.10 | % <0.25 |
| OLS (5) | |  |  |  |  |  |  |  |  |  |  |  |  |
| fold 1 | | 0.707 (0.132) | -0.101 | 0.652 | 0.748 | 0.789 | 0.863 | 0.527 | 0.279 | 0.20377 | 0.15236 | 46% | 83% |
| fold 2 | | 0.712 (0.128) | 0.085 | 0.649 | 0.747 | 0.804 | 0.869 | 0.532 | 0.283 | 0.20391 | 0.15191 | 47% | 84% |
| fold 3 | | 0.709 (0.125) | 0.156 | 0.640 | 0.741 | 0.797 | 0.871 | 0.514 | 0.264 | 0.20993 | 0.15216 | 45% | 83% |
| fold 4 | | 0.708 (0.127) | 0.163 | 0.642 | 0.741 | 0.802 | 0.871 | 0.521 | 0.272 | 0.20767 | 0.15229 | 46% | 83% |
| fold 5 | | 0.711 (0.129) | 0.079 | 0.648 | 0.746 | 0.802 | 0.871 | 0.530 | 0.280 | 0.20635 | 0.15198 | 46% | 83% |
| fold 6 | | 0.711 (0.128) | 0.022 | 0.654 | 0.744 | 0.797 | 0.870 | 0.526 | 0.276 | 0.20799 | 0.15217 | 46% | 83% |
| fold 7 | | 0.708 (0.126) | 0.155 | 0.636 | 0.739 | 0.796 | 0.872 | 0.516 | 0.266 | 0.21048 | 0.15214 | 45% | 83% |
| fold 8 | | 0.705 (0.130) | 0.080 | 0.637 | 0.739 | 0.795 | 0.870 | 0.524 | 0.275 | 0.21132 | 0.15216 | 45% | 83% |
| fold 9 | | 0.710 (0.126) | 0.102 | 0.646 | 0.742 | 0.799 | 0.873 | 0.526 | 0.277 | 0.20441 | 0.15214 | 46% | 84% |
| fold 10 | | 0.709 (0.131) | 0.094 | 0.642 | 0.744 | 0.796 | 0.876 | 0.529 | 0.280 | 0.21068 | 0.15248 | 45% | 83% |
| CV Average | | 0.710 (0.128) | 0.084 | 0.645 | 0.743 | 0.798 | 0.871 | 0.524 | 0.275 | 0.20765 | 0.15218 | 46% | 83% |
|  | |  |  |  |  |  |  |  |  |  |  |  |  |
| OLS (6) | |  |  |  |  |  |  |  |  |  |  |  |  |
| fold 1 | | 0.711 (0.127) | 0.198 | 0.653 | 0.749 | 0.791 | 0.864 | 0.528 | 0.279 | 0.20378 | 0.15449 | 47% | 84% |
| fold 2 | | 0.712 (0.128) | 0.052 | 0.649 | 0.747 | 0.804 | 0.869 | 0.531 | 0.282 | 0.20394 | 0.15188 | 47% | 84% |
| fold 3 | | 0.709 (0.125) | 0.119 | 0.640 | 0.740 | 0.797 | 0.871 | 0.514 | 0.264 | 0.20994 | 0.15216 | 45% | 83% |
| fold 4 | | 0.708 (0.127) | 0.108 | 0.642 | 0.741 | 0.801 | 0.871 | 0.521 | 0.271 | 0.20772 | 0.15225 | 46% | 83% |
| fold 5 | | 0.711 (0.129) | 0.044 | 0.649 | 0.746 | 0.802 | 0.871 | 0.529 | 0.280 | 0.20638 | 0.15194 | 46% | 83% |
| fold 6 | | 0.711 (0.128) | -0.004 | 0.655 | 0.744 | 0.797 | 0.870 | 0.526 | 0.276 | 0.20800 | 0.15214 | 46% | 83% |
| fold 7 | | 0.708 (0.126) | 0.105 | 0.638 | 0.738 | 0.796 | 0.872 | 0.515 | 0.265 | 0.21053 | 0.15211 | 45% | 83% |
| fold 8 | | 0.705 (0.130) | 0.050 | 0.637 | 0.739 | 0.795 | 0.870 | 0.524 | 0.275 | 0.21134 | 0.15213 | 45% | 83% |
| fold 9 | | 0.710 (0.126) | 0.070 | 0.647 | 0.742 | 0.799 | 0.873 | 0.526 | 0.276 | 0.20443 | 0.15212 | 47% | 84% |
| fold 10 | | 0.709 (0.131) | 0.058 | 0.643 | 0.743 | 0.797 | 0.876 | 0.529 | 0.280 | 0.21070 | 0.15246 | 45% | 83% |
| CV Average | | 0.710 (0.128) | 0.080 | 0.645 | 0.743 | 0.798 | 0.871 | 0.524 | 0.275 | 0.20768 | 0.15237 | 46% | 83% |
|  | |  |  |  |  |  |  |  |  |  |  |  |  |
| Estimation method^a^ | | Mean (SD) | Min. | P.25 | Median | P.75 | Max. | Corr | R^2^ | RMSE | MAE | % <0.10 | % <0.25 |
| OLS (7) | |  |  |  |  |  |  |  |  |  |  |  |  |
| fold 1 | | 0.711 (0.133) | -0.004 | 0.640 | 0.742 | 0.824 | 0.901 | 0.557 | 0.310 | 0.20221 | 0.14964 | 45% | 85% |
| fold 2 | | 0.712 (0.139) | -0.121 | 0.645 | 0.748 | 0.824 | 0.937 | 0.578 | 0.335 | 0.19912 | 0.14818 | 47% | 85% |
| fold 3 | | 0.709 (0.135) | -0.065 | 0.634 | 0.736 | 0.828 | 0.965 | 0.553 | 0.306 | 0.20662 | 0.14874 | 44% | 83% |
| fold 4 | | 0.708 (0.137) | -0.074 | 0.637 | 0.739 | 0.844 | 0.912 | 0.563 | 0.317 | 0.20383 | 0.14856 | 45% | 83% |
| fold 5 | | 0.711 (0.141) | -0.101 | 0.641 | 0.746 | 0.827 | 0.958 | 0.579 | 0.335 | 0.20112 | 0.14794 | 46% | 84% |
| fold 6 | | 0.711 (0.137) | -0.163 | 0.643 | 0.744 | 0.821 | 0.912 | 0.563 | 0.317 | 0.20483 | 0.14874 | 45% | 83% |
| fold 7 | | 0.708 (0.135) | -0.062 | 0.637 | 0.735 | 0.822 | 0.975 | 0.552 | 0.305 | 0.20766 | 0.14806 | 46% | 83% |
| fold 8 | | 0.705 (0.140) | -0.083 | 0.634 | 0.736 | 0.813 | 0.905 | 0.567 | 0.321 | 0.20730 | 0.14845 | 44% | 83% |
| fold 9 | | 0.710 (0.135) | -0.081 | 0.638 | 0.740 | 0.824 | 0.895 | 0.562 | 0.316 | 0.20150 | 0.14728 | 45% | 85% |
| fold 10 | | 0.709 (0.141) | -0.132 | 0.634 | 0.746 | 0.831 | 0.895 | 0.570 | 0.325 | 0.20691 | 0.14865 | 44% | 84% |
| CV Average | | 0.710 (0.137) | -0.088 | 0.638 | 0.741 | 0.826 | 0.925 | 0.564 | 0.319 | 0.20411 | 0.14842 | 45% | 84% |
|  | |  |  |  |  |  |  |  |  |  |  |  |  |
| OLS (8) | |  |  |  |  |  |  |  |  |  |  |  |  |
| fold 1 | | 0.711 (0.137) | 0.006 | 0.637 | 0.738 | 0.818 | 0.939 | 0.574 | 0.329 | 0.19942 | 0.14838 | 48% | 85% |
| fold 2 | | 0.712 (0.142) | -0.103 | 0.642 | 0.746 | 0.819 | 0.967 | 0.591 | 0.349 | 0.19701 | 0.14649 | 47% | 84% |
| fold 3 | | 0.709 (0.140) | -0.052 | 0.634 | 0.737 | 0.819 | 1.046 | 0.573 | 0.329 | 0.20335 | 0.14748 | 44% | 84% |
| fold 4 | | 0.708 (0.140) | -0.050 | 0.637 | 0.738 | 0.816 | 0.984 | 0.578 | 0.334 | 0.20140 | 0.14717 | 46% | 84% |
| fold 5 | | 0.711 (0.144) | -0.093 | 0.640 | 0.747 | 0.823 | 1.026 | 0.593 | 0.351 | 0.33060 | 0.14780 | 47% | 84% |
| fold 6 | | 0.711 (0.141) | -0.142 | 0.641 | 0.742 | 0.815 | 0.978 | 0.580 | 0.336 | 0.20208 | 0.14718 | 46% | 84% |
| fold 7 | | 0.708 (0.140) | -0.043 | 0.637 | 0.735 | 0.815 | 1.019 | 0.570 | 0.324 | 0.20479 | 0.14706 | 45% | 84% |
| fold 8 | | 0.705 (0.144) | -0.060 | 0.629 | 0.737 | 0.811 | 0.977 | 0.582 | 0.339 | 0.20472 | 0.14715 | 45% | 85% |
| fold 9 | | 0.710 (0.140) | -0.069 | 0.639 | 0.739 | 0.819 | 0.969 | 0.582 | 0.339 | 0.19824 | 0.14720 | 46% | 85% |
| fold 10 | | 0.709 (0.146) | -0.110 | 0.636 | 0.741 | 0.817 | 0.974 | 0.587 | 0.345 | 0.20384 | 0.14732 | 44% | 85% |
| CV Average | | 0.710 (0.141) | -0.072 | 0.637 | 0.740 | 0.817 | 0.988 | 0.581 | 0.337 | 0.21455 | 0.14732 | 46% | 85% |
|  | |  |  |  |  |  |  |  |  |  |  |  |  |
|  | |  |  |  |  |  |  |  |  |  |  |  |  |
|  | |  |  |  |  |  |  |  |  |  |  |  |  |
|  | |  |  |  |  |  |  |  |  |  |  |  |  |
| Estimation method^a^ | | Mean (SD) | Min. | P.25 | Median | P.75 | Max. | Corr | R^2^ | RMSE | MAE | % <0.10 | % <0.25 |
| OLS (9) | |  |  |  |  |  |  |  |  |  |  |  |  |
| fold 1 | | 0.711 (0.135) | -0.125 | 0.648 | 0.742 | 0.802 | 0.884 | 0.563 | 0.317 | 0.19870 | 0.14877 | 46% | 85% |
| fold 2 | | 0.712 (0.138) | -0.019 | 0.652 | 0.746 | 0.805 | 1.000 | 0.573 | 0.329 | 0.19763 | 0.14832 | 47% | 85% |
| fold 3 | | 0.709 (0.135) | 0.045 | 0.643 | 0.739 | 0.804 | 1.000 | 0.553 | 0.306 | 0.20421 | 0.14874 | 45% | 83% |
| fold 4 | | 0.708 (0.136) | 0.029 | 0.643 | 0.740 | 0.801 | 1.000 | 0.560 | 0.314 | 0.20195 | 0.14858 | 46% | 84% |
| fold 5 | | 0.711 (0.139) | 0.003 | 0.649 | 0.746 | 0.802 | 1.000 | 0.571 | 0.326 | 0.20006 | 0.14831 | 46% | 84% |
| fold 6 | | 0.711 (0.138) | -0.068 | 0.652 | 0.743 | 0.804 | 1.000 | 0.564 | 0.318 | 0.20225 | 0.14870 | 46% | 84% |
| fold 7 | | 0.708 (0.136) | 0.047 | 0.641 | 0.739 | 0.804 | 1.000 | 0.553 | 0.305 | 0.20509 | 0.14881 | 45% | 83% |
| fold 8 | | 0.705 (0.140) | 0.030 | 0.642 | 0.740 | 0.806 | 1.000 | 0.566 | 0.320 | 0.20497 | 0.14892 | 45% | 84% |
| fold 9 | | 0.710 (0.137) | 0.013 | 0.646 | 0.741 | 0.805 | 1.000 | 0.570 | 0.325 | 0.19788 | 0.14860 | 46% | 84% |
| fold 10 | | 0.709 (0.142) | 0.020 | 0.642 | 0.741 | 0.811 | 1.000 | 0.572 | 0.327 | 0.20411 | 0.14907 | 45% | 84% |
| CV Average | | 0.709 (0.137) | -0.002 | 0.646 | 0.742 | 0.804 | 0.988 | 0.564 | 0.319 | 0.20169 | 0.14868 | 46% | 84% |
|  | |  |  |  |  |  |  |  |  |  |  |  |  |
| OLS (10) | |  |  |  |  |  |  |  |  |  |  |  |  |
| fold 1 | | 0.711 (0.134) | 0.047 | 0.648 | 0.742 | 0.812 | 0.919 | 0.560 | 0.313 | 0.19969 | 0.14853 | 45% | 85% |
| fold 2 | | 0.712 (0.138) | -0.017 | 0.651 | 0.742 | 0.814 | 0.966 | 0.574 | 0.330 | 0.19785 | 0.14757 | 47% | 85% |
| fold 3 | | 0.709 (0.135) | 0.013 | 0.643 | 0.737 | 0.810 | 0.952 | 0.554 | 0.307 | 0.20437 | 0.14825 | 45% | 84% |
| fold 4 | | 0.708 (0.136) | -0.012 | 0.644 | 0.740 | 0.805 | 0.941 | 0.561 | 0.315 | 0.20216 | 0.14785 | 46% | 84% |
| fold 5 | | 0.711 (0.139) | -0.037 | 0.649 | 0.744 | 0.811 | 0.947 | 0.573 | 0.329 | 0.20003 | 0.13737 | 46% | 85% |
| fold 6 | | 0.711 (0.137) | -0.049 | 0.651 | 0.742 | 0.809 | 0.952 | 0.563 | 0.317 | 0.20284 | 0.14797 | 46% | 84% |
| fold 7 | | 0.708 (0.136) | 0.046 | 0.646 | 0.741 | 0.809 | 0.963 | 0.556 | 0.309 | 0.20491 | 0.14813 | 45% | 84% |
| fold 8 | | 0.705 (0.140) | -0.013 | 0.641 | 0.738 | 0.810 | 0.923 | 0.564 | 0.318 | 0.20568 | 0.14801 | 45% | 84% |
| fold 9 | | 0.710 (0.136) | -0.005 | 0.649 | 0.739 | 0.811 | 0.923 | 0.567 | 0.321 | 0.19871 | 0.14794 | 46% | 85% |
| fold 10 | | 0.709 (0.142) | -0.025 | 0.642 | 0.737 | 0.814 | 0.968 | 0.573 | 0.328 | 0.20433 | 0.14846 | 45% | 84% |
| CV Average | | 0.709 (0.137) | -0.005 | 0.646 | 0.740 | 0.811 | 0.945 | 0.564 | 0.319 | 0.20206 | 0.14701 | 46% | 84% |
|  | |  |  |  |  |  |  |  |  |  |  |  |  |
|  | |  |  |  |  |  |  |  |  |  |  |  |  |
|  | |  |  |  |  |  |  |  |  |  |  |  |  |
|  | |  |  |  |  |  |  |  |  |  |  |  |  |
| Estimation method^a^ | | Mean (SD) | Min. | P.25 | Median | P.75 | Max. | Corr | R^2^ | RMSE | MAE | % <0.10 | % <0.25 |
| OLS (11) | |  |  |  |  |  |  |  |  |  |  |  |  |
| fold 1 | | 0.711 (0.132) | 0.110 | 0.635 | 0.738 | 0.814 | 0.883 | 0.550 | 0.303 | 0.20113 | 0.15102 | 44% | 85% |
| fold 2 | | 0.689 (0.122) | 0.153 | 0.613 | 0.716 | 0.779 | 0.863 | 0.501 | 0.251 | 0.21278 | 0.14889 | 45% | 85% |
| fold 3 | | 0.712 (0.133) | 0.104 | 0.639 | 0.743 | 0.818 | 0.881 | 0.553 | 0.305 | 0.20132 | 0.15032 | 39% | 83% |
| fold 4 | | 0.708 (0.131) | 0.143 | 0.631 | 0.734 | 0.813 | 0.885 | 0.541 | 0.293 | 0.20529 | 0.15031 | 45% | 84% |
| fold 5 | | 0.711 (0.134) | 0.106 | 0.635 | 0.739 | 0.819 | 0.890 | 0.553 | 0.306 | 0.20329 | 0.15012 | 43% | 84% |
| fold 6 | | 0.711 (0.134) | 0.058 | 0.639 | 0.738 | 0.810 | 0.886 | 0.549 | 0.301 | 0.20509 | 0.15073 | 44% | 84% |
| fold 7 | | 0.708 (0.131) | 0.156 | 0.628 | 0.735 | 0.812 | 0.889 | 0.536 | 0.287 | 0.20810 | 0.15052 | 44% | 83% |
| fold 8 | | 0.705 (0.136) | 0.121 | 0.628 | 0.734 | 0.810 | 0.886 | 0.548 | 0.300 | 0.20831 | 0.15073 | 44% | 84% |
| fold 9 | | 0.710 (0.132) | 0.145 | 0.631 | 0.735 | 0.814 | 0.892 | 0.548 | 0.301 | 0.20167 | 0.15063 | 45% | 85% |
| fold 10 | | 0.709 (0.137) | 0.103 | 0.631 | 0.739 | 0.813 | 0.896 | 0.552 | 0.305 | 0.20768 | 0.15109 | 43% | 84% |
| CV Average | | 0.707 (0.132) | 0.120 | 0.631 | 0.735 | 0.810 | 0.885 | 0.543 | 0.295 | 0.20547 | 0.15044 | 44% | 84% |
|  | |  |  |  |  |  |  |  |  |  |  |  |  |
| CLAD (1) | |  |  |  |  |  |  |  |  |  |  |  |  |
| fold 1 | | 0.740 (0.111) | 0.256 | 0.692 | 0.779 | 0.824 | 0.837 | 0.503 | 0.123 | 0.21672 | 0.15211 | 48% | 84% |
| fold 2 | | 0.738 (0.062) | 0.062 | 0.689 | 0.779 | 0.824 | 0.837 | 0.509 | 0.124 | 0.21673 | 0.15211 | 48% | 85% |
| fold 3 | | 0.734 (0.117) | 0.046 | 0.679 | 0.771 | 0.824 | 0.837 | 0.483 | 0.112 | 0.21639 | 0.15216 | 47% | 83% |
| fold 4 | | 0.734 (0.110) | 0.080 | 0.687 | 0.769 | 0.819 | 0.831 | 0.495 | 0.113 | 0.21657 | 0.15240 | 47% | 84% |
| fold 5 | | 0.736 (0.116) | 0.053 | 0.687 | 0.772 | 0.824 | 0.837 | 0.504 | 0.113 | 0.21652 | 0.15214 | 47% | 83% |
| fold 6 | | 0.737 (0.113) | 0.053 | 0.690 | 0.772 | 0.824 | 0.837 | 0.499 | 0.110 | 0.21652 | 0.15214 | 47% | 83% |
| fold 7 | | 0.734 (0.115) | 0.066 | 0.683 | 0.773 | 0.824 | 0.837 | 0.487 | 0.113 | 0.21680 | 0.15210 | 46% | 83% |
| fold 8 | | 0.741 (0.107) | 0.129 | 0.689 | 0.778 | 0.825 | 0.837 | 0.498 | 0.117 | 0.21861 | 0.15230 | 47% | 83% |
| fold 9 | | 0.737 (0.113) | 0.062 | 0.689 | 0.772 | 0.824 | 0.837 | 0.495 | 0.115 | 0.21673 | 0.15211 | 47% | 84% |
| fold 10 | | 0.736 (0.117) | 0.053 | 0.680 | 0.772 | 0.824 | 0.837 | 0.499 | 0.119 | 0.21652 | 0.15214 | 46% | 84% |
| CV Average | | 0.737 (0.108) | 0.086 | 0.686 | 0.774 | 0.824 | 0.836 | 0.497 | 0.116 | 0.21681 | 0.15217 | 47% | 84% |
|  | |  |  |  |  |  |  |  |  |  |  |  |  |
|  | |  |  |  |  |  |  |  |  |  |  |  |  |
|  | |  |  |  |  |  |  |  |  |  |  |  |  |
|  | |  |  |  |  |  |  |  |  |  |  |  |  |
| Estimation method^a^ | | Mean (SD) | Min. | P.25 | Median | P.75 | Max. | Corr | R^2^ | RMSE | MAE | % <0.10 | % <0.25 |
| CLAD (2) | |  |  |  |  |  |  |  |  |  |  |  |  |
| fold 1 | | 0.743 (0.119) | 0.239 | 0.690 | 0.781 | 0.827 | 0.876 | 0.523 | 0.134 | 0.21361 | 0.15026 | 46% | 85% |
| fold 2 | | 0.737 (0.117) | 0.094 | 0.682 | 0.775 | 0.825 | 0.865 | 0.524 | 0.133 | 0.21407 | 0.15035 | 49% | 84% |
| fold 3 | | 0.738 (0.106) | 0.164 | 0.685 | 0.774 | 0.818 | 0.851 | 0.502 | 0.125 | 0.21548 | 0.15039 | 49% | 84% |
| fold 4 | | 0.735 (0.106) | 0.140 | 0.685 | 0.769 | 0.810 | 0.854 | 0.515 | 0.126 | 0.21425 | 0.15022 | 48% | 85% |
| fold 5 | | 0.746 (0.098) | 0.214 | 0.703 | 0.769 | 0.819 | 0.862 | 0.527 | 0.126 | 0.21650 | 0.15052 | 49% | 84% |
| fold 6 | | 0.095 (0.681) | 0.095 | 0.681 | 0.763 | 0.811 | 0.860 | 0.524 | 0.119 | 0.21237 | 0.15021 | 49% | 83% |
| fold 7 | | 0.741 (0.089) | 0.270 | 0.696 | 0.765 | 0.805 | 0.846 | 0.506 | 0.122 | 0.21803 | 0.15107 | 49% | 84% |
| fold 8 | | 0.748 (0.111) | 0.123 | 0.694 | 0.779 | 0.823 | 0.884 | 0.524 | 0.129 | 0.21586 | 0.15107 | 45% | 83% |
| fold 9 | | 0.730 (0.111) | 0.092 | 0.680 | 0.764 | 0.812 | 0.862 | 0.524 | 0.128 | 0.21251 | 0.15019 | 49% | 85% |
| fold 10 | | 0.736 (0.118) | 0.074 | 0.683 | 0.773 | 0.820 | 0.874 | 0.527 | 0.131 | 0.21278 | 0.15015 | 47% | 84% |
| CV Average | | 0.738 (0.108) | 0.151 | 0.688 | 0.771 | 0.817 | 0.864 | 0.519 | 0.127 | 0.21455 | 0.15044 | 48% | 84% |
|  | |  |  |  |  |  |  |  |  |  |  |  |  |
| CLAD (3) | |  |  |  |  |  |  |  |  |  |  |  |  |
| fold 1 | | 0.742 (0.099) | 0.306 | 0.697 | 0.773 | 0.821 | 0.837 | 0.503 | 0.129 | 0.21757 | 0.15130 | 49% | 84% |
| fold 2 | | 0.747 (0.094) | 0.237 | 0.703 | 0.777 | 0.820 | 0.837 | 0.512 | 0.125 | 0.21946 | 0.15174 | 50% | 85% |
| fold 3 | | 0.735 (0.104) | 0.213 | 0.685 | 0.768 | 0.818 | 0.837 | 0.485 | 0.120 | 0.21765 | 0.15166 | 48% | 84% |
| fold 4 | | 0.741 (0.102) | 0.186 | 0.697 | 0.773 | 0.818 | 0.839 | 0.499 | 0.119 | 0.21772 | 0.15135 | 48% | 84% |
| fold 5 | | 0.739 (0.103) | 0.192 | 0.694 | 0.773 | 0.821 | 0.837 | 0.508 | 0.120 | 0.21737 | 0.15132 | 49% | 84% |
| fold 6 | | 0.738 (0.102) | 0.184 | 0.690 | 0.772 | 0.817 | 0.837 | 0.502 | 0.115 | 0.21714 | 0.15131 | 49% | 84% |
| fold 7 | | 0.736 (0.106) | 0.147 | 0.688 | 0.773 | 0.814 | 0.837 | 0.491 | 0.117 | 0.21705 | 0.15138 | 47% | 84% |
| fold 8 | | 0.736 (0.104) | 0.184 | 0.685 | 0.770 | 0.815 | 0.837 | 0.500 | 0.122 | 0.21739 | 0.15128 | 48% | 84% |
| fold 9 | | 0.735 (0.104) | 0.184 | 0.685 | 0.766 | 0.815 | 0.837 | 0.499 | 0.122 | 0.21694 | 0.15142 | 49% | 84% |
| fold 10 | | 0.739 (0.111) | 0.096 | 0.689 | 0.775 | 0.825 | 0.837 | 0.501 | 0.125 | 0.21698 | 0.15188 | 47% | 84% |
| CV Average | | 0.739 (0.103) | 0.193 | 0.691 | 0.772 | 0.818 | 0.837 | 0.500 | 0.121 | 0.21753 | 0.15146 | 48% | 84% |
|  | |  |  |  |  |  |  |  |  |  |  |  |  |
|  | |  |  |  |  |  |  |  |  |  |  |  |  |
|  | |  |  |  |  |  |  |  |  |  |  |  |  |
|  | |  |  |  |  |  |  |  |  |  |  |  |  |
| Estimation method^a^ | | Mean (SD) | Min. | P.25 | Median | P.75 | Max. | Corr | R^2^ | RMSE | MAE | % <0.10 | % <0.25 |
| CLAD (4) | | 0.737 (0.114) | 0.237 | 0.687 | 0.777 | 0.824 | 0.837 | 0.503 | 0.128 | 0.21621 | 0.15222 | 47% | 84% |
| fold 1 | | 0.731 (0.112) | 0.047 | 0.682 | 0.764 | 0.809 | 0.850 | 0.524 | 0.128 | 0.21336 | 0.15120 | 48% | 85% |
| fold 2 | | 0.743 (0.114) | 0.065 | 0.691 | 0.779 | 0.832 | 0.856 | 0.496 | 0.116 | 0.21607 | 0.15143 | 47% | 84% |
| fold 3 | | 0.732 (0.112) | 0.053 | 0.683 | 0.765 | 0.804 | 0.859 | 0.514 | 0.118 | 0.21333 | 0.15120 | 47% | 84% |
| fold 4 | | 0.730 (0.099) | 0.129 | 0.690 | 0.758 | 0.789 | 0.846 | 0.522 | 0.119 | 0.21433 | 0.15148 | 49% | 83% |
| fold 5 | | 0.743 (0.110) | 0.066 | 0.695 | 0.780 | 0.831 | 0.851 | 0.510 | 0.114 | 0.21589 | 0.15143 | 48% | 83% |
| fold 6 | | 0.732 (0.109) | 0.085 | 0.680 | 0.765 | 0.810 | 0.849 | 0.506 | 0.118 | 0.21439 | 0.15111 | 47% | 84% |
| fold 7 | | 0.730 (0.109) | 0.081 | 0.680 | 0.764 | 0.806 | 0.849 | 0.517 | 0.124 | 0.21399 | 0.15110 | 47% | 84% |
| fold 8 | | 0.750 (0.060) | 0.060 | 0.702 | 0.786 | 0.832 | 0.869 | 0.513 | 0.121 | 0.21639 | 0.15154 | 48% | 85% |
| fold 9 | | 0.739 (0.102) | 0.126 | 0.694 | 0.772 | 0.807 | 0.848 | 0.520 | 0.125 | 0.15131 | 0.21541 | 48% | 84% |
| fold 10 | | 0.737 (0.114) | 0.237 | 0.687 | 0.777 | 0.824 | 0.837 | 0.503 | 0.128 | 0.21621 | 0.15781 | 47% | 84% |
| CV Average | | 0.737 (0.109) | 0.095 | 0.688 | 0.771 | 0.815 | 0.851 | 0.512 | 0.121 | 0.20853 | 0.15222 | 47% | 84% |
|  | |  |  |  |  |  |  |  |  |  |  |  |  |
| CLAD (5) | |  |  |  |  |  |  |  |  |  |  |  |  |
| fold 1 | | 0.746 (0.113) | 0.226 | 0.699 | 0.779 | 0.827 | 0.870 | 0.516 | 0.134 | 0.21527 | 0.15041 | 47% | 85% |
| fold 2 | | 0.740 (0.116) | -0.074 | 0.695 | 0.777 | 0.818 | 0.864 | 0.526 | 0.133 | 0.21390 | 0.15040 | 49% | 85% |
| fold 3 | | 0.727 (0.113) | 0.004 | 0.680 | 0.761 | 0.811 | 0.847 | 0.503 | 0.125 | 0.21352 | 0.15037 | 49% | 84% |
| fold 4 | | 0.748 (0.110) | 0.213 | 0.694 | 0.778 | 0.836 | 0.883 | 0.514 | 0.126 | 0.21613 | 0.15052 | 47% | 84% |
| fold 5 | | 0.741 (0.100) | 0.329 | 0.689 | 0.768 | 0.814 | 0.869 | 0.529 | 0.126 | 0.21492 | 0.15046 | 47% | 84% |
| fold 6 | | 0.732 (0.131) | -0.349 | 0.695 | 0.775 | 0.820 | 0.861 | 0.514 | 0.119 | 0.21401 | 0.15105 | 48% | 84% |
| fold 7 | | 0.742 (0.107) | -0.035 | 0.703 | 0.778 | 0.816 | 0.846 | 0.497 | 0.122 | 0.21677 | 0.15081 | 48% | 84% |
| fold 8 | | 0.727 (0.142) | -0.359 | 0.680 | 0.772 | 0.816 | 0.877 | 0.515 | 0.129 | 0.21355 | 0.15128 | 46% | 83% |
| fold 9 | | 0.735 (0.119) | 0.254 | 0.671 | 0.769 | 0.830 | 0.882 | 0.522 | 0.128 | 0.21289 | 0.15033 | 48% | 84% |
| fold 10 | | 0.735 (0.133) | -0.390 | 0.699 | 0.781 | 0.822 | 0.859 | 0.507 | 0.131 | 0.21538 | 0.15112 | 47% | 84% |
| CV Average | | 0.737 (0.118) | -0.018 | 0.690 | 0.774 | 0.821 | 0.866 | 0.514 | 0.127 | 0.21463 | 0.15068 | 47% | 84% |
|  | |  |  |  |  |  |  |  |  |  |  |  |  |
|  | |  |  |  |  |  |  |  |  |  |  |  |  |
|  | |  |  |  |  |  |  |  |  |  |  |  |  |
|  | |  |  |  |  |  |  |  |  |  |  |  |  |
| Estimation method^a^ | | Mean (SD) | Min. | P.25 | Median | P.75 | Max. | Corr | R^2^ | RMSE | MAE | % <0.10 | % <0.25 |
| CLAD (6) | |  |  |  |  |  |  |  |  |  |  |  |  |
| fold 1 | | 0.739 (0.115) | 0.338 | 0.675 | 0.772 | 0.831 | 0.883 | 0.524 | 0.134 | 0.21283 | 0.15021 | 48% | 84% |
| fold 2 | | 0.727 (0.131) | -0.269 | 0.689 | 0.766 | 0.812 | 0.853 | 0.519 | 0.133 | 0.21359 | 0.15113 | 49% | 85% |
| fold 3 | | 0.740 (0.101) | 0.277 | 0.685 | 0.773 | 0.816 | 0.859 | 0.507 | 0.125 | 0.21576 | 0.15037 | 48% | 84% |
| fold 4 | | 0.740 (0.113) | 0.113 | 0.691 | 0.773 | 0.826 | 0.866 | 0.510 | 0.126 | 0.21506 | 0.15029 | 48% | 84% |
| fold 5 | | 0.736 (0.109) | 0.400 | 0.669 | 0.767 | 0.821 | 0.878 | 0.518 | 0.126 | 0.21474 | 0.15054 | 47% | 83% |
| fold 6 | | 0.735 (0.102) | 0.000 | 0.703 | 0.762 | 0.798 | 0.848 | 0.521 | 0.119 | 0.21441 | 0.15049 | 50% | 83% |
| fold 7 | | 0.727 (0.113) | -0.044 | 0.685 | 0.763 | 0.808 | 0.844 | 0.496 | 0.122 | 0.21458 | 0.15070 | 49% | 83% |
| fold 8 | | 0.726 (0.139) | -0.232 | 0.678 | 0.769 | 0.817 | 0.870 | 0.516 | 0.129 | 0.21310 | 0.15110 | 46% | 83% |
| fold 9 | | 0.739 (0.103) | 0.306 | 0.683 | 0.761 | 0.818 | 0.869 | 0.517 | 0.128 | 0.21541 | 0.15031 | 48% | 85% |
| fold 10 | | 0.744 (0.112) | 0.121 | 0.694 | 0.773 | 0.826 | 0.866 | 0.515 | 0.131 | 0.21594 | 0.15040 | 48% | 84% |
| CV Average | | 0.735 (0.114) | 0.101 | 0.685 | 0.768 | 0.817 | 0.864 | 0.514 | 0.127 | 0.21454 | 0.15055 | 48% | 84% |
|  | |  |  |  |  |  |  |  |  |  |  |  |  |
| CLAD (7) | |  |  |  |  |  |  |  |  |  |  |  |  |
| fold 1 | | 0.731 (0.119) | 0.260 | 0.672 | 0.765 | 0.828 | 0.890 | 0.520 | 0.319 | 0.22876 | 0.16810 | 46% | 84% |
| fold 2 | | 0.733 (0.121) | 0.090 | 0.681 | 0.769 | 0.825 | 0.847 | 0.508 | 0.422 | 0.22402 | 0.13151 | 48% | 84% |
| fold 3 | | 0.737 (0.103) | 0.199 | 0.682 | 0.762 | 0.822 | 0.984 | 0.493 | 0.225 | 0.20420 | 0.15995 | 46% | 83% |
| fold 4 | | 0.742 (0.100) | 0.204 | 0.695 | 0.772 | 0.825 | 0.867 | 0.501 | 0.156 | 0.21722 | 0.14119 | 46% | 84% |
| fold 5 | | 0.737 (0.111) | 0.114 | 0.689 | 0.771 | 0.823 | 0.919 | 0.513 | 0.156 | 0.22575 | 0.16134 | 47% | 84% |
| fold 6 | | 0.734 (0.104) | 0.167 | 0.683 | 0.764 | 0.815 | 0.878 | 0.510 | 0.265 | 0.20556 | 0.14262 | 46% | 83% |
| fold 7 | | 0.730 (0.110) | 0.126 | 0.679 | 0.763 | 0.815 | 0.879 | 0.502 | 0.410 | 0.16573 | 0.14735 | 47% | 83% |
| fold 8 | | 0.734 (0.101) | 0.195 | 0.681 | 0.761 | 0.813 | 0.864 | 0.521 | 0.359 | 0.23395 | 0.16627 | 47% | 83% |
| fold 9 | | 0.728 (0.118) | 0.060 | 0.672 | 0.768 | 0.824 | 0.885 | 0.507 | 0.193 | 0.20099 | 0.13934 | 46% | 84% |
| fold 10 | | 0.737 (0.102) | 0.209 | 0.686 | 0.769 | 0.817 | 0.860 | 0.518 | 0.106 | 0.21966 | 0.15142 | 46% | 84% |
| CV Average | | 0.734 (0.109) | 0.162 | 0.682 | 0.766 | 0.821 | 0.887 | 0.509 | 0.261 | 0.21259 | 0.15091 | 47% | 84% |
|  | |  |  |  |  |  |  |  |  |  |  |  |  |
|  | |  |  |  |  |  |  |  |  |  |  |  |  |
|  | |  |  |  |  |  |  |  |  |  |  |  |  |
|  | |  |  |  |  |  |  |  |  |  |  |  |  |
| Estimation method^a^ | | Mean (SD) | Min. | P.25 | Median | P.75 | Max. | Corr | R^2^ | RMSE | MAE | % <0.10 | % <0.25 |
| CLAD (8) | |  |  |  |  |  |  |  |  |  |  |  |  |
| fold 1 | | 0.740 (0.366) | 0.366 | 0.692 | 0.771 | 0.817 | 0.860 | 0.524 | 0.166 | 0.22277 | 0.13930 | 47% | 84% |
| fold 2 | | 0.745 (0.112) | 0.248 | 0.685 | 0.771 | 0.835 | 0.906 | 0.520 | 0.208 | 0.19984 | 0.14100 | 46% | 84% |
| fold 3 | | 0.739 (0.110) | 0.251 | 0.679 | 0.768 | 0.821 | 0.885 | 0.502 | 0.268 | 0.21794 | 0.15369 | 46% | 84% |
| fold 4 | | 0.730 (0.118) | 0.219 | 0.666 | 0.759 | 0.826 | 0.900 | 0.517 | 0.288 | 0.21911 | 0.14490 | 45% | 84% |
| fold 5 | | 0.721 (0.111) | 0.210 | 0.666 | 0.753 | 0.803 | 0.851 | 0.505 | 0.289 | 0.20434 | 0.18504 | 49% | 83% |
| fold 6 | | 0.736 (0.105) | 0.250 | 0.682 | 0.763 | 0.811 | 0.869 | 0.510 | 0.310 | 0.21945 | 0.16835 | 48% | 84% |
| fold 7 | | 0.736 (0.097) | 0.320 | 0.681 | 0.758 | 0.812 | 0.931 | 0.509 | 0.233 | 0.21095 | 0.15273 | 47% | 83% |
| fold 8 | | 0.730 (0.127) | 0.120 | 0.665 | 0.760 | 0.827 | 0.891 | 0.514 | 0.348 | 0.21190 | 0.13937 | 46% | 83% |
| fold 9 | | 0.733 (0.111) | 0.198 | 0.680 | 0.764 | 0.820 | 0.867 | 0.504 | 0.174 | 0.21203 | 0.13806 | 47% | 85% |
| fold 10 | | 0.733 (0.119) | 0.177 | 0.672 | 0.765 | 0.825 | 0.895 | 0.519 | 0.235 | 0.21571 | 0.15449 | 46% | 83% |
| CV Average | | 0.734 (0.138) | 0.236 | 0.677 | 0.763 | 0.820 | 0.886 | 0.512 | 0.252 | 0.21340 | 0.15169 | 47% | 84% |
|  | |  |  |  |  |  |  |  |  |  |  |  |  |
| CLAD (9) | |  |  |  |  |  |  |  |  |  |  |  |  |
| fold 1 | | 0.743 (0.111) | 0.365 | 0.675 | 0.763 | 0.827 | 0.901 | 0.518 | 0.285 | 0.23157 | 0.14384 | 49% | 85% |
| fold 2 | | 0.744 (0.111) | 0.386 | 0.679 | 0.769 | 0.822 | 1.630 | 0.324 | 0.254 | 0.19122 | 0.15562 | 49% | 86% |
| fold 3 | | 0.730 (0.113) | 0.201 | 0.692 | 0.767 | 0.820 | 1.360 | 0.323 | 0.234 | 0.20158 | 0.15512 | 49% | 84% |
| fold 4 | | 0.734 (0.133) | 0.230 | 0.674 | 0.761 | 0.818 | 0.901 | 0.326 | 0.389 | 0.26187 | 0.15650 | 48% | 84% |
| fold 5 | | 0.734 (0.113) | 0.131 | 0.684 | 0.763 | 0.810 | 0.872 | 0.560 | 0.317 | 0.19043 | 0.14258 | 49% | 84% |
| fold 6 | | 0.739 (0.105) | 0.147 | 0.689 | 0.764 | 0.815 | 1.000 | 0.552 | 0.236 | 0.21053 | 0.12739 | 49% | 84% |
| fold 7 | | 0.739 (0.117) | 0.081 | 0.688 | 0.768 | 0.818 | 1.000 | 0.547 | 0.277 | 0.18242 | 0.14803 | 48% | 84% |
| fold 8 | | 0.738 (0.119) | 0.144 | 0.681 | 0.768 | 0.822 | 1.000 | 0.558 | 0.157 | 0.21930 | 0.13581 | 46% | 84% |
| fold 9 | | 0.741 (0.115) | 0.211 | 0.683 | 0.765 | 0.824 | 1.083 | 0.563 | 0.254 | 0.22855 | 0.14732 | 43% | 84% |
| fold 10 | | 0.731 (0.125) | 0.066 | 0.675 | 0.758 | 0.820 | 1.000 | 0.566 | 0.141 | 0.19726 | 0.18184 | 47% | 84% |
| CV Average | | 0.740 (0.116) | 0.196 | 0.682 | 0.765 | 0.820 | 1.075 | 0.484 | 0.254 | 0.21147 | 0.14941 | 48% | 84% |
|  | |  |  |  |  |  |  |  |  |  |  |  |  |
|  | |  |  |  |  |  |  |  |  |  |  |  |  |
| Estimation method^a^ | | Mean (SD) | Min. | P.25 | Median | P.75 | Max. | Corr | R^2^ | RMSE | MAE | % <0.10 | % <0.25 |
| CLAD (10) | |  |  |  |  |  |  |  |  |  |  |  |  |
| fold 1 | | 0.735 (0.109) | 0.311 | 0.684 | 0.766 | 0.813 | 0.988 | 0.548 | 0.273 | 0.21974 | 0.13237 | 49% | 86% |
| fold 2 | | 0.733 (0.125) | -0.023 | 0.684 | 0.759 | 0.811 | 0.973 | 0.564 | 0.371 | 0.19896 | 0.13239 | 49% | 85% |
| fold 3 | | 0.739 (0.117) | 0.083 | 0.689 | 0.771 | 0.820 | 0.918 | 0.550 | 0.489 | 0.22983 | 0.14408 | 49% | 85% |
| fold 4 | | 0.733 (0.130) | 0.028 | 0.671 | 0.763 | 0.828 | 1.000 | 0.561 | 0.338 | 0.18444 | 0.14987 | 47% | 85% |
| fold 5 | | 0.732 (0.129) | 0.050 | 0.678 | 0.768 | 0.827 | 1.009 | 0.564 | 0.254 | 0.19082 | 0.19063 | 49% | 85% |
| fold 6 | | 0.743 (0.127) | 0.098 | 0.693 | 0.781 | 0.829 | 1.060 | 0.537 | 0.314 | 0.21378 | 0.15443 | 47% | 84% |
| fold 7 | | 0.732 (0.136) | -0.017 | 0.676 | 0.767 | 0.837 | 0.937 | 0.551 | 0.204 | 0.19583 | 0.13433 | 48% | 84% |
| fold 8 | | 0.733 (0.141) | -0.060 | 0.672 | 0.767 | 0.826 | 1.095 | 0.558 | 0.185 | 0.21301 | 0.12851 | 46% | 84% |
| fold 9 | | 0.729 (0.112) | 0.275 | 0.672 | 0.759 | 0.814 | 0.961 | 0.552 | 0.154 | 0.22789 | 0.15338 | 48% | 85% |
| fold 10 | | 0.740 (0.120) | 0.107 | 0.685 | 0.768 | 0.823 | 1.134 | 0.563 | 0.276 | 0.19924 | 0.14492 | 48% | 85% |
| CV Average | | 0.735 (0.125) | 0.085 | 0.680 | 0.767 | 0.823 | 1.008 | 0.555 | 0.286 | 0.20735 | 0.14649 | 48% | 85% |
|  | |  |  |  |  |  |  |  |  |  |  |  |  |
| CLAD (11) | |  |  |  |  |  |  |  |  |  |  |  |  |
| fold 1 | | 0.735 (0.121) | 0.231 | 0.678 | 0.770 | 0.829 | 0.897 | 0.523 | 0.225 | 0.22971 | 0.14917 | 47% | 85% |
| fold 2 | | 0.741 (0.103) | 0.211 | 0.690 | 0.767 | 0.822 | 0.913 | 0.537 | 0.211 | 0.20514 | 0.14535 | 49% | 85% |
| fold 3 | | 0.737 (0.103) | 0.241 | 0.682 | 0.762 | 0.815 | 0.916 | 0.519 | 0.262 | 0.20603 | 0.16367 | 48% | 84% |
| fold 4 | | 0.731 (0.109) | 0.199 | 0.679 | 0.761 | 0.811 | 0.915 | 0.524 | 0.136 | 0.20118 | 0.14948 | 48% | 85% |
| fold 5 | | 0.729 (0.120) | 0.127 | 0.667 | 0.761 | 0.821 | 0.911 | 0.544 | 0.220 | 0.21380 | 0.16221 | 47% | 84% |
| fold 6 | | 0.728 (0.124) | 0.079 | 0.668 | 0.764 | 0.822 | 0.957 | 0.528 | 0.356 | 0.17616 | 0.15521 | 45% | 84% |
| fold 7 | | 0.740 (0.100) | 0.259 | 0.685 | 0.764 | 0.813 | 0.930 | 0.514 | 0.261 | 0.22743 | 0.13816 | 49% | 84% |
| fold 8 | | 0.731 (0.125) | 0.123 | 0.666 | 0.765 | 0.824 | 0.919 | 0.531 | 0.323 | 0.21463 | 0.14834 | 46% | 83% |
| fold 9 | | 0.740 (0.114) | 0.161 | 0.678 | 0.764 | 0.833 | 0.929 | 0.541 | 0.337 | 0.20866 | 0.14617 | 47% | 86% |
| fold 10 | | 0.740 (0.105) | 0.185 | 0.688 | 0.774 | 0.819 | 0.876 | 0.525 | 0.376 | 0.20732 | 0.13528 | 47% | 85% |
| CV Average | | 0.735 (0.112) | 0.182 | 0.678 | 0.765 | 0.821 | 0.916 | 0.528 | 0.271 | 0.20901 | 0.14930 | 47% | 84% |
|  | |  |  |  |  |  |  |  |  |  |  |  |  |
|  | |  |  |  |  |  |  |  |  |  |  |  |  |
|  | |  |  |  |  |  |  |  |  |  |  |  |  |
|  | |  |  |  |  |  |  |  |  |  |  |  |  |
| Estimation method^a^ | | Mean (SD) | Min. | P.25 | Median | P.75 | Max. | Corr | R^2^ | RMSE | MAE | % <0.10 | % <0.25 |
| BB (1) | |  |  |  |  |  |  |  |  |  |  |  |  |
| fold 1 | | 0.708 (0.109) | 0.223 | 0.670 | 0.751 | 0.787 | 0.796 | 0.499 | 0.295 | 0.21526 | 0.15569 | 49% | 84% |
| fold 2 | | 0.709 (0.110) | 0.110 | 0.670 | 0.753 | 0.789 | 0.798 | 0.507 | 0.127 | 0.21528 | 0.15530 | 49% | 84% |
| fold 3 | | 0.704 (0.106) | 0.127 | 0.663 | 0.742 | 0.781 | 0.791 | 0.481 | 0.219 | 0.21568 | 0.15590 | 48% | 82% |
| fold 4 | | 0.703 (0.109) | 0.115 | 0.666 | 0.742 | 0.782 | 0.792 | 0.494 | 0.249 | 0.21545 | 0.15610 | 48% | 83% |
| fold 5 | | 0.706 (0.110) | 0.115 | 0.668 | 0.744 | 0.784 | 0.794 | 0.503 | 0.400 | 0.21540 | 0.15575 | 49% | 83% |
| fold 6 | | 0.707 (0.110) | 0.107 | 0.670 | 0.745 | 0.786 | 0.796 | 0.499 | 0.120 | 0.21526 | 0.15574 | 99% | 83% |
| fold 7 | | 0.703 (0.108) | 0.123 | 0.663 | 0.743 | 0.783 | 0.792 | 0.485 | 0.292 | 0.21557 | 0.15574 | 47% | 82% |
| fold 8 | | 0.702 (0.111) | 0.119 | 0.658 | 0.745 | 0.785 | 0.795 | 0.497 | 0.156 | 0.21547 | 0.15544 | 47% | 83% |
| fold 9 | | 0.706 (0.109) | 0.115 | 0.669 | 0.744 | 0.785 | 0.794 | 0.493 | 0.276 | 0.21539 | 0.15565 | 48% | 84% |
| fold 10 | | 0.705 (0.111) | 0.115 | 0.661 | 0.743 | 0.784 | 0.793 | 0.497 | 0.186 | 0.21542 | 0.15583 | 48% | 83% |
| CV Average | | 0.705 (0.109) | 0.127 | 0.666 | 0.745 | 0.785 | 0.794 | 0.495 | 0.232 | 0.21542 | 0.15571 | 53% | 83% |
|  | |  |  |  |  |  |  |  |  |  |  |  |  |
|  | |  |  |  |  |  |  |  |  |  |  |  |  |
| Estimation method^a^ | | Mean (SD) | Min. | P.25 | Median | P.75 | Max. | Corr | R^2^ | RMSE | MAE | % <0.10 | % <0.25 |
| BB (2) | |  |  |  |  |  |  |  |  |  |  |  |  |
| fold 1 | | 0.708 (0.114) | 0.238 | 0.664 | 0.747 | 0.778 | 0.828 | 0.524 | 0.381 | 0.21117 | 0.15243 | 48% | 84% |
| fold 2 | | 0.709 (0.115) | 0.102 | 0.670 | 0.747 | 0.783 | 0.828 | 0.529 | 0.219 | 0.21131 | 0.15203 | 50% | 84% |
| fold 3 | | 0.704 (0.112) | 0.120 | 0.661 | 0.741 | 0.775 | 0.826 | 0.510 | 0.192 | 0.21151 | 0.15247 | 48% | 83% |
| fold 4 | | 0.703 (0.107) | 0.107 | 0.664 | 0.741 | 0.776 | 0.825 | 0.518 | 0.160 | 0.21141 | 0.15266 | 48% | 84% |
| fold 5 | | 0.706 (0.114) | 0.108 | 0.667 | 0.744 | 0.780 | 0.826 | 0.528 | 0.327 | 0.21134 | 0.15231 | 49% | 83% |
| fold 6 | | 0.706 (0.115) | 0.101 | 0.668 | 0.743 | 0.780 | 0.829 | 0.525 | 0.289 | 0.21114 | 0.15230 | 49% | 83% |
| fold 7 | | 0.702 (0.113) | 0.111 | 0.657 | 0.738 | 0.773 | 0.824 | 0.512 | 0.401 | 0.21152 | 0.15275 | 48% | 83% |
| fold 8 | | 0.702 (0.116) | 0.108 | 0.658 | 0.740 | 0.776 | 0.826 | 0.522 | 0.230 | 0.21144 | 0.15248 | 47% | 83% |
| fold 9 | | 0.706 (0.115) | 0.108 | 0.665 | 0.742 | 0.780 | 0.831 | 0.523 | 0.216 | 0.21113 | 0.15205 | 49% | 84% |
| fold 10 | | 0.704 (0.116) | 0.105 | 0.663 | 0.745 | 0.775 | 0.829 | 0.527 | 0.232 | 0.21120 | 0.15244 | 48% | 83% |
| CV Average | | 0.705 (0.114) | 0.121 | 0.664 | 0.743 | 0.778 | 0.827 | 0.522 | 0.265 | 0.21132 | 0.15239 | 48% | 83% |
|  | |  |  |  |  |  |  |  |  |  |  |  |  |
| Estimation method^a^ | | Mean (SD) | Min. | P.25 | Median | P.75 | Max. | Corr | R^2^ | RMSE | MAE | % <0.10 | % <0.25 |
| BB (3) | |  |  |  |  |  |  |  |  |  |  |  |  |
| fold 1 | | 0.708 (0.109) | 0.217 | 0.669 | 0.751 | 0.786 | 0.800 | 0.503 | 0.239 | 0.21472 | 0.15500 | 48% | 84% |
| fold 2 | | 0.709 (0.111) | 0.120 | 0.667 | 0.754 | 0.787 | 0.802 | 0.511 | 0.205 | 0.21475 | 0.15456 | 50% | 84% |
| fold 3 | | 0.704 (0.107) | 0.144 | 0.664 | 0.744 | 0.780 | 0.796 | 0.487 | 0.194 | 0.21510 | 0.15498 | 48% | 83% |
| fold 4 | | 0.703 (0.109) | 0.126 | 0.662 | 0.743 | 0.779 | 0.796 | 0.509 | 0.165 | 0.21492 | 0.15536 | 48% | 83% |
| fold 5 | | 0.706 (0.110) | 0.129 | 0.667 | 0.746 | 0.785 | 0.799 | 0.509 | 0.270 | 0.21484 | 0.15496 | 48% | 83% |
| fold 6 | | 0.707 (0.111) | 0.119 | 0.665 | 0.748 | 0.783 | 0.801 | 0.503 | 0.299 | 0.21469 | 0.15496 | 48% | 83% |
| fold 7 | | 0.703 (0.109) | 0.132 | 0.662 | 0.743 | 0.780 | 0.796 | 0.489 | 0.292 | 0.21510 | 0.15509 | 48% | 83% |
| fold 8 | | 0.703 (0.111) | 0.128 | 0.660 | 0.744 | 0.782 | 0.798 | 0.500 | 0.165 | 0.21498 | 0.15478 | 47% | 83% |
| fold 9 | | 0.706 (0.110) | 0.130 | 0.664 | 0.743 | 0.783 | 0.801 | 0.500 | 0.247 | 0.21477 | 0.15475 | 48% | 84% |
| fold 10 | | 0.705 (0.111) | 0.127 | 0.661 | 0.748 | 0.781 | 0.798 | 0.501 | 0.305 | 0.21486 | 0.15503 | 47% | 83% |
| CV Average | | 0.705 (0.110) | 0.137 | 0.664 | 0.746 | 0.783 | 0.799 | 0.501 | 0.238 | 0.21487 | 0.15495 | 48% | 83% |
|  | |  |  |  |  |  |  |  |  |  |  |  |  |
| Estimation method^a^ | | Mean (SD) | Min. | P.25 | Median | P.75 | Max. | Corr | R^2^ | RMSE | MAE | % <0.10 | % <0.25 |
| BB (4) | |  |  |  |  |  |  |  |  |  |  |  |  |
| fold 1 | | 0.708 (0.113) | 0.245 | 0.667 | 0.747 | 0.773 | 0.823 | 0.520 | 0.173 | 0.21201 | 0.15374 | 47% | 83% |
| fold 2 | | 0.709 (0.114) | 0.092 | 0.669 | 0.746 | 0.778 | 0.822 | 0.523 | 0.318 | 0.21209 | 0.15322 | 48% | 84% |
| fold 3 | | 0.704 (0.111) | 0.104 | 0.661 | 0.742 | 0.768 | 0.818 | 0.502 | 0.285 | 0.21234 | 0.15391 | 47% | 82% |
| fold 4 | | 0.703 (0.113) | 0.097 | 0.663 | 0.740 | 0.772 | 0.819 | 0.512 | 0.228 | 0.21217 | 0.15386 | 47% | 83% |
| fold 5 | | 0.706 (0.114) | 0.095 | 0.669 | 0.742 | 0.774 | 0.820 | 0.522 | 0.263 | 0.21214 | 0.15358 | 48% | 83% |
| fold 6 | | 0.706 (0.114) | 0.089 | 0.668 | 0.743 | 0.774 | 0.823 | 0.519 | 0.364 | 0.21196 | 0.15357 | 47% | 82% |
| fold 7 | | 0.702 (0.113) | 0.101 | 0.660 | 0.738 | 0.769 | 0.819 | 0.508 | 0.216 | 0.21224 | 0.15384 | 47% | 82% |
| fold 8 | | 0.702 (0.116) | 0.099 | 0.656 | 0.740 | 0.771 | 0.821 | 0.517 | 0.291 | 0.21216 | 0.15357 | 46% | 82% |
| fold 9 | | 0.706 (0.113) | 0.094 | 0.666 | 0.740 | 0.772 | 0.823 | 0.514 | 0.163 | 0.21203 | 0.15356 | 48% | 83% |
| fold 10 | | 0.704 (0.116) | 0.094 | 0.665 | 0.743 | 0.770 | 0.823 | 0.521 | 0.271 | 0.21201 | 0.15374 | 46% | 83% |
| CV Average | | 0.705 (0.114) | 0.111 | 0.665 | 0.742 | 0.772 | 0.821 | 0.516 | 0.257 | 0.21212 | 0.15366 | 47% | 83% |
|  | |  |  |  |  |  |  |  |  |  |  |  |  |
|  | |  |  |  |  |  |  |  |  |  |  |  |  |
| Estimation method^a^ | | Mean (SD) | Min. | P.25 | Median | P.75 | Max. | Corr | R^2^ | RMSE | MAE | % <0.10 | % <0.25 |
| BB (5) | |  |  |  |  |  |  |  |  |  |  |  |  |
| fold 1 | | 0.707 (0.114) | 0.340 | 0.644 | 0.745 | 0.791 | 0.839 | 0.518 | 0.268 | 0.21160 | 0.15286 | 48% | 83% |
| fold 2 | | 0.709 (0.115) | 0.390 | 0.637 | 0.744 | 0.804 | 0.844 | 0.518 | 0.268 | 0.21269 | 0.15305 | 48% | 84% |
| fold 3 | | 0.704 (0.113) | 0.394 | 0.631 | 0.737 | 0.796 | 0.842 | 0.502 | 0.196 | 0.21295 | 0.15331 | 46% | 83% |
| fold 4 | | 0.703 (0.114) | 0.394 | 0.634 | 0.737 | 0.797 | 0.842 | 0.512 | 0.248 | 0.21292 | 0.15355 | 46% | 83% |
| fold 5 | | 0.705 (0.114) | 0.386 | 0.637 | 0.741 | 0.800 | 0.842 | 0.518 | 0.203 | 0.21261 | 0.15323 | 47% | 83% |
| fold 6 | | 0.706 (0.114) | 0.378 | 0.639 | 0.741 | 0.797 | 0.843 | 0.513 | 0.247 | 0.21236 | 0.15311 | 46% | 83% |
| fold 7 | | 0.702 (0.113) | 0.396 | 0.626 | 0.733 | 0.794 | 0.841 | 0.506 | 0.405 | 0.21288 | 0.15342 | 46% | 82% |
| fold 8 | | 0.702 (0.116) | 0.389 | 0.625 | 0.734 | 0.796 | 0.843 | 0.511 | 0.306 | 0.21262 | 0.15318 | 46% | 82% |
| fold 9 | | 0.705 (0.115) | 0.380 | 0.634 | 0.739 | 0.800 | 0.848 | 0.512 | 0.408 | 0.21261 | 0.15310 | 47% | 83% |
| fold 10 | | 0.704 (0.116) | 0.382 | 0.631 | 0.740 | 0.796 | 0.845 | 0.517 | 0.129 | 0.21256 | 0.15344 | 46% | 83% |
| CV Average | | 0.705 (0.114) | 0.383 | 0.634 | 0.739 | 0.797 | 0.843 | 0.513 | 0.268 | 0.21258 | 0.15322 | 47% | 83% |
|  | |  |  |  |  |  |  |  |  |  |  |  |  |
| Estimation method^a^ | | Mean (SD) | Min. | P.25 | Median | P.75 | Max. | Corr | R^2^ | RMSE | MAE | % <0.10 | % <0.25 |
| BB (6) | |  |  |  |  |  |  |  |  |  |  |  |  |
| fold 1 | | 0.707 (0.114) | 0.341 | 0.640 | 0.743 | 0.793 | 0.840 | 0.519 | 0.256 | 0.21162 | 0.15296 | 47% | 83% |
| fold 2 | | 0.709 (0.114) | 0.393 | 0.634 | 0.742 | 0.805 | 0.845 | 0.518 | 0.324 | 0.21267 | 0.15316 | 47% | 84% |
| fold 3 | | 0.704 (0.112) | 0.401 | 0.627 | 0.735 | 0.798 | 0.844 | 0.502 | 0.238 | 0.21298 | 0.15348 | 46% | 83% |
| fold 4 | | 0.703 (0.113) | 0.398 | 0.632 | 0.734 | 0.799 | 0.843 | 0.511 | 0.178 | 0.21288 | 0.15367 | 46% | 83% |
| fold 5 | | 0.705 (0.113) | 0.387 | 0.636 | 0.739 | 0.801 | 0.843 | 0.518 | 0.190 | 0.21255 | 0.15332 | 46% | 83% |
| fold 6 | | 0.706 (0.114) | 0.374 | 0.637 | 0.739 | 0.798 | 0.844 | 0.514 | 0.181 | 0.21224 | 0.15316 | 46% | 82% |
| fold 7 | | 0.702 (0.112) | 0.397 | 0.624 | 0.730 | 0.796 | 0.842 | 0.506 | 0.275 | 0.21281 | 0.15352 | 46% | 82% |
| fold 8 | | 0.702 (0.115) | 0.389 | 0.623 | 0.731 | 0.798 | 0.844 | 0.512 | 0.300 | 0.21256 | 0.15327 | 45% | 84% |
| fold 9 | | 0.705 (0.114) | 0.386 | 0.632 | 0.736 | 0.802 | 0.849 | 0.512 | 0.426 | 0.21261 | 0.15325 | 47% | 84% |
| fold 10 | | 0.704 (0.116) | 0.384 | 0.629 | 0.738 | 0.797 | 0.846 | 0.517 | 0.297 | 0.21253 | 0.15358 | 46% | 83% |
| CV Average | | 0.704 (0.114) | 0.385 | 0.631 | 0.737 | 0.799 | 0.844 | 0.513 | 0.266 | 0.21254 | 0.15334 | 46% | 83% |
|  | |  |  |  |  |  |  |  |  |  |  |  |  |
|  | |  |  |  |  |  |  |  |  |  |  |  |  |
| Estimation method^a^ | | Mean (SD) | Min. | P.25 | Median | P.75 | Max. | Corr | R^2^ | RMSE | MAE | % <0.10 | % <0.25 |
| BB (7) | |  |  |  |  |  |  |  |  |  |  |  |  |
| fold 1 | | 0.708 (0.122) | 0.267 | 0.631 | 0.737 | 0.814 | 0.881 | 0.545 | 0.248 | 0.20929 | 0.15211 | 45% | 85% |
| fold 2 | | 0.710 (0.122) | 0.216 | 0.630 | 0.745 | 0.809 | 0.885 | 0.559 | 0.308 | 0.20730 | 0.15054 | 45% | 85% |
| fold 3 | | 0.704 (0.120) | 0.242 | 0.628 | 0.732 | 0.812 | 0.897 | 0.538 | 0.238 | 0.20799 | 0.15175 | 44% | 83% |
| fold 4 | | 0.704 (0.122) | 0.210 | 0.626 | 0.735 | 0.810 | 0.891 | 0.551 | 0.136 | 0.20749 | 0.15150 | 44% | 83% |
| fold 5 | | 0.706 (0.125) | 0.232 | 0.632 | 0.739 | 0.813 | 0.905 | 0.564 | 0.127 | 0.20781 | 0.15060 | 45% | 84% |
| fold 6 | | 0.707 (0.122) | 0.176 | 0.633 | 0.736 | 0.808 | 0.888 | 0.549 | 0.153 | 0.20722 | 0.15097 | 44% | 84% |
| fold 7 | | 0.703 (0.121) | 0.223 | 0.628 | 0.730 | 0.808 | 0.908 | 0.540 | 0.213 | 0.20748 | 0.15097 | 44% | 83% |
| fold 8 | | 0.703 (0.124) | 0.230 | 0.625 | 0.732 | 0.805 | 0.892 | 0.551 | 0.319 | 0.20752 | 0.15079 | 44% | 83% |
| fold 9 | | 0.706 (0.121) | 0.255 | 0.631 | 0.736 | 0.812 | 0.872 | 0.547 | 0.343 | 0.20813 | 0.15084 | 45% | 85% |
| fold 10 | | 0.705 (0.124) | 0.213 | 0.626 | 0.738 | 0.815 | 0.879 | 0.551 | 0.270 | 0.20719 | 0.15148 | 44% | 84% |
| CV Average | | 0.706 (0.122) | 0.226 | 0.629 | 0.736 | 0.811 | 0.890 | 0.549 | 0.236 | 0.20774 | 0.15115 | 44% | 84% |
|  | |  |  |  |  |  |  |  |  |  |  |  |  |
|  | |  |  |  |  |  |  |  |  |  |  |  |  |
| Estimation method^a^ | | Mean (SD) | Min. | P.25 | Median | P.75 | Max. | Corr | R^2^ | RMSE | MAE | % <0.10 | % <0.25 |
| BB (8) | |  |  |  |  |  |  |  |  |  |  |  |  |
| fold 1 | | 0.707 (0.125) | 0.245 | 0.632 | 0.738 | 0.812 | 0.897 | 0.563 | 0.187 | 0.20604 | 0.15035 | 45% | 85% |
| fold 2 | | 0.710 (0.125) | 0.216 | 0.634 | 0.744 | 0.809 | 0.898 | 0.575 | 0.184 | 0.20411 | 0.14849 | 47% | 85% |
| fold 3 | | 0.704 (0.124) | 0.239 | 0.626 | 0.735 | 0.811 | 0.919 | 0.561 | 0.253 | 0.20461 | 0.14951 | 44% | 84% |
| fold 4 | | 0.703 (0.126) | 0.216 | 0.626 | 0.735 | 0.805 | 0.908 | 0.569 | 0.175 | 0.20423 | 0.14937 | 44% | 84% |
| fold 5 | | 0.706 (0.128) | 0.224 | 0.630 | 0.744 | 0.809 | 0.924 | 0.581 | 0.383 | 0.20455 | 0.14863 | 46% | 84% |
| fold 6 | | 0.707 (0.126) | 0.175 | 0.634 | 0.736 | 0.810 | 0.910 | 0.569 | 0.130 | 0.20388 | 0.14881 | 45% | 84% |
| fold 7 | | 0.703 (0.125) | 0.226 | 0.627 | 0.733 | 0.806 | 0.919 | 0.559 | 0.431 | 0.20420 | 0.14915 | 44% | 84% |
| fold 8 | | 0.702 (0.128) | 0.235 | 0.627 | 0.738 | 0.807 | 0.915 | 0.569 | 0.164 | 0.20431 | 0.14915 | 45% | 84% |
| fold 9 | | 0.706 (0.125) | 0.253 | 0.632 | 0.734 | 0.815 | 0.901 | 0.569 | 0.365 | 0.20467 | 0.14887 | 46% | 86% |
| fold 10 | | 0.705 (0.128) | 0.217 | 0.628 | 0.743 | 0.813 | 0.906 | 0.572 | 0.377 | 0.20378 | 0.14960 | 44% | 85% |
| CV Average | | 0.705 (0.126) | 0.225 | 0.629 | 0.738 | 0.810 | 0.910 | 0.569 | 0.265 | 0.20444 | 0.14919 | 45% | 85% |
|  | |  |  |  |  |  |  |  |  |  |  |  |  |
| Estimation method^a^ | | Mean (SD) | Min. | P.25 | Median | P.75 | Max. | Corr | R^2^ | RMSE | MAE | % <0.10 | % <0.25 |
| BB (9) | |  |  |  |  |  |  |  |  |  |  |  |  |
| fold 1 | | 0.707 (0.123) | 0.246 | 0.644 | 0.741 | 0.797 | 0.852 | 0.549 | 0.263 | 0.20656 | 0.15111 | 47% | 85% |
| fold 2 | | 0.710 (0.123) | 0.210 | 0.650 | 0.745 | 0.796 | 0.922 | 0.559 | 0.303 | 0.20563 | 0.14460 | 48% | 84% |
| fold 3 | | 0.704 (0.121) | 0.243 | 0.639 | 0.734 | 0.790 | 0.918 | 0.541 | 0.272 | 0.20616 | 0.14247 | 45% | 83% |
| fold 4 | | 0.703 (0.123) | 0.231 | 0.639 | 0.734 | 0.793 | 0.919 | 0.551 | 0.324 | 0.20585 | 0.14804 | 46% | 83% |
| fold 5 | | 0.706 (0.123) | 0.229 | 0.643 | 0.742 | 0.794 | 0.920 | 0.560 | 0.246 | 0.20587 | 0.13075 | 47% | 83% |
| fold 6 | | 0.707 (0.122) | 0.185 | 0.646 | 0.736 | 0.791 | 0.920 | 0.552 | 0.291 | 0.20528 | 0.13383 | 46% | 83% |
| fold 7 | | 0.702 (0.122) | 0.240 | 0.635 | 0.734 | 0.790 | 0.918 | 0.542 | 0.412 | 0.20595 | 0.14930 | 83% | 45% |
| fold 8 | | 0.702 (0.126) | 0.245 | 0.637 | 0.738 | 0.794 | 0.920 | 0.554 | 0.295 | 0.15047 | 0.13556 | 45% | 83% |
| fold 9 | | 0.706 (0.124) | 0.223 | 0.640 | 0.736 | 0.794 | 0.922 | 0.558 | 0.310 | 0.20559 | 0.14777 | 46% | 84% |
| fold 10 | | 0.705 (0.126) | 0.236 | 0.636 | 0.739 | 0.793 | 0.919 | 0.560 | 0.193 | 0.19971 | 0.14970 | 46% | 84% |
| CV Average | | 0.705 (0.123) | 0.229 | 0.641 | 0.738 | 0.793 | 0.913 | 0.553 | 0.291 | 0.19971 | 0.14331 | 50% | 80% |
|  | |  |  |  |  |  |  |  |  |  |  |  |  |
|  | |  |  |  |  |  |  |  |  |  |  |  |  |
| Estimation method^a^ | | Mean (SD) | Min. | P.25 | Median | P.75 | Max. | Corr | R^2^ | RMSE | MAE | % <0.10 | % <0.25 |
| BB (10) | |  |  |  |  |  |  |  |  |  |  |  |  |
| fold 1 | | 0.708 (0.122) | 0.231 | 0.649 | 0.742 | 0.799 | 0.859 | 0.548 | 0.276 | 0.20740 | 0.15021 | 46% | 85% |
| fold 2 | | 0.710 (0.123) | 0.197 | 0.651 | 0.743 | 0.799 | 0.885 | 0.560 | 0.229 | 0.20584 | 0.14918 | 47% | 85% |
| fold 3 | | 0.705 (0.120) | 0.225 | 0.644 | 0.738 | 0.795 | 0.883 | 0.541 | 0.390 | 0.20626 | 0.14996 | 45% | 84% |
| fold 4 | | 0.704 (0.122) | 0.187 | 0.646 | 0.737 | 0.793 | 0.874 | 0.551 | 0.182 | 0.20579 | 0.14970 | 46% | 84% |
| fold 5 | | 0.706 (0.124) | 0.203 | 0.649 | 0.742 | 0.795 | 0.883 | 0.561 | 0.383 | 0.20615 | 0.14948 | 46% | 85% |
| fold 6 | | 0.707 (0.122) |  | 0.654 | 0.739 | 0.795 | 0.882 | 0.552 | 0.365 | 0.20560 | 0.14934 | 46% | 84% |
| fold 7 | | 0.703 (0.122) | 0.232 | 0.643 | 0.736 | 0.794 | 0.887 | 0.544 | 0.277 | 0.20605 | 0.14992 | 45% | 84% |
| fold 8 | | 0.703 (0.124) | 0.202 | 0.644 | 0.737 | 0.797 | 0.877 | 0.552 | 0.249 | 0.20593 | 0.14948 | 45% | 84% |
| fold 9 | | 0.706 (0.123) | 0.207 | 0.648 | 0.742 | 0.800 | 0.878 | 0.556 | 0.292 | 0.20595 | 0.14935 | 46% | 85% |
| fold 10 | | 0.705 (0.126) | 0.210 | 0.641 | 0.737 | 0.796 | 0.879 | 0.559 | 0.166 | 0.20596 | 0.15006 | 45% | 84% |
| CV Average | | 0.706 (0.123) | 0.210 | 0.647 | 0.739 | 0.796 | 0.879 | 0.552 | 0.281 | 0.20609 | 0.14967 | 46% | 84% |
|  | |  |  |  |  |  |  |  |  |  |  |  |  |
| Estimation method^a^ | | Mean (SD) | Min. | P.25 | Median | P.75 | Max. | Corr | R^2^ | RMSE | MAE | % <0.10 | % <0.25 |
| BB (11) | |  |  |  |  |  |  |  |  |  |  |  |  |
| fold 1 | | 0.707 (0.119) | 0.282 | 0.627 | 0.733 | 0.811 | 0.854 | 0.542 | 0.284 | 0.20866 | 0.15200 | 45% | 85% |
| fold 2 | | 0.708 (0.119) | 0.276 | 0.627 | 0.738 | 0.808 | 0.853 | 0.542 | 0.365 | 0.20893 | 0.15134 | 46% | 85% |
| fold 3 | | 0.703 (0.117) | 0.280 | 0.623 | 0.724 | 0.810 | 0.854 | 0.530 | 0.235 | 0.20930 | 0.15211 | 44% | 84% |
| fold 4 | | 0.702 (0.118) | 0.290 | 0.622 | 0.728 | 0.808 | 0.853 | 0.534 | 0.242 | 0.20895 | 0.15187 | 44% | 84% |
| fold 5 | | 0.705 (0.119) | 0.281 | 0.621 | 0.732 | 0.809 | 0.855 | 0.545 | 0.259 | 0.20879 | 0.15158 | 46% | 84% |
| fold 6 | | 0.706 (0.119) | 0.236 | 0.629 | 0.729 | 0.808 | 0.855 | 0.540 | 0.218 | 0.20850 | 0.15160 | 44% | 84% |
| fold 7 | | 0.701 (0.117) | 0.292 | 0.620 | 0.727 | 0.803 | 0.854 | 0.528 | 0.260 | 0.20892 | 0.15179 | 44% | 83% |
| fold 8 | | 0.701 (0.120) | 0.278 | 0.621 | 0.726 | 0.809 | 0.854 | 0.539 | 0.312 | 0.20890 | 0.15171 | 44% | 84% |
| fold 9 | | 0.705 (0.119) | 0.301 | 0.618 | 0.729 | 0.816 | 0.861 | 0.540 | 0.194 | 0.20906 | 0.15165 | 44% | 84% |
| fold 10 | | 0.704 (0.121) | 0.290 | 0.621 | 0.735 | 0.809 | 0.858 | 0.543 | 0.318 | 0.20865 | 0.15223 | 44% | 83% |
| CV Average | | 0.704 (0.119) | 0.281 | 0.623 | 0.730 | 0.809 | 0.855 | 0.538 | 0.269 | 0.20886 | 0.15179 | 45% | 84% |
|  | |  |  |  |  |  |  |  |  |  |  |  |  |
| Estimation method^a^ | | Mean (SD) | Min. | P.25 | Median | P.75 | Max. | Corr | R^2^ | RMSE | MAE | % <0.10 | % <0.25 |
| GLM (1) | |  |  |  |  |  |  |  |  |  |  |  |  |
| fold 1 | | 0.711 (0.123) | 0.354 | 0.627 | 0.741 | 0.818 | 0.843 | 0.493 | 0.189 | 0.21550 | 0.15589 | 44% | 83% |
| fold 2 | | 0.712 (0.126) | 0.297 | 0.626 | 0.744 | 0.822 | 0.847 | 0.503 | 0.277 | 0.21554 | 0.15570 | 44% | 84% |
| fold 3 | | 0.709 (0.121) | 0.307 | 0.623 | 0.733 | 0.815 | 0.839 | 0.481 | 0.381 | 0.21538 | 0.15513 | 43% | 83% |
| fold 4 | | 0.708 (0.124) | 0.299 | 0.624 | 0.731 | 0.817 | 0.841 | 0.492 | 0.172 | 0.21545 | 0.15573 | 43% | 83% |
| fold 5 | | 0.711 (0.126) | 0.297 | 0.625 | 0.733 | 0.821 | 0.846 | 0.497 | 0.300 | 0.21553 | 0.15580 | 43% | 82% |
| fold 6 | | 0.711 (0.125) | 0.296 | 0.627 | 0.733 | 0.821 | 0.846 | 0.489 | 0.317 | 0.21555 | 0.15591 | 43% | 82% |
| fold 7 | | 0.708 (0.122) | 0.306 | 0.624 | 0.735 | 0.818 | 0.842 | 0.483 | 0.249 | 0.21542 | 0.15504 | 43% | 83% |
| fold 8 | | 0.705 (0.127) | 0.300 | 0.613 | 0.734 | 0.821 | 0.846 | 0.491 | 0.208 | 0.21548 | 0.15565 | 42% | 82% |
| fold 9 | | 0.710 (0.122) | 0.303 | 0.628 | 0.733 | 0.818 | 0.842 | 0.490 | 0.283 | 0.21542 | 0.15533 | 43% | 84% |
| fold 10 | | 0.709 (0.127) | 0.296 | 0.616 | 0.732 | 0.820 | 0.845 | 0.494 | 0.124 | 0.21553 | 0.15593 | 42% | 82% |
| CV Average | | 0.709 (0.124) | 0.305 | 0.623 | 0.735 | 0.819 | 0.844 | 0.491 | 0.250 | 0.21548 | 0.15561 | 43% | 83% |
|  | |  |  |  |  |  |  |  |  |  |  |  |  |
|  | |  |  |  |  |  |  |  |  |  |  |  |  |
| Estimation method^a^ | | Mean (SD) | Min. | P.25 | Median | P.75 | Max. | Corr | R^2^ | RMSE | MAE | % <0.10 | % <0.25 |
| GLM (2) | |  |  |  |  |  |  |  |  |  |  |  |  |
| fold 1 | | 0.711 (0.130) | 0.359 | 0.624 | 0.733 | 0.807 | 0.910 | 0.518 | 0.274 | 0.21153 | 0.15474 | 48% | 83% |
| fold 2 | | 0.712 (0.131) | 0.296 | 0.620 | 0.732 | 0.819 | 0.911 | 0.523 | 0.175 | 0.21167 | 0.15412 | 48% | 83% |
| fold 3 | | 0.709 (0.129) | 0.306 | 0.620 | 0.731 | 0.806 | 0.911 | 0.507 | 0.282 | 0.21153 | 0.15414 | 47% | 82% |
| fold 4 | | 0.708 (0.130) | 0.298 | 0.617 | 0.728 | 0.812 | 0.911 | 0.513 | 0.299 | 0.21157 | 0.15434 | 48% | 85% |
| fold 5 | | 0.711 (0.132) | 0.296 | 0.620 | 0.734 | 0.817 | 0.916 | 0.519 | 0.439 | 0.21170 | 0.15432 | 47% | 82% |
| fold 6 | | 0.711 (0.132) | 0.294 | 0.623 | 0.732 | 0.812 | 0.918 | 0.515 | 0.181 | 0.21170 | 0.15432 | 46% | 82% |
| fold 7 | | 0.708 (0.130) | 0.304 | 0.618 | 0.726 | 0.805 | 0.912 | 0.508 | 0.337 | 0.21144 | 0.15410 | 47% | 83% |
| fold 8 | | 0.705 (0.133) | 0.297 | 0.614 | 0.725 | 0.808 | 0.915 | 0.515 | 0.157 | 0.21152 | 0.15445 | 46% | 82% |
| fold 9 | | 0.710 (0.130) | 0.301 | 0.621 | 0.728 | 0.811 | 0.918 | 0.518 | 0.323 | 0.21163 | 0.15433 | 47% | 83% |
| fold 10 | | 0.709 (0.135) | 0.293 | 0.618 | 0.732 | 0.807 | 0.922 | 0.521 | 0.196 | 0.21165 | 0.15495 | 46% | 83% |
| CV Average | | 0.709 (0.131) | 0.304 | 0.619 | 0.730 | 0.811 | 0.915 | 0.516 | 0.266 | 0.21159 | 0.15438 | 47% | 83% |
|  | |  |  |  |  |  |  |  |  |  |  |  |  |
| Estimation method^a^ | | Mean (SD) | Min. | P.25 | Median | P.75 | Max. | Corr | R^2^ | RMSE | MAE | % <0.10 | % <0.25 |
| GLM (3) | |  |  |  |  |  |  |  |  |  |  |  |  |
| fold 1 | | 0.711 (0.124) | 0.352 | 0.624 | 0.739 | 0.815 | 0.852 | 0.496 | 0.227 | 0.21510 | 0.15566 | 44% | 83% |
| fold 2 | | 0.712 (0.127) | 0.303 | 0.621 | 0.745 | 0.817 | 0.858 | 0.505 | 0.226 | 0.21517 | 0.15544 | 44% | 84% |
| fold 3 | | 0.709 (0.123) | 0.314 | 0.621 | 0.735 | 0.808 | 0.851 | 0.484 | 0.245 | 0.21508 | 0.15509 | 43% | 83% |
| fold 4 | | 0.708 (0.125) | 0.305 | 0.620 | 0.732 | 0.807 | 0.853 | 0.494 | 0.152 | 0.21510 | 0.15558 | 43% | 83% |
| fold 5 | | 0.711 (0.303) | 0.303 | 0.624 | 0.739 | 0.820 | 0.858 | 0.500 | 0.270 | 0.21520 | 0.15564 | 44% | 82% |
| fold 6 | | 0.711 (0.126) | 0.301 | 0.619 | 0.742 | 0.811 | 0.857 | 0.493 | 0.297 | 0.21519 | 0.15570 | 43% | 83% |
| fold 7 | | 0.708 (0.123) | 0.312 | 0.621 | 0.735 | 0.808 | 0.852 | 0.485 | 0.287 | 0.21502 | 0.15483 | 43% | 83% |
| fold 8 | | 0.705 (0.128) | 0.305 | 0.615 | 0.732 | 0.811 | 0.856 | 0.493 | 0.225 | 0.21512 | 0.15527 | 42% | 82% |
| fold 9 | | 0.710 (0.124) | 0.310 | 0.619 | 0.736 | 0.808 | 0.855 | 0.494 | 0.249 | 0.21515 | 0.15530 | 44% | 84% |
| fold 10 | | 0.709 (0.128) | 0.301 | 0.617 | 0.742 | 0.810 | 0.856 | 0.496 | 0.268 | 0.21517 | 0.15571 | 43% | 83% |
| CV Average | | 0.709 (0.125) | 0.310 | 0.620 | 0.738 | 0.811 | 0.855 | 0.494 | 0.245 | 0.21513 | 0.15542 | 43% | 83% |
|  | |  |  |  |  |  |  |  |  |  |  |  |  |
|  | |  |  |  |  |  |  |  |  |  |  |  |  |
| Estimation method^a^ | | Mean (SD) | Min. | P.25 | Median | P.75 | Max. | Corr | R^2^ | RMSE | MAE | % <0.10 | % <0.25 |
| GLM (4) | |  |  |  |  |  |  |  |  |  |  |  |  |
| fold 1 | | 0.711 (0.129) | 0.361 | 0.618 | 0.734 | 0.799 | 0.897 | 0.515 | 0.378 | 0.21201 | 0.15556 | 43% | 83% |
| fold 2 | | 0.712 (0.130) | 0.291 | 0.623 | 0.732 | 0.809 | 0.896 | 0.521 | 0.219 | 0.21212 | 0.15507 | 44% | 83% |
| fold 3 | | 0.709 (0.127) | 0.298 | 0.622 | 0.733 | 0.795 | 0.893 | 0.503 | 0.179 | 0.21187 | 0.15494 | 43% | 82% |
| fold 4 | | 0.708 (0.128) | 0.292 | 0.621 | 0.729 | 0.802 | 0.895 | 0.510 | 0.202 | 0.21199 | 0.15521 | 43% | 82% |
| fold 5 | | 0.711 (0.131) | 0.289 | 0.621 | 0.738 | 0.807 | 0.900 | 0.515 | 0.321 | 0.21211 | 0.15522 | 42% | 83% |
| fold 6 | | 0.711 (0.131) | 0.288 | 0.619 | 0.731 | 0.802 | 0.902 | 0.511 | 0.277 | 0.21207 | 0.15547 | 47% | 82% |
| fold 7 | | 0.708 (0.128) | 0.298 | 0.623 | 0.726 | 0.796 | 0.898 | 0.506 | 0.375 | 0.21189 | 0.15490 | 42% | 82% |
| fold 8 | | 0.705 (0.132) | 0.292 | 0.613 | 0.724 | 0.799 | 0.901 | 0.514 | 0.238 | 0.21197 | 0.15528 | 46% | 82% |
| fold 9 | | 0.710 (0.128) | 0.294 | 0.621 | 0.734 | 0.799 | 0.899 | 0.513 | 0.234 | 0.21193 | 0.15508 | 43% | 83% |
| fold 10 | | 0.709 (0.134) | 0.288 | 0.616 | 0.732 | 0.797 | 0.905 | 0.518 | 0.240 | 0.21206 | 0.15577 | 47% | 83% |
| CV Average | | 0.709 (0.130) | 0.299 | 0.620 | 0.731 | 0.801 | 0.899 | 0.513 | 0.266 | 0.21200 | 0.15525 | 44% | 83% |
|  | |  |  |  |  |  |  |  |  |  |  |  |  |
| Estimation method^a^ | | Mean (SD) | Min. | P.25 | Median | P.75 | Max. | Corr | R^2^ | RMSE | MAE | % <0.10 | % <0.25 |
| GLM (5) | |  |  |  |  |  |  |  |  |  |  |  |  |
| fold 1 | | 0.711 (0.128) | 0.246 | 0.658 | 0.743 | 0.793 | 0.867 | 0.520 | 0.207 | 0.21135 | 0.15350 | 46% | 84% |
| fold 2 | | 0.712 (0.128) | 0.188 | 0.647 | 0.745 | 0.792 | 0.878 | 0.528 | 0.320 | 0.21099 | 0.15288 | 47% | 84% |
| fold 3 | | 0.709 (0.126) | 0.214 | 0.642 | 0.731 | 0.786 | 0.886 | 0.510 | 0.288 | 0.21090 | 0.15310 | 45% | 83% |
| fold 4 | | 0.708 (0.127) | 0.208 | 0.638 | 0.738 | 0.792 | 0.885 | 0.517 | 0.247 | 0.21091 | 0.15360 | 45% | 83% |
| fold 5 | | 0.711 (0.129) | 0.187 | 0.647 | 0.738 | 0.790 | 0.881 | 0.526 | 0.308 | 0.21101 | 0.15304 | 46% | 83% |
| fold 6 | | 0.711 (0.129) | 0.172 | 0.655 | 0.739 | 0.784 | 0.878 | 0.522 | 0.357 | 0.21116 | 0.15337 | 46% | 82% |
| fold 7 | | 0.708 (0.127) | 0.213 | 0.639 | 0.737 | 0.784 | 0.886 | 0.513 | 0.235 | 0.21076 | 0.15303 | 46% | 82% |
| fold 8 | | 0.705 (0.131) | 0.190 | 0.641 | 0.737 | 0.785 | 0.880 | 0.521 | 0.266 | 0.21087 | 0.15315 | 45% | 82% |
| fold 9 | | 0.710 (0.127) | 0.196 | 0.645 | 0.734 | 0.786 | 0.886 | 0.522 | 0.174 | 0.21098 | 0.15319 | 46% | 84% |
| fold 10 | | 0.709 (0.132) | 0.190 | 0.645 | 0.736 | 0.786 | 0.889 | 0.526 | 0.315 | 0.21087 | 0.15357 | 44% | 83% |
| CV Average | | 0.709 (0.128) | 0.200 | 0.646 | 0.738 | 0.788 | 0.882 | 0.520 | 0.272 | 0.21098 | 0.15324 | 45% | 83% |
|  | |  |  |  |  |  |  |  |  |  |  |  |  |
|  | |  |  |  |  |  |  |  |  |  |  |  |  |
| Estimation method^a^ | | Mean (SD) | Min. | P.25 | Median | P.75 | Max. | Corr | R^2^ | RMSE | MAE | % <0.10 | % <0.25 |
| GLM (6) | |  |  |  |  |  |  |  |  |  |  |  |  |
| fold 1 | | 0.711 (0.128) | 0.266 | 0.653 | 0.744 | 0.795 | 0.869 | 0.520 | 0.181 | 0.21137 | 0.15361 | 45% | 84% |
| fold 2 | | 0.712 (0.128) | 0.207 | 0.648 | 0.744 | 0.790 | 0.876 | 0.527 | 0.222 | 0.21120 | 0.15303 | 47% | 84% |
| fold 3 | | 0.709 (0.126) | 0.232 | 0.642 | 0.732 | 0.785 | 0.884 | 0.509 | 0.262 | 0.21103 | 0.15318 | 45% | 83% |
| fold 4 | | 0.708 (0.127) | 0.223 | 0.640 | 0.740 | 0.789 | 0.882 | 0.517 | 0.194 | 0.21095 | 0.15325 | 46% | 83% |
| fold 5 | | 0.711 (0.129) | 0.206 | 0.648 | 0.741 | 0.788 | 0.878 | 0.524 | 0.326 | 0.21123 | 0.15319 | 45% | 83% |
| fold 6 | | 0.711 (0.129) | 0.193 | 0.655 | 0.741 | 0.787 | 0.875 | 0.520 | 0.139 | 0.21142 | 0.15354 | 46% | 83% |
| fold 7 | | 0.708 (0.127) | 0.229 | 0.640 | 0.738 | 0.784 | 0.883 | 0.512 | 0.425 | 0.21092 | 0.15310 | 46% | 82% |
| fold 8 | | 0.705 (0.131) | 0.210 | 0.640 | 0.736 | 0.788 | 0.877 | 0.520 | 0.216 | 0.21108 | 0.15329 | 44% | 82% |
| fold 9 | | 0.710 (0.127) | 0.216 | 0.645 | 0.736 | 0.785 | 0.884 | 0.520 | 0.369 | 0.21115 | 0.15330 | 46% | 84% |
| fold 10 | | 0.709 (0.132) | 0.209 | 0.645 | 0.735 | 0.789 | 0.887 | 0.524 | 0.353 | 0.21106 | 0.15369 | 45% | 83% |
| CV Average | | 0.709 (0.129) | 0.219 | 0.646 | 0.739 | 0.788 | 0.879 | 0.519 | 0.269 | 0.21114 | 0.15332 | 45% | 83% |
|  | |  |  |  |  |  |  |  |  |  |  |  |  |
| Estimation method^a^ | | Mean (SD) | Min. | P.25 | Median | P.75 | Max. | Corr | R^2^ | RMSE | MAE | % <0.10 | % <0.25 |
| GLM (7) | |  |  |  |  |  |  |  |  |  |  |  |  |
| fold 1 | | 0.711 (0.135) | 0.158 | 0.642 | 0.737 | 0.831 | 0.999 | 0.550 | 0.224 | 0.20665 | 0.15076 | 45% | 84% |
| fold 2 | | 0.712 (0.142) | 0.115 | 0.646 | 0.745 | 0.828 | 1.028 | 0.562 | 0.306 | 0.20724 | 0.15035 | 46% | 84% |
| fold 3 | | 0.709 (0.137) | 0.119 | 0.635 | 0.732 | 0.833 | 1.095 | 0.540 | 0.198 | 0.20719 | 0.15046 | 44% | 83% |
| fold 4 | | 0.708 (0.139) | 0.123 | 0.632 | 0.737 | 0.829 | 1.008 | 0.554 | 0.149 | 0.20615 | 0.15024 | 44% | 83% |
| fold 5 | | 0.711 (0.143) | 0.099 | 0.640 | 0.741 | 0.832 | 1.098 | 0.563 | 0.101 | 0.20767 | 0.15040 | 46% | 83% |
| fold 6 | | 0.711 (0.140) | 0.091 | 0.644 | 0.742 | 0.827 | 1.000 | 0.551 | 0.283 | 0.20639 | 0.15024 | 45% | 83% |
| fold 7 | | 0.708 (0.138) | 0.138 | 0.635 | 0.730 | 0.828 | 1.142 | 0.539 | 0.208 | 0.20647 | 0.15003 | 44% | 83% |
| fold 8 | | 0.705 (0.143) | 0.120 | 0.634 | 0.733 | 0.821 | 1.007 | 0.553 | 0.364 | 0.20631 | 0.15013 | 44% | 83% |
| fold 9 | | 0.710 (0.137) | 0.120 | 0.638 | 0.738 | 0.833 | 0.966 | 0.540 | 0.445 | 0.20687 | 0.15033 | 45% | 84% |
| fold 10 | | 0.709 (0.144) | 0.112 | 0.635 | 0.741 | 0.835 | 0.990 | 0.553 | 0.223 | 0.20665 | 0.15121 | 43% | 84% |
| CV Average | | 0.709 (0.140) | 0.120 | 0.638 | 0.738 | 0.830 | 1.033 | 0.550 | 0.250 | 0.20676 | 0.15041 | 44% | 83% |
|  | |  |  |  |  |  |  |  |  |  |  |  |  |
|  | |  |  |  |  |  |  |  |  |  |  |  |  |
| Estimation method^a^ | | Mean (SD) | Min. | P.25 | Median | P.75 | Max. | Corr | R^2^ | RMSE | MAE | % <0.10 | % <0.25 |
| GLM (8) | |  |  |  |  |  |  |  |  |  |  |  |  |
| fold 1 | | 0.711 (0.139) | 0.159 | 0.636 | 0.732 | 0.816 | 1.055 | 0.566 | 0.232 | 0.20372 | 0.14997 | 48% | 85% |
| fold 2 | | 0.712 (0.145) | 0.115 | 0.643 | 0.739 | 0.816 | 1.082 | 0.573 | 0.194 | 0.20461 | 0.14928 | 45% | 85% |
| fold 3 | | 0.709 (0.142) | 0.120 | 0.633 | 0.730 | 0.818 | 1.257 | 0.557 | 0.293 | 0.20539 | 0.15005 | 47% | 84% |
| fold 4 | | 0.708 (0.143) | 0.124 | 0.631 | 0.732 | 0.818 | 1.109 | 0.565 | 0.309 | 0.20353 | 0.14936 | 47% | 85% |
| fold 5 | | 0.711 (0.147) | 0.099 | 0.637 | 0.738 | 0.824 | 1.225 | 0.574 | 0.182 | 0.20494 | 0.14913 | 48% | 84% |
| fold 6 | | 0.711 (0.144) | 0.092 | 0.638 | 0.737 | 0.818 | 1.099 | 0.565 | 0.336 | 0.20364 | 0.14928 | 48% | 83% |
| fold 7 | | 0.708 (0.142) | 0.139 | 0.632 | 0.729 | 0.812 | 1.238 | 0.555 | 0.289 | 0.20369 | 0.14960 | 48% | 84% |
| fold 8 | | 0.705 (0.147) | 0.121 | 0.630 | 0.729 | 0.810 | 1.116 | 0.567 | 0.256 | 0.20350 | 0.14946 | 47% | 84% |
| fold 9 | | 0.710 (0.142) | 0.121 | 0.637 | 0.729 | 0.819 | 1.105 | 0.558 | 0.352 | 0.20428 | 0.14964 | 48% | 84% |
| fold 10 | | 0.709 (0.149) | 0.113 | 0.635 | 0.735 | 0.815 | 1.069 | 0.569 | 0.212 | 0.20396 | 0.15042 | 46% | 84% |
| CV Average | | 0.709 (0.144) | 0.120 | 0.635 | 0.733 | 0.817 | 1.135 | 0.565 | 0.266 | 0.20413 | 0.14962 | 47% | 84% |
|  | |  |  |  |  |  |  |  |  |  |  |  |  |
| Estimation method^a^ | | Mean (SD) | Min. | P.25 | Median | P.75 | Max. | Corr | R^2^ | RMSE | MAE | % <0.10 | % <0.25 |
| GLM (9) | |  |  |  |  |  |  |  |  |  |  |  |  |
| fold 1 | | 0.711 (0.138) | 0.065 | 0.652 | 0.736 | 0.804 | 0.975 | 0.536 | 0.319 | 0.20994 | 0.15296 | 45% | 84% |
| fold 2 | | 0.712 (0.143) | 0.233 | 0.625 | 0.741 | 0.818 | 1.000 | 0.555 | 0.112 | 0.20730 | 0.15162 | 47% | 84% |
| fold 3 | | 0.709 (0.139) | 0.253 | 0.621 | 0.732 | 0.809 | 1.000 | 0.538 | 0.355 | 0.20630 | 0.15107 | 44% | 83% |
| fold 4 | | 0.708 (0.140) | 0.247 | 0.618 | 0.732 | 0.804 | 1.000 | 0.546 | 0.268 | 0.20641 | 0.15121 | 45% | 83% |
| fold 5 | | 0.711 (0.144) | 0.238 | 0.623 | 0.739 | 0.814 | 1.000 | 0.551 | 0.409 | 0.20673 | 0.15132 | 45% | 83% |
| fold 6 | | 0.711 (0.143) | 0.224 | 0.628 | 0.738 | 0.809 | 1.000 | 0.541 | 0.265 | 0.20773 | 0.15216 | 44% | 83% |
| fold 7 | | 0.708 (0.140) | 0.256 | 0.619 | 0.729 | 0.804 | 1.000 | 0.539 | 0.277 | 0.20605 | 0.15081 | 44% | 83% |
| fold 8 | | 0.705 (0.145) | 0.244 | 0.614 | 0.729 | 0.810 | 1.000 | 0.548 | 0.270 | 0.20650 | 0.15150 | 44% | 83% |
| fold 9 | | 0.710 (0.141) | 0.243 | 0.622 | 0.734 | 0.810 | 1.000 | 0.550 | 0.380 | 0.20660 | 0.15129 | 45% | 84% |
| fold 10 | | 0.709 (0.147) | 0.240 | 0.616 | 0.735 | 0.816 | 1.000 | 0.554 | 0.221 | 0.20660 | 0.15188 | 43% | 83% |
| CV Average | | 0.709 (0.142) | 0.224 | 0.624 | 0.734 | 0.810 | 0.998 | 0.546 | 0.288 | 0.20701 | 0.15158 | 45% | 83% |
|  | |  |  |  |  |  |  |  |  |  |  |  |  |
|  | |  |  |  |  |  |  |  |  |  |  |  |  |
| Estimation method^a^ | | Mean (SD) | Min. | P.25 | Median | P.75 | Max. | Corr | R^2^ | RMSE | MAE | % <0.10 | % <0.25 |
| GLM (10) | |  |  |  |  |  |  |  |  |  |  |  |  |
| fold 1 | | 0.711 (0.137) | 0.233 | 0.624 | 0.728 | 0.816 | 0.995 | 0.549 | 0.169 | 0.20563 | 0.15067 | 47% | 84% |
| fold 2 | | 0.712 (0.142) | 0.188 | 0.629 | 0.735 | 0.820 | 1.080 | 0.560 | 0.384 | 0.20633 | 0.15044 | 47% | 85% |
| fold 3 | | 0.709 (0.140) | 0.197 | 0.625 | 0.729 | 0.811 | 1.130 | 0.538 | 0.220 | 0.20629 | 0.15096 | 45% | 83% |
| fold 4 | | 0.708 (0.140) | 0.193 | 0.622 | 0.723 | 0.814 | 1.047 | 0.546 | 0.380 | 0.20616 | 0.15091 | 45% | 84% |
| fold 5 | | 0.711 (0.144) | 0.173 | 0.627 | 0.735 | 0.812 | 0.994 | 0.557 | 0.198 | 0.20665 | 0.15071 | 47% | 84% |
| fold 6 | | 0.711 (0.142) | 0.170 | 0.628 | 0.733 | 0.819 | 1.062 | 0.544 | 0.234 | 0.20688 | 0.15089 | 46% | 83% |
| fold 7 | | 0.708 (0.140) | 0.212 | 0.622 | 0.726 | 0.812 | 1.156 | 0.542 | 0.348 | 0.20594 | 0.15069 | 46% | 84% |
| fold 8 | | 0.705 (0.144) | 0.191 | 0.622 | 0.724 | 0.815 | 1.076 | 0.548 | 0.277 | 0.20604 | 0.15091 | 45% | 83% |
| fold 9 | | 0.710 (0.140) | 0.189 | 0.626 | 0.728 | 0.812 | 1.085 | 0.553 | 0.376 | 0.20620 | 0.15054 | 47% | 85% |
| fold 10 | | 0.709 (0.147) | 0.190 | 0.615 | 0.729 | 0.814 | 1.107 | 0.556 | 0.262 | 0.20622 | 0.15201 | 43% | 84% |
| CV Average | | 0.709 (0.142) | 0.193 | 0.624 | 0.729 | 0.814 | 1.073 | 0.549 | 0.285 | 0.20623 | 0.15087 | 46% | 84% |
|  | |  |  |  |  |  |  |  |  |  |  |  |  |
| Estimation method^a^ | | Mean (SD) | Min. | P.25 | Median | P.75 | Max. | Corr | R^2^ | RMSE | MAE | % <0.10 | % <0.25 |
| GLM (11) | |  |  |  |  |  |  |  |  |  |  |  |  |
| fold 1 | | 0.714 (0.123) | 0.314 | 0.631 | 0.727 | 0.811 | 0.912 | 0.543 | 0.320 | 0.20819 | 0.15112 | 47% | 85% |
| fold 2 | | 0.715 (0.122) | 0.319 | 0.635 | 0.731 | 0.814 | 0.901 | 0.546 | 0.135 | 0.20870 | 0.15048 | 48% | 85% |
| fold 3 | | 0.712 (0.121) | 0.328 | 0.629 | 0.723 | 0.813 | 0.917 | 0.531 | 0.338 | 0.20887 | 0.15101 | 47% | 84% |
| fold 4 | | 0.711 (0.122) | 0.325 | 0.630 | 0.725 | 0.813 | 0.918 | 0.533 | 0.309 | 0.20849 | 0.15083 | 47% | 84% |
| fold 5 | | 0.714 (0.123) | 0.326 | 0.632 | 0.728 | 0.815 | 0.923 | 0.543 | 0.338 | 0.20847 | 0.15047 | 48% | 84% |
| fold 6 | | 0.714 (0.122) | 0.297 | 0.638 | 0.729 | 0.811 | 0.908 | 0.539 | 0.167 | 0.20840 | 0.15079 | 47% | 84% |
| fold 7 | | 0.711 (0.122) | 0.329 | 0.629 | 0.723 | 0.806 | 0.924 | 0.529 | 0.327 | 0.20855 | 0.15082 | 47% | 84% |
| fold 8 | | 0.709 (0.124) | 0.320 | 0.627 | 0.722 | 0.808 | 0.919 | 0.540 | 0.260 | 0.20848 | 0.15096 | 47% | 84% |
| fold 9 | | 0.713 (0.121) | 0.338 | 0.630 | 0.724 | 0.813 | 0.919 | 0.538 | 0.311 | 0.20886 | 0.15093 | 47% | 85% |
| fold 10 | | 0.713 (0.125) | 0.325 | 0.626 | 0.723 | 0.808 | 0.935 | 0.544 | 0.295 | 0.20825 | 0.15136 | 46% | 85% |
| CV Average | | 0.713 (0.122) | 0.322 | 0.631 | 0.725 | 0.811 | 0.918 | 0.539 | 0.280 | 0.20852 | 0.15088 | 47% | 84% |
|  | |  |  |  |  |  |  |  |  |  |  |  |  |
|  | |  |  |  |  |  |  |  |  |  |  |  |  |
| Estimation method^a^ | | Mean (SD) | Min. | P.25 | Median | P.75 | Max. | Corr | R^2^ | RMSE | MAE | % <0.10 | % <0.25 |
| GLM B (1) | |  |  |  |  |  |  |  |  |  |  |  |  |
| fold 1 | | 0.724 (0.101) | 0.444 | 0.653 | 0.746 | 0.813 | 0.835 | 0.491 | 0.208 | 0.21698 | 0.15389 | 45% | 83% |
| fold 2 | | 0.726 (0.100) | 0.404 | 0.655 | 0.749 | 0.814 | 0.836 | 0.501 | 0.263 | 0.21728 | 0.15365 | 46% | 84% |
| fold 3 | | 0.724 (0.095) | 0.414 | 0.654 | 0.740 | 0.807 | 0.827 | 0.480 | 0.359 | 0.21777 | 0.15417 | 46% | 83% |
| fold 4 | | 0.722 (0.097) | 0.407 | 0.655 | 0.738 | 0.808 | 0.829 | 0.490 | 0.192 | 0.21737 | 0.15424 | 45% | 83% |
| fold 5 | | 0.724 (0.096) | 0.410 | 0.657 | 0.740 | 0.809 | 0.829 | 0.496 | 0.264 | 0.21755 | 0.15407 | 47% | 82% |
| fold 6 | | 0.726 (0.096) | 0.410 | 0.661 | 0.741 | 0.810 | 0.831 | 0.488 | 0.344 | 0.21758 | 0.15391 | 46% | 82% |
| fold 7 | | 0.723 (0.097) | 0.411 | 0.654 | 0.742 | 0.811 | 0.832 | 0.482 | 0.126 | 0.21768 | 0.15379 | 46% | 82% |
| fold 8 | | 0.721 (0.098) | 0.411 | 0.649 | 0.742 | 0.811 | 0.832 | 0.490 | 0.269 | 0.21768 | 0.15379 | 45% | 82% |
| fold 9 | | 0.722 (0.097) | 0.408 | 0.655 | 0.738 | 0.809 | 0.829 | 0.488 | 0.256 | 0.21740 | 0.15414 | 46% | 83% |
| fold 10 | | 0.725 (0.097) | 0.411 | 0.653 | 0.741 | 0.810 | 0.830 | 0.493 | 0.172 | 0.21761 | 0.15396 | 45% | 83% |
| CV Average | | 0.724 (0.097) | 0.413 | 0.654 | 0.741 | 0.810 | 0.831 | 0.490 | 0.245 | 0.21749 | 0.15396 | 46% | 83% |
|  | |  |  |  |  |  |  |  |  |  |  |  |  |
| Estimation method^a^ | | Mean (SD) | Min. | P.25 | Median | P.75 | Max. | Corr | R^2^ | RMSE | MAE | % <0.10 | % <0.25 |
| GLM B (2) | |  |  |  |  |  |  |  |  |  |  |  |  |
| fold 1 | | 0.724 (0.107) | 0.447 | 0.649 | 0.740 | 0.803 | 0.898 | 0.515 | 0.174 | 0.21387 | 0.15264 | 46% | 84% |
| fold 2 | | 0.726 (0.106) | 0.403 | 0.651 | 0.741 | 0.808 | 0.898 | 0.519 | 0.277 | 0.21368 | 0.15236 | 47% | 84% |
| fold 3 | | 0.723 (0.103) | 0.413 | 0.652 | 0.735 | 0.799 | 0.894 | 0.503 | 0.325 | 0.21416 | 0.15257 | 46% | 83% |
| fold 4 | | 0.722 (0.103) | 0.406 | 0.648 | 0.734 | 0.801 | 0.894 | 0.510 | 0.195 | 0.21373 | 0.15248 | 46% | 83% |
| fold 5 | | 0.724 (0.103) | 0.408 | 0.653 | 0.737 | 0.801 | 0.895 | 0.515 | 0.257 | 0.21387 | 0.15248 | 47% | 83% |
| fold 6 | | 0.725 (0.103) | 0.409 | 0.655 | 0.738 | 0.801 | 0.899 | 0.512 | 0.220 | 0.21393 | 0.15251 | 46% | 82% |
| fold 7 | | 0.722 (0.104) | 0.408 | 0.648 | 0.735 | 0.797 | 0.895 | 0.505 | 0.239 | 0.21390 | 0.15253 | 46% | 83% |
| fold 8 | | 0.721 (0.105) | 0.409 | 0.648 | 0.732 | 0.798 | 0.895 | 0.513 | 0.313 | 0.21391 | 0.15252 | 46% | 83% |
| fold 9 | | 0.722 (0.105) | 0.406 | 0.648 | 0.734 | 0.802 | 0.903 | 0.514 | 0.321 | 0.21381 | 0.15276 | 49% | 84% |
| fold 10 | | 0.725 (0.105) | 0.408 | 0.652 | 0.738 | 0.796 | 0.901 | 0.518 | 0.300 | 0.21387 | 0.15264 | 49% | 83% |
| CV Average | | 0.723 (0.104) | 0.412 | 0.650 | 0.736 | 0.801 | 0.897 | 0.512 | 0.262 | 0.21387 | 0.15255 | 47% | 83% |
|  | |  |  |  |  |  |  |  |  |  |  |  |  |
|  | |  |  |  |  |  |  |  |  |  |  |  |  |
| Estimation method^a^ | | Mean (SD) | Min. | P.25 | Median | P.75 | Max. | Corr | R^2^ | RMSE | MAE | % <0.10 | % <0.25 |
| GLM B (3) | |  |  |  |  |  |  |  |  |  |  |  |  |
| fold 1 | | 0.724 (0.102) | 0.442 | 0.652 | 0.746 | 0.811 | 0.845 | 0.493 | 0.292 | 0.21661 | 0.15362 | 46% | 83% |
| fold 2 | | 0.726 (0.101) | 0.409 | 0.652 | 0.746 | 0.810 | 0.847 | 0.503 | 0.390 | 0.21693 | 0.15341 | 47% | 84% |
| fold 3 | | 0.723 (0.097) | 0.420 | 0.654 | 0.742 | 0.803 | 0.840 | 0.483 | 0.127 | 0.21747 | 0.15340 | 46% | 83% |
| fold 4 | | 0.722 (0.098) | 0.412 | 0.652 | 0.739 | 0.801 | 0.839 | 0.492 | 0.163 | 0.21700 | 0.15345 | 46% | 83% |
| fold 5 | | 0.724 (0.097) | 0.415 | 0.656 | 0.743 | 0.809 | 0.840 | 0.499 | 0.194 | 0.21718 | 0.15336 | 46% | 82% |
| fold 6 | | 0.726 (0.097) | 0.416 | 0.655 | 0.743 | 0.804 | 0.842 | 0.491 | 0.178 | 0.21724 | 0.15336 | 46% | 82% |
| fold 7 | | 0.723 (0.098) | 0.415 | 0.652 | 0.741 | 0.804 | 0.840 | 0.484 | 0.252 | 0.21729 | 0.15324 | 45% | 82% |
| fold 8 | | 0.721 (0.099) | 0.416 | 0.650 | 0.737 | 0.801 | 0.841 | 0.491 | 0.129 | 0.21729 | 0.15324 | 48% | 85% |
| fold 9 | | 0.722 (0.099) | 0.414 | 0.650 | 0.735 | 0.802 | 0.844 | 0.491 | 0.255 | 0.21712 | 0.15362 | 46% | 83% |
| fold 10 | | 0.725 (0.098) | 0.415 | 0.651 | 0.745 | 0.803 | 0.841 | 0.495 | 0.435 | 0.21724 | 0.15329 | 46% | 83% |
| CV Average | | 0.724 (0.099) | 0.417 | 0.652 | 0.742 | 0.805 | 0.842 | 0.492 | 0.242 | 0.21714 | 0.15340 | 46% | 83% |
|  | |  |  |  |  |  |  |  |  |  |  |  |  |
| Estimation method^a^ | | Mean (SD) | Min. | P.25 | Median | P.75 | Max. | Corr | R^2^ | RMSE | MAE | % <0.10 | % <0.25 |
| GLM B (4) | |  |  |  |  |  |  |  |  |  |  |  |  |
| fold 1 | | 0.724 (0.106) | 0.449 | 0.646 | 0.740 | 0.795 | 0.884 | 0.513 | 0.311 | 0.21373 | 0.15351 | 45% | 84% |
| fold 2 | | 0.726 (0.105) | 0.398 | 0.651 | 0.739 | 0.798 | 0.882 | 0.518 | 0.193 | 0.21407 | 0.15323 | 46% | 84% |
| fold 3 | | 0.723 (0.100) | 0.407 | 0.653 | 0.739 | 0.789 | 0.876 | 0.501 | 0.354 | 0.21446 | 0.15333 | 46% | 83% |
| fold 4 | | 0.722 (0.102) | 0.401 | 0.650 | 0.738 | 0.792 | 0.878 | 0.508 | 0.261 | 0.21414 | 0.15335 | 45% | 83% |
| fold 5 | | 0.724 (0.101) | 0.403 | 0.652 | 0.741 | 0.792 | 0.880 | 0.512 | 0.298 | 0.21427 | 0.15329 | 46% | 83% |
| fold 6 | | 0.725 (0.101) | 0.403 | 0.653 | 0.740 | 0.791 | 0.882 | 0.509 | 0.289 | 0.21429 | 0.15332 | 46% | 83% |
| fold 7 | | 0.722 (0.103) | 0.404 | 0.652 | 0.735 | 0.790 | 0.882 | 0.504 | 0.189 | 0.21434 | 0.15331 | 45% | 83% |
| fold 8 | | 0.721 (0.103) | 0.404 | 0.648 | 0.733 | 0.789 | 0.881 | 0.512 | 0.240 | 0.21434 | 0.15335 | 45% | 83% |
| fold 9 | | 0.722 (0.102) | 0.400 | 0.650 | 0.738 | 0.790 | 0.882 | 0.511 | 0.258 | 0.21407 | 0.15347 | 46% | 83% |
| fold 10 | | 0.725 (0.104) | 0.403 | 0.654 | 0.737 | 0.787 | 0.885 | 0.516 | 0.242 | 0.21407 | 0.15347 | 45% | 83% |
| CV Average | | 0.723 (0.103) | 0.407 | 0.651 | 0.738 | 0.791 | 0.881 | 0.510 | 0.264 | 0.21418 | 0.15336 | 45% | 83% |
|  | |  |  |  |  |  |  |  |  |  |  |  |  |
|  | |  |  |  |  |  |  |  |  |  |  |  |  |
| Estimation method^a^ | | Mean (SD) | Min. | P.25 | Median | P.75 | Max. | Corr | R^2^ | RMSE | MAE | % <0.10 | % <0.25 |
| GLM B (5) | |  |  |  |  |  |  |  |  |  |  |  |  |
| fold 1 | | 0.724 (0.107) | 0.437 | 0.651 | 0.740 | 0.801 | 0.895 | 0.516 | 0.242 | 0.21312 | 0.15258 | 46% | 84% |
| fold 2 | | 0.726 (0.108) | 0.489 | 0.642 | 0.733 | 0.817 | 0.911 | 0.509 | 0.308 | 0.21481 | 0.15324 | 49% | 84% |
| fold 3 | | 0.724 (0.106) | 0.514 | 0.637 | 0.727 | 0.813 | 0.915 | 0.488 | 0.170 | 0.21584 | 0.15381 | 47% | 83% |
| fold 4 | | 0.722 (0.106) | 0.505 | 0.637 | 0.726 | 0.814 | 0.912 | 0.496 | 0.212 | 0.21529 | 0.15371 | 47% | 83% |
| fold 5 | | 0.724 (0.105) | 0.509 | 0.642 | 0.729 | 0.814 | 0.914 | 0.499 | 0.228 | 0.21544 | 0.15366 | 48% | 83% |
| fold 6 | | 0.726 (0.105) | 0.501 | 0.645 | 0.733 | 0.810 | 0.914 | 0.499 | 0.279 | 0.21521 | 0.15339 | 48% | 83% |
| fold 7 | | 0.723 (0.106) | 0.503 | 0.637 | 0.726 | 0.809 | 0.910 | 0.494 | 0.233 | 0.21515 | 0.15350 | 47% | 83% |
| fold 8 | | 0.721 (0.107) | 0.505 | 0.636 | 0.723 | 0.810 | 0.913 | 0.499 | 0.230 | 0.21526 | 0.15356 | 47% | 83% |
| fold 9 | | 0.722 (0.107) | 0.500 | 0.637 | 0.727 | 0.814 | 0.921 | 0.500 | 0.222 | 0.21531 | 0.15380 | 47% | 84% |
| fold 10 | | 0.725 (0.108) | 0.512 | 0.639 | 0.731 | 0.810 | 0.922 | 0.501 | 0.350 | 0.21568 | 0.15362 | 47% | 84% |
| CV Average | | 0.724 (0.106) | 0.497 | 0.641 | 0.730 | 0.811 | 0.913 | 0.500 | 0.247 | 0.21511 | 0.15349 | 47% | 83% |
|  | |  |  |  |  |  |  |  |  |  |  |  |  |
| Estimation method^a^ | | Mean (SD) | Min. | P.25 | Median | P.75 | Max. | Corr | R^2^ | RMSE | MAE | % <0.10 | % <0.25 |
| GLM B (6) | |  |  |  |  |  |  |  |  |  |  |  |  |
| fold 1 | | 0.724 (0.107) | 0.437 | 0.652 | 0.741 | 0.801 | 0.895 | 0.517 | 0.286 | 0.21311 | 0.15255 | 46% | 84% |
| fold 2 | | 0.726 (0.107) | 0.443 | 0.645 | 0.732 | 0.815 | 0.908 | 0.513 | 0.159 | 0.21440 | 0.15297 | 49% | 84% |
| fold 3 | | 0.724 (0.105) | 0.493 | 0.639 | 0.727 | 0.812 | 0.913 | 0.492 | 0.356 | 0.21548 | 0.15361 | 47% | 83% |
| fold 4 | | 0.722 (0.105) | 0.469 | 0.641 | 0.727 | 0.812 | 0.909 | 0.499 | 0.226 | 0.21486 | 0.15345 | 47% | 83% |
| fold 5 | | 0.724 (0.105) | 0.471 | 0.644 | 0.731 | 0.812 | 0.910 | 0.503 | 0.260 | 0.21497 | 0.15337 | 48% | 83% |
| fold 6 | | 0.726 (0.104) | 0.456 | 0.648 | 0.734 | 0.808 | 0.910 | 0.503 | 0.290 | 0.21476 | 0.15311 | 48% | 83% |
| fold 7 | | 0.723 (0.105) | 0.457 | 0.640 | 0.727 | 0.806 | 0.907 | 0.498 | 0.263 | 0.21474 | 0.15324 | 48% | 83% |
| fold 8 | | 0.721 (0.106) | 0.464 | 0.639 | 0.724 | 0.808 | 0.910 | 0.503 | 0.248 | 0.21486 | 0.15332 | 47% | 83% |
| fold 9 | | 0.722 (0.107) | 0.466 | 0.640 | 0.728 | 0.812 | 0.918 | 0.504 | 0.286 | 0.21488 | 0.15356 | 48% | 84% |
| fold 10 | | 0.725 (0.107) | 0.487 | 0.640 | 0.732 | 0.809 | 0.920 | 0.505 | 0.177 | 0.21528 | 0.15378 | 47% | 84% |
| CV Average | | 0.724 (0.106) | 0.464 | 0.643 | 0.730 | 0.809 | 0.910 | 0.504 | 0.255 | 0.21474 | 0.15330 | 48% | 83% |
|  | |  |  |  |  |  |  |  |  |  |  |  |  |
|  | |  |  |  |  |  |  |  |  |  |  |  |  |
| Estimation method^a^ | | Mean (SD) | Min. | P.25 | Median | P.75 | Max. | Corr | R^2^ | RMSE | MAE | % <0.10 | % <0.25 |
| GLM B (7) | |  |  |  |  |  |  |  |  |  |  |  |  |
| fold 1 | | 0.725 (0.115) | 0.351 | 0.650 | 0.743 | 0.837 | 0.918 | 0.531 | 0.161 | 0.21357 | 0.15109 | 47% | 84% |
| fold 2 | | 0.727 (0.112) | 0.406 | 0.651 | 0.736 | 0.843 | 1.238 | 0.514 | 0.190 | 0.21449 | 0.15088 | 46% | 85% |
| fold 3 | | 0.724 (0.112) | 0.406 | 0.643 | 0.734 | 0.833 | 1.071 | 0.509 | 0.198 | 0.21449 | 0.15088 | 45% | 84% |
| fold 4 | | 0.721 (0.113) | 0.393 | 0.638 | 0.731 | 0.834 | 0.996 | 0.537 | 0.260 | 0.21039 | 0.15007 | 45% | 83% |
| fold 5 | | 0.724 (0.113) | 0.412 | 0.646 | 0.741 | 0.831 | 1.078 | 0.534 | 0.213 | 0.21270 | 0.15006 | 47% | 84% |
| fold 6 | | 0.725 (0.111) | 0.356 | 0.651 | 0.740 | 0.837 | 0.951 | 0.532 | 0.227 | 0.21059 | 0.14958 | 46% | 83% |
| fold 7 | | 0.723 (0.113) | 0.397 | 0.644 | 0.737 | 0.829 | 1.062 | 0.518 | 0.236 | 0.21204 | 0.15004 | 46% | 84% |
| fold 8 | | 0.721 (0.115) | 0.395 | 0.642 | 0.733 | 0.818 | 1.001 | 0.530 | 0.225 | 0.21206 | 0.15028 | 45% | 84% |
| fold 9 | | 0.722 (0.113) | 0.389 | 0.646 | 0.736 | 0.834 | 0.992 | 0.532 | 0.189 | 0.21098 | 0.14991 | 46% | 84% |
| fold 10 | | 0.725 (0.114) | 0.399 | 0.643 | 0.741 | 0.839 | 0.946 | 0.534 | 0.288 | 0.21094 | 0.14997 | 44% | 84% |
| CV Average | | 0.724 (0.113) | 0.390 | 0.645 | 0.737 | 0.833 | 1.025 | 0.527 | 0.219 | 0.21222 | 0.15028 | 46% | 84% |
|  | |  |  |  |  |  |  |  |  |  |  |  |  |
| Estimation method^a^ | | Mean (SD) | Min. | P.25 | Median | P.75 | Max. | Corr | R^2^ | RMSE | MAE | % <0.10 | % <0.25 |
| GLM B (8) | |  |  |  |  |  |  |  |  |  |  |  |  |
| fold 1 | | 0.724 (0.119) | 0.351 | 0.647 | 0.738 | 0.824 | 0.963 | 0.547 | 0.308 | 0.21100 | 0.14979 | 50% | 85% |
| fold 2 | | 0.726 (0.119) | 0.358 | 0.651 | 0.735 | 0.827 | 1.039 | 0.552 | 0.196 | 0.20913 | 0.14799 | 51% | 85% |
| fold 3 | | 0.723 (0.117) | 0.400 | 0.641 | 0.729 | 0.827 | 1.252 | 0.523 | 0.154 | 0.21420 | 0.14991 | 48% | 85% |
| fold 4 | | 0.721 (0.117) | 0.390 | 0.639 | 0.730 | 0.822 | 1.120 | 0.546 | 0.173 | 0.20824 | 0.14871 | 49% | 84% |
| fold 5 | | 0.724 (0.117) | 0.408 | 0.643 | 0.738 | 0.823 | 1.201 | 0.543 | 0.351 | 0.21051 | 0.14899 | 50% | 84% |
| fold 6 | | 0.725 (0.115) | 0.362 | 0.647 | 0.737 | 0.822 | 1.040 | 0.545 | 0.175 | 0.20830 | 0.14836 | 49% | 84% |
| fold 7 | | 0.722 (0.117) | 0.396 | 0.646 | 0.733 | 0.820 | 1.149 | 0.532 | 0.260 | 0.20968 | 0.14904 | 49% | 84% |
| fold 8 | | 0.721 (0.118) | 0.391 | 0.642 | 0.733 | 0.817 | 1.094 | 0.542 | 0.224 | 0.20975 | 0.14894 | 49% | 84% |
| fold 9 | | 0.722 (0.118) | 0.387 | 0.642 | 0.730 | 0.820 | 1.142 | 0.548 | 0.381 | 0.20878 | 0.14870 | 49% | 85% |
| fold 10 | | 0.724 (0.118) | 0.401 | 0.642 | 0.736 | 0.823 | 1.066 | 0.548 | 0.235 | 0.20862 | 0.14881 | 48% | 85% |
| CV Average | | 0.723 (0.118) | 0.384 | 0.644 | 0.734 | 0.822 | 1.106 | 0.542 | 0.246 | 0.20982 | 0.14892 | 49% | 85% |
|  | |  |  |  |  |  |  |  |  |  |  |  |  |
|  | |  |  |  |  |  |  |  |  |  |  |  |  |
| Estimation method^a^ | | Mean (SD) | Min. | P.25 | Median | P.75 | Max. | Corr | R^2^ | RMSE | MAE | % <0.10 | % <0.25 |
| GLM B (9) | |  |  |  |  |  |  |  |  |  |  |  |  |
| fold 1 | | 0.724 (0.116) | 0.457 | 0.636 | 0.736 | 0.805 | 0.944 | 0.530 | 0.248 | 0.21070 | 0.15100 | 46% | 85% |
| fold 2 | | 0.725 (0.116) | 0.395 | 0.644 | 0.738 | 0.804 | 1.000 | 0.551 | 0.298 | 0.20846 | 0.14963 | 48% | 85% |
| fold 3 | | 0.723 (0.113) | 0.425 | 0.642 | 0.729 | 0.801 | 1.000 | 0.530 | 0.228 | 0.20939 | 0.15032 | 46% | 84% |
| fold 4 | | 0.721 (0.114) | 0.413 | 0.642 | 0.727 | 0.803 | 1.000 | 0.538 | 0.309 | 0.20891 | 0.15007 | 47% | 84% |
| fold 5 | | 0.724 (0.113) | 0.418 | 0.643 | 0.734 | 0.803 | 1.000 | 0.544 | 0.277 | 0.20894 | 0.14994 | 47% | 84% |
| fold 6 | | 0.725 (0.112) | 0.401 | 0.647 | 0.733 | 0.802 | 1.000 | 0.536 | 0.265 | 0.20868 | 0.14969 | 47% | 84% |
| fold 7 | | 0.722 (0.114) | 0.416 | 0.640 | 0.728 | 0.802 | 1.000 | 0.532 | 0.325 | 0.20890 | 0.15005 | 46% | 84% |
| fold 8 | | 0.720 | 0.423 | 0.639 | 0.728 | 0.799 | 1.000 | 0.541 | 0.311 | 0.20908 | 0.15032 | 46% | 84% |
| fold 9 | | 0.722 (0.116) | 0.408 | 0.640 | 0.727 | 0.804 | 1.000 | 0.543 | 0.283 | 0.20894 | 0.15027 | 46% | 84% |
| fold 10 | | 0.724 (0.117) | 0.424 | 0.640 | 0.730 | 0.798 | 1.000 | 0.545 | 0.322 | 0.20915 | 0.15051 | 48% | 84% |
| CV Average | | 0.723 (0.115) | 0.418 | 0.641 | 0.731 | 0.802 | 0.994 | 0.539 | 0.286 | 0.20911 | 0.15018 | 47% | 84% |
|  | |  |  |  |  |  |  |  |  |  |  |  |  |
| Estimation method^a^ | | Mean (SD) | Min. | P.25 | Median | P.75 | Max. | Corr | R^2^ | RMSE | MAE | % <0.10 | % <0.25 |
| GLM B (10) | |  |  |  |  |  |  |  |  |  |  |  |  |
| fold 1 | | 0.724 (0.116) | 0.438 | 0.640 | 0.729 | 0.813 | 0.952 | 0.534 | 0.273 | 0.21042 | 0.15093 | 47% | 85% |
| fold 2 | | 0.725 (0.409) | 0.409 | 0.645 | 0.730 | 0.811 | 1.038 | 0.547 | 0.276 | 0.20937 | 0.15003 | 49% | 85% |
| fold 3 | | 0.723 (0.113) | 0.432 | 0.642 | 0.729 | 0.807 | 1.046 | 0.524 | 0.316 | 0.21005 | 0.15050 | 47% | 84% |
| fold 4 | | 0.721 (0.114) | 0.395 | 0.641 | 0.726 | 0.804 | 0.988 | 0.537 | 0.151 | 0.20877 | 0.15001 | 48% | 84% |
| fold 5 | | 0.724 (0.115) | 0.416 | 0.644 | 0.731 | 0.805 | 1.033 | 0.540 | 0.282 | 0.20970 | 0.15035 | 48% | 84% |
| fold 6 | | 0.725 (0.113) | 0.386 | 0.647 | 0.729 | 0.809 | 0.991 | 0.537 | 0.292 | 0.20892 | 0.14958 | 48% | 84% |
| fold 7 | | 0.722 (0.115) | 0.425 | 0.639 | 0.729 | 0.803 | 1.013 | 0.531 | 0.277 | 0.20927 | 0.15008 | 47% | 83% |
| fold 8 | | 0.721 (0.115) | 0.411 | 0.638 | 0.727 | 0.806 | 0.973 | 0.536 | 0.282 | 0.20925 | 0.14995 | 47% | 84% |
| fold 9 | | 0.722 (0.393) | 0.393 | 0.642 | 0.727 | 0.806 | 1.001 | 0.541 | 0.282 | 0.20886 | 0.15002 | 48% | 85% |
| fold 10 | | 0.724 (0.117) | 0.427 | 0.640 | 0.727 | 0.808 | 1.040 | 0.541 | 0.267 | 0.20972 | 0.15079 | 45% | 84% |
| CV Average | | 0.723 (0.115) | 0.413 | 0.642 | 0.728 | 0.807 | 1.008 | 0.537 | 0.270 | 0.20943 | 0.15022 | 47% | 84% |
|  | |  |  |  |  |  |  |  |  |  |  |  |  |
|  | |  |  |  |  |  |  |  |  |  |  |  |  |
| Estimation method^a^ | | Mean (SD) | Min. | P.25 | Median | P.75 | Max. | Corr | R^2^ | RMSE | MAE | % <0.10 | % <0.25 |
| GLM B (11) | |  |  |  |  |  |  |  |  |  |  |  |  |
| fold 1 | | 0.725 (0.113) | 0.388 | 0.641 | 0.729 | 0.822 | 0.932 | 0.523 | 0.169 | 0.21256 | 0.15107 | 49% | 85% |
| fold 2 | | 0.726 (0.113) | 0.394 | 0.645 | 0.735 | 0.822 | 0.915 | 0.529 | 0.256 | 0.21246 | 0.15044 | 50% | 85% |
| fold 3 | | 0.724 (0.111) | 0.414 | 0.640 | 0.724 | 0.825 | 0.939 | 0.513 | 0.215 | 0.21293 | 0.15066 | 49% | 85% |
| fold 4 | | 0.722 (0.110) | 0.431 | 0.637 | 0.726 | 0.810 | 0.939 | 0.515 | 0.303 | 0.21236 | 0.15067 | 48% | 85% |
| fold 5 | | 0.724 (0.110) | 0.410 | 0.641 | 0.731 | 0.815 | 0.936 | 0.521 | 0.179 | 0.21279 | 0.15066 | 49% | 84% |
| fold 6 | | 0.726 (0.111) | 0.390 | 0.646 | 0.730 | 0.817 | 0.926 | 0.519 | 0.314 | 0.21227 | 0.15039 | 49% | 84% |
| fold 7 | | 0.723 (0.111) | 0.409 | 0.640 | 0.726 | 0.811 | 0.945 | 0.512 | 0.320 | 0.21256 | 0.15057 | 48% | 84% |
| fold 8 | | 0.721 (0.113) | 0.406 | 0.638 | 0.724 | 0.813 | 0.940 | 0.521 | 0.264 | 0.21260 | 0.15083 | 48% | 84% |
| fold 9 | | 0.722 (0.113) | 0.409 | 0.636 | 0.723 | 0.819 | 0.944 | 0.522 | 0.259 | 0.21277 | 0.15086 | 48% | 85% |
| fold 10 | | 0.725 (0.113) | 0.425 | 0.636 | 0.728 | 0.819 | 0.960 | 0.522 | 0.313 | 0.21257 | 0.15104 | 47% | 84% |
| CV Average | | 0.724 (0.112) | 0.407 | 0.640 | 0.728 | 0.817 | 0.938 | 0.520 | 0.259 | 0.21259 | 0.15072 | 48% | 85% |

^a^ Estimation method: CLAD = censored least absolute deviations; OLS = ordinary least squares; GLM = generalised linear model; and BB = Beta Binomial.

The dependent variables in the rest of the regression models were EQ-5D-5L utilities. The independent variable(s) were: Model (1) Functional Outcomes of Sleep Questionnaire (FOSQ-10) summary score only; Model (2) FOSQ-10 summary score, OSA status and income level; Model (3) FOSQ-10 summary score and OSA status; Model (4) FOSQ-10 summary score and income level; Model (5) FOSQ-10 summary score, FOSQ-10 summary score squared, OSA status and income level; Model (6) FOSQ-10 summary score, FOSQ-10 summary score cubed, OSA status and income level; Model (7) FOSQ-10 domain scores (F_gp_conc: concentration, F_gp_rem: remembering, F_al_ra: relationship affected, F_al_am: morning activity, F_al_pm: evening activity, F_vi_sd: driving short distance, F_vi_ld: driving long distance, F_vi_mo: watching movies, F_social: social visit, F_sex: desire intimacy); Model (8) FOSQ-10 domain scores, OSA status and income level; (9) FOSQ-10 summary score^-2^, FOSQ-10 summary score^-0.5^, income, OSA, education and four FOSQ-10 sub domain items were included (i.e. F_gp_conc^3^, F_al_am, F_al_pm, F_sex); (10) FOSQ-10 summary score^-2^, FOSQ-10 summary score^-0.5^, income, OSA, education and five FOSQ-10 sub domain items were included (i.e. F_gp_conc, F_al_am^3^, F_al_pm, F_social^-2^, F_sex); (11) FOSQ10 1.F_gp_conc 4.F_gp_conc 1.F_gp_rem 1.F_al_ra 4.F_al_am 1.F_al_pm 4.F_al_pm 1.F_vi_sd 2.F_vi_ld 3.F_vi_mo 1.F_social 4.F_social 4.F_sex 0.biincomelevel 0.OSA. ^*^ Estimation samples used k-1 = 9 cross validated samples (n=1,362), and the validation sample is formed by the leftover fold (k – 9 = 1, n =152).

For all models, FOSQ-10 calculated as a continuous variable with a range of 0 – 20; EQ-5D-5L utilities calculated as continuous variables ranging from -0.594 to 1, except for Beta regression where negatives values are replaced to zero; OSA: dummy = 1 if diagnosed with OSA and 0 if undiagnosed;^c^ Income: dummy = 1 if earning more than AU$ 60,000/ year and 0 if lower than AU$ 60,000/year; Education: dummy =1 if attaining education higher than high-school/ Year 12 level and 0 if not finished high-school/ Year 12 level.

EQ-5D-5L EuroQoL 5 dimensions 5 levels, FOSQ-10 Functional Outcomes of Sleep Questionnaire

SD = Standard deviation; min = minimum; Max = maximum; Corr = spearman rank correlation; RMSE = Root mean squared error; MAE = Mean absolute error; % <0.10 and % <0.25 total proportion of all predictions (across both the estimation and validation samples) where the predicted utility score differed from the observed value by no more than 0.10 or 0.25 respectively.

**Table S2** Predictive ability of best fitting models when gender, area and age group dummies are included as explanatory variables

| Estimation method^a^ | RMSE | MAE | Estimation method^b^ | RMSE | MAE | Estimation method^a^ | RMSE | MAE | Estimation method^b^ | RMSE | MAE |
| --- | --- | --- | --- | --- | --- | --- | --- | --- | --- | --- | --- |
| Estimation samples* | |  | Estimation samples* | |  | Validation samples* | |  | Validation samples* | |  |
| BB (9) | 0.1997 | 0.1433 | BB (9) | 0.2017 | 0.1434 | BB (9) | 0.1902 | 0.1430 | BB (9) ^b^ | 0.2001 | 0.1435 |
| OLS (9) | 0.2017 | 0.1487 | OLS (9) ^b^ | 0.2043 | 0.1508 | BB (10) | 0.1898 | 0.1439 | BB (10) ^b^ | 0.2055 | 0.1504 |
| OLS (10) | 0.2021 | 0.1470 | OLS (10) ^b^ | 0.2033 | 0.1484 | BB (11) | 0.1924 | 0.1456 | BB (11) ^b^ | 0.2022 | 0.1459 |
| OLS (7) | 0.2041 | 0.1484 | OLS (7) ^b^ | 0.2071 | 0.1498 | BB (8) | 0.1936 | 0.1454 | BB (8) ^b^ | 0.2039 | 0.1493 |
| GLM (8) | 0.2041 | 0.1496 | GLM (8) ^b^ | 0.2049 | 0.1508 | BB (5) | 0.1937 | 0.1458 | BB (5) ^b^ | 0.2030 | 0.1468 |

^a^ Estimation method: OLS = ordinary least squares; GLM = generalised linear model; and BB = Beta Binomial.

The dependent variables in the rest of the regression models were EQ-5D-5L utilities. The independent variable(s) were: Model (5) FOSQ-10 summary score, FOSQ-10 summary score squared, OSA status and income level; Model (7) FOSQ-10 domain scores (F_gp_conc: concentration, F_gp_rem: remembering, F_al_ra: relationship affected, F_al_am: morning activity, F_al_pm: evening activity, F_vi_sd: driving short distance, F_vi_ld: driving long distance, F_vi_mo: watching movies, F_social: social visit, F_sex: desire intimacy); Model (8) FOSQ-10 domain scores, OSA status and income level; (9) FOSQ-10 summary score^-2^, FOSQ-10 summary score^-0.5^, income, OSA, education and four FOSQ-10 sub domain items were included (i.e. F_gp_conc^3^, F_al_am, F_al_pm, F_sex); (10) FOSQ-10 summary score^-2^, FOSQ-10 summary score^-0.5^, income, OSA, education and five FOSQ-10 sub domain items were included (i.e. F_gp_conc, F_al_am^3^, F_al_pm, F_social^-2^, F_sex); (11) FOSQ10 1.F_gp_conc 4.F_gp_conc 1.F_gp_rem 1.F_al_ra 4.F_al_am 1.F_al_pm 4.F_al_pm 1.F_vi_sd 2.F_vi_ld 3.F_vi_mo 1.F_social 4.F_social 4.F_sex 0.biincomelevel 0.OSA.

^b^ Estimation method is the same as the first with additional explanatory variables including: gender (dummy =1 if male and 0 if female), age group (dummy = 1 if < 55 years and 0 if > 55 years), and area (dummy = 1 if regional and 0 if metro).

EQ-5D-5L EuroQoL 5 dimensions 5 levels, FOSQ-10 Functional Outcomes of Sleep Questionnaire

RMSE = Root mean squared error; MAE = Mean absolute error

^*^ Estimation samples used k-1 = 9 cross validated samples (n=1,362), and the validation sample is formed by the leftover fold (k – 9 = 1, n =152).

**Table S3** Predicting utilities of EQ-5D-5L from FOSQ-10 using mapping algorithm BB 9

| Item no. | Item names and formula (A) | Example Item values (B) | Item transformed values (C= B🡪A)^a^ | Betareg coefficient (D) | Products  (E = C * D) |
| --- | --- | --- | --- | --- | --- |
| Item 1 | (FOSQ10 total)^-2^-0.33 | 17.67 | -0.3265 | 1.63E-11 | -5.32E-12 |
| Item 2 | ((FOSQ10 total+4.77*10^-7^)/10)^-0.5^-0.76 | 17.67 | -0.0054 | -1.5640 | 0.0084 |
| Item 3 | Income level | 0 | 0 | 0.2376 | 0 |
| Item 4 | OSA diagnostic status | 0 | 0 | -0.1777 | 0 |
| Item 5 | Education level | 1 | 1 | 0.1929 | 0.1929 |
| Item 6 | (Concentration+1)^3^-79.65 | 3 | -16.2459 | 0.0046 | -0.0747 |
| Item 7 | (Morning activity)-3.35 | 3 | -0.3500 | 0.1351 | -0.0473 |
| Item 8 | (Evening activity)-3.26 | 4 | 0.7381 | 0.1746 | 0.1289 |
| Item 9 | (Intimacy level)-2.66 | 4 | 1.3441 | 0.0704 | 0.0946 |
|  |  |  |  | Constant | 0.7170 |
|  |  |  |  | Sum | 1.0198 |
|  | EQ-5D-5L utility | 0.7950 |  | Logit transformation^c^ | 0.7349 |

^a^ The Item transformed values (C) are derived from placing example item values (B) to the item names and formula (A).

^b^ The products (E) are obtained by multiplying Item transformed values (A) by Betareg coefficient (D). The betareg coefficients are reported in Table 5.

^c^ The logit transformation for BB 9 is achieved by using the following formula: exp(sum) / (1+exp(sum); where sum is the summation of the products in column E with the constant term of 0.717.

A randomly selected case from the estimation sample with FOSQ-10 total score of 17.67, earning less than AU$60,000/year (income level = 0), not diagnosed with OSA (OSA status = 0), completed high-school (education level = 0), and has specific FOSQ-10 domain scores of 3 for concentration and morning activity, and 4 for evening activity and intimacy level has an original EQ-5D-5L utility of 0.795, which is comparable to the predicted EQ-5D-5L utility derived from the mapping algorithm of 0.735.

**Table S4** Marginal effect for the highest rank model (BB 9) based on the whole sample (n=1,514)

| FOSQ-10 domain item/ demographic characteristics | Margin effect (standard error) |
| --- | --- |
| (FOSQ 10 total score)^-2^ | 0.000 (0.000)*** |
| (FOSQ 10 total score)^-0.5^ | -0.301 (0.085)*** |
| OSA status | -0.034 (0.010)*** |
| Income level | 0.050 (0.010)*** |
| Education level | 0.037 (0.010)*** |
| (Concentration)^3^ | 0.001 (0.000)*** |
| Morning activity | 0.026 (0.007)*** |
| Evening activity | 0.034 (0.007)*** |
| Intimacy | 0.014 (0.003)*** |

FOSQ-10 Functional Outcomes of Sleep Questionnaire, OSA Obstructive Sleep Apnoea

* Statistically significant at 0.1 level, ** statistically significant at 0.05 level, *** statistically significant at 0.01 level
